# Mapping applications and evaluations of LLM-enabled AI chatbots for health purposes: a scoping review

**DOI:** 10.3389/fpubh.2026.1916780

**Published:** 2026-09-22

**Authors:** Yuan Wang, Romy RW, Yanjiao Deng, Xingyu Chen

**Affiliations:** 1 Wee Kim Wee School of Communication and Information, Nanyang Technological University, Singapore, Singapore; 2 College of Communication and Fine Arts, Loyola Marymount University, Los Angeles, CA, United States; 3 School of Journalism and Information Communication, Huazhong University of Science and Technology, Wuhan, China

**Keywords:** artificial intelligence, delivery of health care, digital public health, health communication, large language model, scoping review

## Abstract

**Background:**

Large Language Model (LLM)-enabled artificial intelligence (AI) chatbots are increasingly shaping health communication by mediating how patients, health professionals, researchers, and health institutions seek, interpret, produce, and act on health information. Existing reviews have largely focused on a single stakeholder group or clinical domain, leaving unclear how these systems are applied and evaluated across stakeholder groups and health purposes.

**Objective:**

This scoping review mapped empirical studies of LLM-enabled AI chatbot applications and evaluations for health purposes across four stakeholder groups: the public or patients, health professionals, health researchers and students, and health institutions. We characterized the purposes and evidence patterns associated with these applications and evaluations.

**Methods:**

We searched nine databases for peer-reviewed, English-language empirical studies available through July 2025. After screening, 286 articles were coded for study characteristics, methodology, evidence type, chatbot modality, LLM type, health topic, stakeholder group, and health purpose.

**Results:**

The included articles were published between 2023 and 2025. Most examined general-purpose LLMs (*n* = 267, 93.4%). Studies were concentrated in the United States and China and primarily examined text-based chatbots. Noncommunicable or chronic diseases and general health information were the most common health topics. Ten health-related purposes were identified across four stakeholder groups and two umbrella categories. Public-facing applications involved the general public or patients and encompassed four purposes: (1) seeking health information, (2) symptom assessment and self-care management, (3) emotional support, and (4) preventive and transitional care support. Professional and organizational applications involved health professionals, health researchers and students, and health institutions, and encompassed six purposes: (5) clinical decision support, (6) improving administrative efficiency, (7) supporting patient interaction and doctor–patient communication, (8) enhancing continuing education, (9) facilitating health research, and (10) institutional workflow support and care navigation.

**Conclusion:**

This review provides a stakeholder–purpose mapping of AI chatbot applications and evaluations in health care. The evidence base is concentrated in text-based, lower-acuity, and information-oriented contexts and derives primarily from output evaluations and self-reported perceptions or use. Key gaps concern institutional integration and governance, higher-stakes and longitudinal contexts, and evidence across populations, languages, and interaction modalities.

## 1 Introduction

### 1.1 Background

Artificial intelligence (AI)-powered chatbots are increasingly transforming the delivery of and access to health care. AI chatbots refer to conversational software agents that use AI methods to interact with patients, clinicians, or other stakeholders via natural language (1, 2). Earlier generations of health chatbots were largely rule-based, following scripted dialog trees or matching user inputs against predefined knowledge bases. Following the public release and rapid adoption of generative AI systems such as ChatGPT in late 2022, chatbots powered by large language models (LLMs) have emerged as a prominent form of conversational AI in health care. This review focuses specifically on LLM-enabled AI chatbots, which we define as user-facing conversational systems in which an LLM generates or substantially contributes to the generation of natural-language responses to user inputs. The term “AI chatbots” is used hereafter to refer to these LLM-enabled systems.

In recent years, scholarly attention to AI chatbots and their potential applications in health care has expanded substantially. These chatbots have been examined across diverse health purposes, ranging from mental health support (3) and chronic disease management (4) to clinical decision support (5). This expansion is situated within a broader health chatbot market that has also experienced substantial growth. According to Fortune Business Insights (6), the global health care chatbot market was valued at USD 1.98 billion in 2025 and is projected to grow from USD 2.41 billion in 2026 to USD 12.63 billion by 2034, representing a compound annual growth rate of 23.01%.

Health care stakeholders at multiple levels report growing exposure to and interest in AI chatbots. At the institutional level, a 2025 World Health Organization survey found that 64% of countries in the European Region were using AI-assisted diagnostics, and 50% had introduced AI chatbots for patient engagement and support (7). Among clinicians, a survey of 498 physicians in Germany found that 32.1% reported using AI for medical purposes and 81.5% perceived AI as useful for preventive diagnostics (8). Among the public, a population-based survey conducted in Denmark in 2024 found that approximately 35% of respondents believed that the benefits of AI in health care outweighed the risks (9). In more targeted domains such as mental health, a 2025 survey suggested that approximately one in eight U. S. adolescents and young adults aged 12–21 reported using AI chatbots for mental health advice (10). Collectively, these findings indicate growing institutional and public engagement with health-related AI, although chatbot-specific use remains unevenly documented across stakeholder groups.

Despite the growing attention to the diverse needs and applications of AI chatbots, there is a lack of systematic reviews mapping how specific stakeholders utilize these tools for distinct purposes. Previous reviews have predominantly concentrated on a single dimension of chatbot application, focusing either on one stakeholder group (e.g., consumers (11)) or a specific health domain (e.g., cancer therapy (12)). While broader reviews (13) have synthesized evidence on the benefits and limitations of health care chatbots across clinical and public health contexts, few have systematically examined how different stakeholder groups use chatbots for distinct purposes. Moreover, much of this earlier evidence predates the widespread adoption of large language models and therefore centers on rule-based health chatbots. Consequently, prior research captures important segments of the field, but an integrated, multi-stakeholder synthesis of empirical research on AI chatbots remains lacking.

A scoping review mapping the applications and evaluations of AI chatbots across stakeholders and health purposes is therefore needed. Evidence from digital health communication research suggests that stakeholder groups engage with technologies with different goals and constraints (14). Recent research on human–AI communication further suggests that responses to AI chatbots are shaped not only by system capabilities but also by how users perceive AI-mediated interactions (15–18). Without a structured synthesis that links stakeholder groups to specific health purposes and domains, it remains difficult to assess where evidence is concentrated, where critical gaps exist, and how future research, design, and governance efforts should be prioritized. This review aims to address this gap by mapping empirical studies of AI chatbot applications and evaluations across stakeholder groups and health purposes.

In this review, we adapted a previous stakeholder framework (14) and classified stakeholders into four categories, organized within two application-level groups. Public-facing applications involve (1) *the general public or patients*, that is, lay individuals interacting with AI chatbots outside a professional role. Professional and organizational applications involve (2) *health professionals*, including clinicians and allied health practitioners acting in a professional capacity; (3) *health researchers and students*, including academic researchers, educators, and trainees; and (4) *health institutions*, defined as organizational actors such as hospitals and health systems. This framework distinguishes stakeholder groups whose goals, forms of use, and risk profiles differ.

## 2 Objectives

Given the rapid proliferation of AI chatbots in health-related contexts and their expanding range of potential applications across diverse stakeholders, there is a growing need to synthesize how these systems are being empirically studied and applied for health purposes. To address this need, this scoping review aims to map the empirical literature on AI chatbot applications and evaluations across health topics and across different stakeholder groups, including the general public or patients, health professionals, health researchers and students, and health institutions.

Accordingly, this review is guided by the following research questions:

*RQ1*: What are the general characteristics of existing empirical studies examining the applications and evaluations of AI chatbots for health purposes (e.g., publication trends, geographic distribution, study designs, health topics, chatbot modalities, and LLM types)?

*RQ2*: What applications and evaluations of AI chatbots have been empirically examined for each stakeholder group (general public or patients, health professionals, health researchers and students, and health institutions)?

By synthesizing evidence from a diverse and rapidly growing body of literature, this review provides researchers, health professionals, and policymakers with a structured overview of how AI chatbots are currently studied and utilized across health care contexts. In doing so, it lays a foundation for identifying research gaps, guiding future empirical investigations, and informing the governance of AI chatbot applications in health care.

## 3 Methods

A scoping review design was selected because the objective was to map the breadth, characteristics, and types of evidence within a rapidly evolving and heterogeneous field. This scoping review followed the Preferred Reporting Items for Systematic Reviews and Meta-Analyses extension for Scoping Reviews (PRISMA-ScR) guidelines (19) for literature searching, study selection, and reporting. The search and screening process is illustrated in Figure 1.

**Figure 1:**
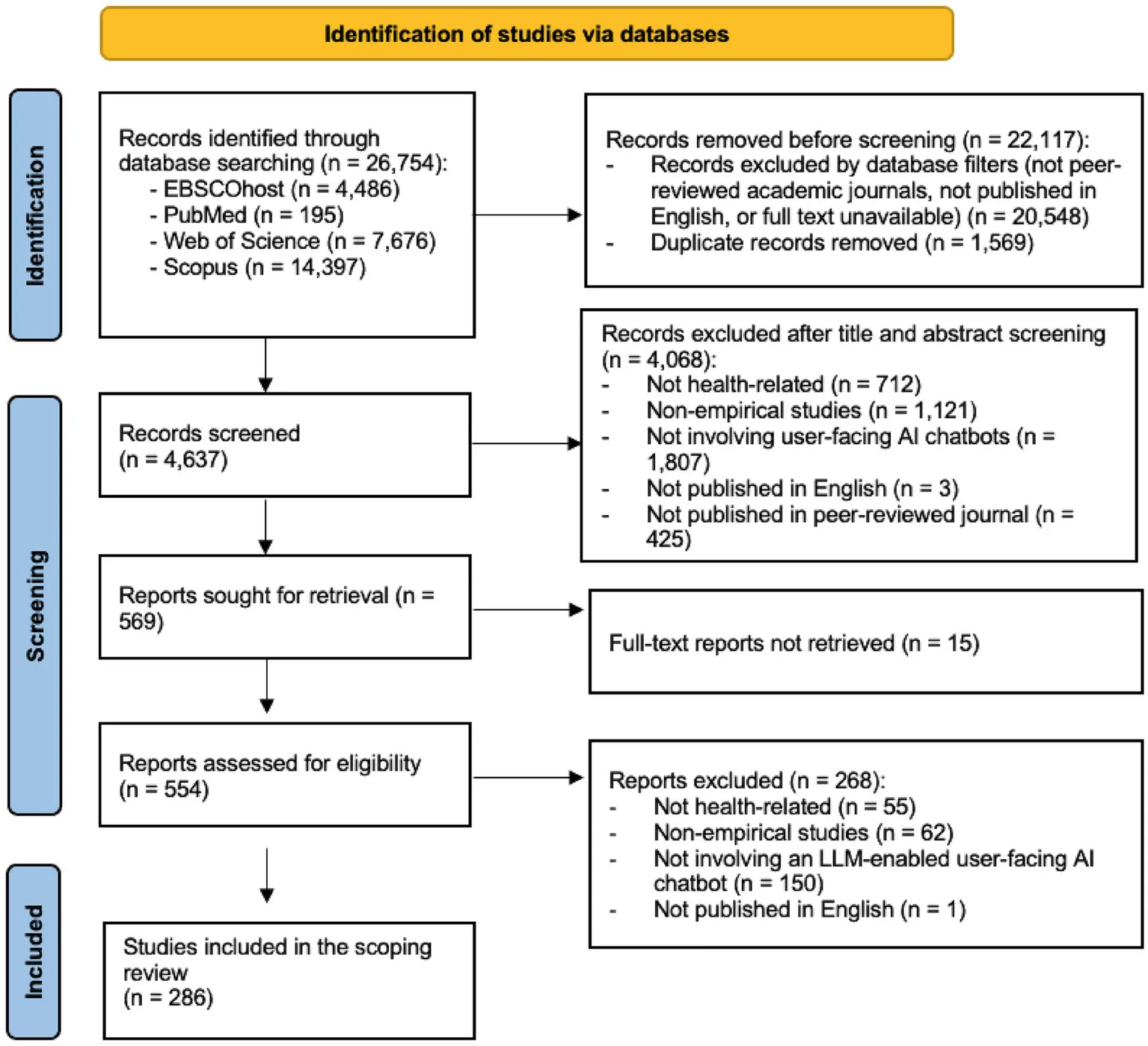
PRISMA flow diagram.

### 3.1 Search strategy

A systematic literature search was conducted to identify empirical research articles examining the use of LLM-enabled AI chatbots for health-related purposes. Searches were performed across multiple databases to ensure comprehensive coverage of health, medical, communication, information science, and interdisciplinary research. Specifically, we searched a total of nine databases, including six databases accessed through EBSCOhost (*Academic Search Complete, Communication and Mass Media Complete, Global Health, Library Literature and Information Science, APA PsycInfo, and Education Source Ultimate*), as well as PubMed, Web of Science, and Scopus.

The search strategy combined AI-related terms with health-related terms using Boolean operators. The AI-related concept block comprised “generative artificial intelligence,” “generative AI,” “AI-generated content,” AIGC, GenAI, “large language model*,” chatbot, ChatGPT, DeepSeek, and Midjourney. The health-related concept block comprised health*, healthcare, medicine, medical, clinic*, patient*, hospital*, doctor*, “public health,” “chronic disease*,” and “mental health.” The search syntax and field tags were adapted for each database. The complete database-specific search strings and filters are provided in Supplementary Table S3.

Where available, additional filters were applied to restrict results to peer-reviewed academic journal articles written in English and available in full text. All database searches and study-selection procedures were completed in July 2025. Some included articles were retrieved as online-first versions and later assigned to formal journal volumes and issues; consequently, their recorded publication dates may postdate the search closure, although the versions screened and coded fell within the search window. The initial search yielded 26,754 records. After applying database filters, 6,206 records remained.

### 3.2 Screening criteria

We screened articles based on the following criteria. First, the purpose of this review was to map how LLM-enabled AI chatbots are applied and evaluated for health-related purposes across stakeholder groups. Therefore, articles that did not involve a health-related context or addressed health topics only tangentially were excluded. Second, because the focus of this review was on user-facing applications and evaluations of LLM-enabled AI chatbots, we excluded purely technical articles (e.g., system development or algorithmic design) and studies of non-LLM conversational agents (e.g., rule-based, flow-based, or convolutional neural network-based systems). Third, to ensure the inclusion of evidence-based findings, only empirical research articles reporting original data (e.g., surveys, experiments, interviews, content analyses) were included. Conceptual papers, commentaries, editorials, opinion pieces, study protocols, and purely technical or algorithmic articles were excluded. Finally, to maintain scholarly rigor and comparability across articles, only articles published in peer-reviewed academic journals and written in English were included. No protocol was prospectively registered or publicly posted for this scoping review. The full search strategy and coding framework are provided in Supplementary Tables S2, S3.

### 3.3 Screening process

After removing duplicates across databases (*n* = 1,569), all remaining records (*n* = 4,637) were screened in two stages: title and abstract screening, followed by full-text review. In the first stage, titles and abstracts were screened to exclude clearly ineligible articles. Articles were excluded for the following reasons: not situated in a health-related context (*n* = 712), non-empirical in nature (*n* = 1,121), not involving a user-facing AI chatbot application or evaluation (*n* = 1,807), not published in English (*n* = 3), or not published in a peer-reviewed journal (*n* = 425). Following this initial screening, 569 articles were retained for full-text review.

In the second stage, reports were sought for retrieval for 569 records, of which 15 were not retrieved. The remaining 554 reports were assessed for eligibility, and 268 were excluded because they were not situated in a health-related context (*n* = 55), non-empirical in nature (*n* = 62), not involving an LLM-enabled user-facing AI chatbot (*n* = 150), or not published in English (*n* = 1). Following full-text screening, 286 articles were retained for final coding and analysis.

### 3.4 Coding process

All included articles (*N* = 286) were coded using a structured codebook. The unit of analysis in this review was the published article. Although some articles employed mixed-methods designs, each article was treated as a single analytical unit, consistent with the review’s mapping-oriented objective of examining how AI chatbots are applied and evaluated for health-related purposes across stakeholders.

A preliminary coding scheme was developed based on an initial review of approximately 15% of the included articles. Using this preliminary scheme, a subset of articles (*n* = 30, 10.5%) was independently coded by two researchers. Intercoder reliability was satisfactory across coding dimensions (Krippendorff’s *α* = 0.81–1.00). For five dimensions with no variation, α was not estimable and percent agreement was 100% (Supplementary Table S2). For discrepant items, all members of the research team jointly reviewed the relevant article, refined the applicable coding rule where necessary, and reached consensus on the final code.

Each included article was first coded for basic article characteristics, including *year of publication*, *human involvement, country of research participants*, *sample size*, and the *primary health issue* addressed (e.g., communicable and infectious diseases, noncommunicable or chronic diseases, mental health, maternal, sexual, and reproductive health, nutrition- and lifestyle-related health issues, general health information, or other). Human involvement recorded the analytic role of people in each study: none, evaluator or comparator only (people took part solely to rate chatbot outputs or to produce the material used for comparison), or participant (people were themselves an object of measurement). Country of participants and sample size were recorded only for articles coded as participant.

Next, the *chatbot modality* examined in each article was coded (e.g., text-based, voice-based, or multimodal). Chatbot modality was coded based on the type of interaction examined; articles were coded as text-based when they analyzed only textual interactions, even if the chatbot system itself was technically capable of multimodal output. *LLM type* was coded as general-purpose, custom, or unspecified. General-purpose LLMs referred to broadly available chatbots designed for use across domains, whereas custom LLM applications were built or adapted for a specific health task or user group. Systems were coded as unspecified when the article provided insufficient information for classification. Where reported, we also recorded the specific chatbot name (e.g., ChatGPT).

*Methodological approach* was coded using multiple-response options to account for articles employing mixed-methods designs (e.g., experiment, survey, interview, content analysis, text analysis). We also coded *evidence type* to indicate whether the evidence reflected actual use, self-reported use, or potential use. Evidence types formed an ordered gradient of proximity to actual use: (1) *output evaluation*, in which researchers assessed chatbot-generated outputs or task performance; (2) *self-reported perceptions and use,* in which participants reported attitudes, intentions, or prior/current use; (3) *researcher-directed interaction*, in which participants interacted with a chatbot as specified by the study protocol; and (4) *documented naturalistic use*, in which personal, clinical, organizational, or scholarly use occurring outside researcher direction was evidenced by logs, records, interaction traces, or direct observation. Types 1–2 primarily characterize potential or reported use, whereas types 3–4 provide direct evidence of actual engagement.

Each article was then coded for one or more of the four stakeholder groups defined above: the general public or patients, health professionals, health researchers and students, and health institutions. The purpose framework was developed inductively through review of approximately 15% of the included articles and subsequently refined during pilot coding based on conceptual distinctiveness and applicability across study designs. Both dimensions permitted multi-label coding: articles involving multiple stakeholder groups or purposes were coded for all applicable categories. Because stakeholder and purpose were coded independently, a given purpose could involve more than one stakeholder group (e.g., continuing education involving both practicing professionals and students in training). The complete coding results are provided in Supplementary Table S1.

## 4 Descriptions of research characteristics

### 4.1 Publication trends and sample characteristics

Our review included 286 articles, all published between 2023 and 2025. Publication volume rose sharply after 2023, with 39 articles (13.6%) published in 2023, 125 articles (43.7%) in 2024, and 122 articles (42.7%) in 2025 (through July), indicating accelerated scholarly attention following the public release of ChatGPT in late 2022.

Of the 286 included articles, 149 (52.1%) involved people as participants, 85 (29.7%) involved people only as evaluators or raters of chatbot outputs, and 52 (18.2%) involved no people. Among the 149 participant studies, sample sizes ranged from 1 to 2,406 (median = 101; interquartile range = 21–306). Seventy-three studies (49.0%) enrolled fewer than 100 participants, indicating that much of the participant-level evidence was based on small samples.

In terms of geographic distribution, the United States (*n* = 46) and China (*n* = 26) were the most frequently represented contexts among the 149 participant studies, followed by the United Kingdom (*n* = 13) and Germany, Saudi Arabia, and Turkey (*n* = 11 each). Eighteen studies (12.1%) involved participants from multiple countries, and six (4.0%) did not report an individually named participant country. Overall, participant research was geographically diverse but concentrated primarily in North America, Western Europe, East Asia, and the Middle East. Figure 2 presents the geographic distribution of participant studies.

**Figure 2:**
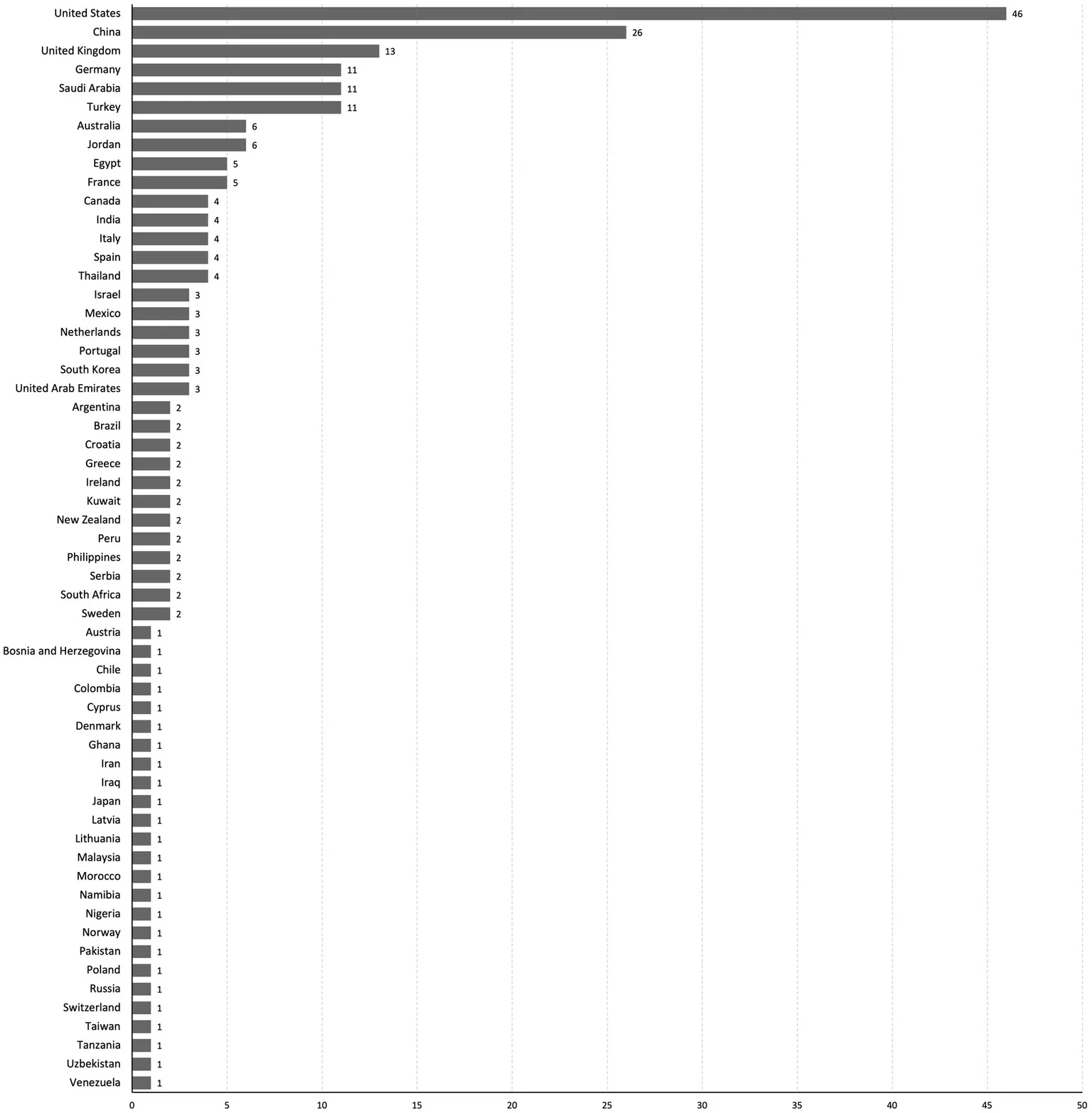
Geographic distribution of participant studies (*n* = 149). Bars indicate the number of included studies involving participants from each reported country. Multicountry studies were counted once for each named country; therefore, country counts were not mutually exclusive. Studies for which participant countries were not individually reported were not assigned to a specific country.

### 4.2 Methodological approaches

Regarding research methods, 195 studies (68.2%) employed a single approach, whereas 91 (31.8%) employed two or more methodological approaches. Among studies using a single approach (*n* = 195), content analysis was the most common (*n* = 112, 57.4%), followed by survey (*n* = 47, 24.1%), experiment (*n* = 19, 9.7%), interview (*n* = 12, 6.2%), and focus group (*n* = 5, 2.6%). Among studies using multiple approaches (*n* = 91), the most common exact combinations were text analysis with content analysis (*n* = 28, 30.8%), experiment with content analysis (*n* = 18, 19.8%), and survey with content analysis (*n* = 11, 12.1%).

### 4.3 LLM types and Chatbot models

Most studies focused on general-purpose LLMs (*n* = 267, 93.4%), followed by custom LLM applications (*n* = 15, 5.2%) and studies in which the LLM type was unspecified (*n* = 4, 1.4%). Among named specific chatbot systems, ChatGPT was the most frequently reported (*n* = 252, 88.1%). Google Bard or Gemini was reported in 29 studies (10.1%), while Claude was reported in 8 studies (2.8%) and Microsoft Copilot or Bing variants in 10 studies (3.5%). In terms of chatbot modality, the literature overwhelmingly focused on text-based interactions (*n* = 263, 92.0%). Eighteen articles (6.3%) examined multimodal interactions, three (1.0%) focused exclusively on voice-based interfaces, and two (0.7%) did not report a specific chatbot modality.

### 4.4 Health topics

The included articles examined AI chatbot applications and evaluations across a wide range of health topics. Noncommunicable or chronic diseases were the most frequently studied category (*n* = 115, 40.2%), followed by general health information (*n* = 103, 36.0%). Mental health accounted for 30 articles (10.5%), and 17 articles (5.9%) focused on communicable and infectious diseases. Smaller proportions addressed maternal, sexual, and reproductive health (*n* = 11, 3.8%) and nutrition- and lifestyle-related issues (*n* = 4, 1.4%). Five articles (1.7%) addressed other specialized topics, and one (0.3%) focused on occupational health. Table 1 illustrates how AI chatbot applications and evaluations are distributed across health domains and purposes.

**Table 1:** Mapping AI Chatbot applications and evaluations across health topics and purposes.

Purpose	General health information	Noncommunicable or chronic diseases	Mental health	Communicable diseases	Other	Total
Public-facing applications general public or patients (*n* = 138)						
Seeking health information	32	46	4	6	8	96
Symptom assessment and self-care management	3	5	0	1	1	10
Emotional support	0	0	17	1	0	18
Preventive and transitional care support	2	5	0	3	6	16
Professional and organizational applications health professionals (*n* = 125), health researchers and students (*n* = 46), health institutions (*n* = 8)						
Clinical decision support	24	40	7	5	1	77
Improving administrative efficiency	11	5	1	1	0	18
Supporting patient interaction and doctor–patient communication	5	7	0	2	0	14
Enhancing continuing education	24	7	3	0	4	38
Facilitating health research	12	12	0	0	1	25
Institutional workflow support and care navigation	5	1	1	0	1	8
All articles	103	115	30	17	21	286

Articles addressing multiple purposes or stakeholder groups were counted in each applicable category. For presentation, the “Other” health-topic category combines maternal, sexual, and reproductive health (*n* = 11), other specialized health topics (*n* = 5), nutrition- and lifestyle-related health issues (*n* = 4), and occupational health (*n* = 1). Additional descriptive comparisons across stakeholder groups are provided in Supplementary Table S4.

## 5 AI Chatbot applications and evaluations for health purposes

Across the 286 included articles, 10 purposes for which AI chatbots were applied or evaluated were identified across four stakeholder groups, organized under two umbrella categories. Public-facing applications were directed toward the general public or patients and encompassed four purposes: seeking health information (*n* = 96), symptom assessment and self-care management (*n* = 10), emotional support (*n* = 18), and preventive and transitional care support (*n* = 16). Professional and organizational applications concerned practicing health professionals, health researchers and students, and health institutions. These applications encompassed six purposes: clinical decision support (*n* = 77), improving administrative efficiency (*n* = 18), supporting patient interaction and doctor–patient communication (*n* = 14), enhancing continuing education (*n* = 38), facilitating health research (*n* = 25), and institutional workflow support and care navigation (*n* = 8). Articles spanning multiple stakeholder groups or purposes were coded into all applicable categories; therefore, the counts are not mutually exclusive.

To distinguish potential or reported use from direct evidence of engagement, each article was additionally coded using four non-mutually-exclusive evidence-type flags. Output evaluation was the dominant evidence type: 184 articles (64.3%) assessed the accuracy, safety, completeness, readability, or expert concordance of chatbot outputs, typically generated from researcher-written prompts, clinical vignettes, or benchmark tasks. Self-reported perceptions and use appeared in 127 articles (44.4%), while 45 articles (15.7%) involved researcher-directed interaction through experimental, intervention, usability, or assigned-use protocols. Only seven articles (2.4%) documented naturalistic use occurring outside researcher direction. Overall, the evidence base largely concerned output potential or reported experience; evidence based on direct chatbot interaction was less common, and documented naturalistic use was especially rare.

### 5.1 Public-facing applications: general public and patients (*n* = 138)

Public-facing applications involved the general public or patients (*n* = 138) and encompassed four purposes: seeking health information (*n* = 96), symptom assessment and self-care management (*n* = 10), emotional support (*n* = 18), and preventive and transitional care support (*n* = 16).

#### 5.1.1 Seeking health information

Health information seeking was the most frequently identified purpose among public-facing applications (*n* = 96). The evidence base was dominated by potential or reported use: most articles evaluated chatbot outputs or reported users’ perceptions or self-reported use, whereas evidence on observed health-information-seeking behavior remained limited. Across these studies, three sub-uses emerged: simplifying medical information, providing condition-specific information, and offering an alternative information channel.

Output-evaluation studies assessed chatbots’ potential to simplify and translate medical content, producing more patient-friendly, readable text for materials such as pathology reports (20), dental radiology explanations (21), and cardiovascular MRI reports (22). Gains, however, were typically measured as readability, understandability, or short-term comprehension rather than clinical outcomes. Output quality also varied by language, with English generally outperforming Chinese outputs in accuracy and comprehensiveness (23, 24).

A larger body of output-evaluation studies assessed the accuracy of AI-generated information across clinical domains, commonly by comparing chatbot- and clinician-generated responses (25, 26). For common patient questions, chatbot responses received comparable or higher quality ratings and higher empathy ratings than clinician responses (25, 27), but accuracy was less consistent for specialized or context-dependent inquiries (28, 29). Users also reported lower trust in AI-chatbot medical advice as the health-related task became more complex (30). Laypeople’s evaluations of chatbot-output quality also varied by prompt informedness (31).

A third body of survey- and interview-based studies examined why the public reported turning to chatbots. Perceived usefulness, trustworthiness, and feeling respected were associated with greater engagement with and expected benefit from AI chatbots (32, 33). Respondents described chatbots as convenient, easy to use, and a rapid means of accessing health information (34, 35). Notably, individuals without a usual source of primary care were more likely to report chatbot use (36). Across studies, users trusted chatbot and search-engine content similarly (37) but reported lower likelihood of cross-checking or acting on chatbot advice (38).

#### 5.1.2 Symptom assessment and self-care management

Symptom assessment and self-care management was identified in 10 studies. Evidence consisted primarily of self-reported perceptions and use, supplemented by output evaluations and researcher-directed interactions.

##### 5.1.2.1 Symptom assessment and self-triage

Studies pointed to a gap between intended use and demonstrated diagnostic reliability. Survey evidence found high willingness to use chatbots for self-diagnosis, associated with perceived usefulness and favorable risk–benefit appraisals (39). Experimental studies, however, reported lower intended adherence to chatbot advice than to physician advice, as well as reduced trust and care-seeking intention when a physician was described as using AI assistance in diagnosis (40, 41). Both bodies of evidence rested largely on intentions rather than observed adherence or care-seeking behavior. Output evaluations also raised reliability concerns: chatbots did not consistently provide appropriate guidance in emergency scenarios (42), and showed poor agreement with specialist diagnoses in the otologic setting examined (43).

##### 5.1.2.2 Chronic disease self-care management

Studies examined chatbot support for chronic disease self-management, including symptom-related information, medication inquiries, lifestyle and dietary planning, and behavioral coaching (44, 45). The evidence consisted primarily of self-reported perceptions and use, supplemented by output evaluations and short-term researcher-directed interactions. In output evaluations, chatbot-generated self-management guidance was rated as comparable to or more comprehensive than clinician-created materials (46). Patient interviews following study-directed chatbot use described the tools as convenient and potentially supportive, while also raising concerns about limited human connection, unreliable information, limited personalization, and inadequate source transparency (44, 45). Acceptance also varied by education, gender, and prior experience with chatbots and online health-information seeking (47, 48).

#### 5.1.3 Emotional support

Eighteen studies examined AI chatbots for mental health counseling and emotional support, spanning self-reports, researcher-directed interventions, output evaluations, and studies documenting use in routine mental health care (49). Three themes emerged: the quality of AI-generated emotional support relative to human support, the motivations and barriers shaping engagement, and intervention outcomes in specific populations.

Comparative output evaluations identified broad similarities between AI-generated and human-counselor responses in professionalism, empathy, and information quality (50). Users also evaluated chatbot-based support positively, although they identified limitations in personalization and conversational naturalness (51). However, perceived authenticity declined when AI involvement was disclosed (52), and response quality varied across risk levels and prompt conditions (53). Moreover, interview and output-evaluation studies identified concerns regarding privacy, transparency, accuracy, limited assessment capability, gender-differentiated stigmatizing responses, and ethical accountability (50, 54, 55).

Another line of research examined reported motivations and barriers shaping engagement with AI chatbots for emotional support. Users described turning to AI-based support to avoid embarrassment, manage self-presentation, and obtain immediate opportunities for emotional expression (56). In contrast, doubts about helpfulness and a lack of perceived need discouraged chatbot use, whereas cost, stigma, and time were more frequently reported barriers to conventional mental health services (57).

Beyond general populations, researcher-directed interventions examined short-term outcomes among populations with distinct support needs. Findings were mixed: autistic adolescents and adults showed improved empathetic responding during social conversations (58), and pediatric and young adult patients with cancer reported lower anxiety and stress (3), whereas emotional outcomes did not differ significantly between chatbot and mindfulness conditions among older adults (59). These assigned-use studies offer preliminary evidence regarding short-term outcomes, but sustained effects in everyday settings remain largely unexamined.

#### 5.1.4 Preventive and transitional care support

Sixteen studies examined preventive and transitional *c*are support. Eleven of these included output evaluations; thus, the literature primarily assessed the quality of chatbot-generated guidance rather than whether AI-chatbot use affected preventive behavior or recovery.

##### 5.1.4.1 Preventive health care

Preventive health studies examined chatbot-generated guidance on diet (60), exercise (61), vaccination (62, 63), and cancer screening (64). Output evaluations generally found that chatbots produced tailored and broadly relevant information but sometimes omitted relevant clinical context and guideline details (62, 64). The limited participant-level evidence was mixed: a small, short-term exercise intervention reported improvements in health outcomes (61), whereas AI-generated corrections of vaccine misbeliefs sometimes backfired among participants with stronger pseudoscientific beliefs (63).

##### 5.1.4.2 Transitional care support

Transitional care studies examined AI chatbot support across postoperative, rehabilitative, and broader recovery contexts (65–70). Evaluations of AI chatbot outputs identified strengths in clarity and relevance (65, 66) and in simplifying discharge information (69). However, the outputs also showed limited clinical tailoring (67), contained omissions and hallucinated information (68, 69), and raised potential safety concerns (69). Interview evidence indicated that users valued the accessible and personalized information provided by the AI chatbot, whereas limited digital literacy, usability problems, privacy concerns, and low perceived relevance constrained engagement (70).

### 5.2 Professional and organizational applications

Among the articles on professional and organizational applications, three stakeholder groups were represented: health professionals (*n* = 125), health researchers and students (*n* = 46), and health institutions (*n* = 8). The applications and evaluations spanned six purposes. Clinical decision support (*n* = 77), improving administrative efficiency (*n* = 18), and supporting patient interaction and doctor–patient communication (*n* = 14) most directly addressed health professionals. Enhancing continuing education (*n* = 38) and facilitating health research (*n* = 25) most directly addressed health researchers and students, whereas institutional workflow support and care navigation (*n* = 8) most directly addressed health institutions. Counts are not mutually exclusive because an article could address multiple stakeholder groups or purposes. For presentation, each purpose is discussed below under the stakeholder group it most directly addressed.

#### 5.2.1 Health professionals (*n* = 125)

##### 5.2.1.1 Clinical decision support

Clinical decision support was the most frequently identified professional purpose (*n* = 77). Output evaluation predominated (*n* = 60), indicating that this literature primarily assessed chatbots’ potential to generate decision-support outputs. Chatbot outputs were evaluated for three potential functions: processing clinical information, supporting diagnostic reasoning, and assisting treatment planning.

Evaluations of clinical-information processing examined whether chatbots could transform unstructured information into structured, decision-relevant outputs. Chatbots generated structured summaries of clinical text, with selected outputs showing broad similarity to human-generated summaries (71, 72). Performance, however, was sensitive to prompt design and reproducibility. Structured prompts improved clinical adequacy but were also associated with more hallucinations, while ambiguous prompts occasionally produced inaccurate responses across repeated tests (73, 74).

Diagnostic evaluations revealed a task-dependent pattern. Chatbot outputs showed relatively favorable sensitivity or agreement on selected emergency-triage and stroke benchmarks (75, 76). However, chatbots were less accurate or specific than specialists when diagnosis required nuanced clinical interpretation, particularly in complex or atypical cases (77–79).

Treatment-planning evaluations revealed similar limitations. Across evaluations, treatment recommendations showed limited agreement with specialists (80), sometimes lacked specificity regarding dosing or treatment sequencing (81, 82), and, in some cases, were judged potentially harmful (83). Incorporating clinical guidelines into the model prompt improved agreement with expert-preferred treatments in one bipolar-depression benchmark, but some outputs still included poor or contraindicated options (5).

##### 5.2.1.2 Improving administrative efficiency

Eighteen studies examined AI chatbot applications and evaluations intended to reduce documentation and related administrative workload. The evidence base was methodologically diverse, including controlled task comparisons, pilot studies conducted in clinical settings, and cross-sectional surveys of workplace use. Controlled comparisons found that AI assistance reduced the time required to produce clinical documentation (84–86). Participants in clinical pilots also reported less time spent on documentation, less after-hours work, and lower cognitive or administrative burden (87, 88).

However, several limitations were noted. In one electronic health record integrated inbox pilot, objective time savings were not observed despite reductions in perceived task load and work exhaustion (89). Medical physicists reported improvements in efficiency more frequently than improvements in work quality (90). Documentation quality also varied across tasks and prompting conditions, including variation in factual correctness across report types (84), omissions when prompts lacked sufficient detail (91), and occasional incomplete or fabricated content (92). Clinicians additionally raised concerns about patient safety, confidentiality, and data security regarding the implementation of AI chatbots in clinical workflows (90, 93, 94).

##### 5.2.1.3 Supporting patient interaction and doctor–patient communication

Fourteen studies examined AI chatbot applications and evaluations intended to support patient interaction and doctor–patient communication. Most evidence came from output evaluations (*n* = 11) of AI-generated replies or patient-facing materials. Two principal functions were examined: responding to patient inquiries and producing patient education materials.

Evaluations of responses to patient inquiries yielded mixed findings. AI-generated replies received ratings comparable to or higher than clinician-authored replies for overall quality, communication style, or empathy (27, 95, 96). However, favorable communication ratings did not consistently indicate safer or more usable content. Higher empathy ratings sometimes coincided with lower alignment with scientific consensus and greater potential for harm (27).

Evaluations of patient-facing materials showed a similar pattern. AI chatbots could produce broadly relevant and understandable information, but outputs sometimes omitted information important to patients, exceeded accessible reading levels, or contained topic-specific inaccuracies (97–99). Human-guided revision improved the quality of AI-generated consent materials, whereas translation quality varied substantially across languages and continued to require professional review (100, 101).

Participant-level evidence was limited. In one controlled study, lay participants achieved higher understanding scores and physicians required less explanation time with GPT-4-generated interpretations than with original pathology reports (20). Effects on patient experience, patient–clinician relationships, or health outcomes in routine care remained largely unexamined.

## 5.2.2 Health researchers and students (*n* = 46)

Forty-six articles concerned health researchers or students in training. Two related, non-mutually-exclusive purposes were identified: enhancing continuing education (*n* = 38) and facilitating health research (*n* = 25). These purposes also appeared in some studies involving practicing health professionals.

### 5.2.2.1 Enhancing continuing education

Thirty-eight studies examined AI chatbot applications and evaluations in education and professional development. The evidence comprised self-reported perceptions and use, researcher-directed learning activities, and output evaluations of educational materials or assessment performance.

Perception studies indicated that students and educators valued AI chatbots for rapid access to medical information, summarizing complex material, and supporting learning efficiency (102–104). Participants also raised concerns about the accuracy and reliability of generated content, overreliance on AI, and the potential effects of AI on future employment (103, 105, 106). Organizational restrictions, geographic access barriers, and limited digital literacy further constrained adoption (107).

Researcher-directed studies used chatbots as virtual patients, role-play partners, or structured self-regulated learning tools. Participants generally described these activities as accessible and educationally useful (108–111). However, these were short-term, assigned-use activities, and their sustained learning effects remained unclear.

Output evaluations examined the quality of AI-generated instructional materials and performance on educational assessments. Across benchmark studies, AI performance exceeded that of some medical-student groups but remained below senior trainees (112, 113). Clinician raters found that many AI-generated clinical vignettes were usable after review or revision (114, 115). When used to assess clinical skills, AI systems tended to assign higher scores than human evaluators, while agreement varied by the type of skill assessed (116).

### 5.2.2.2 Facilitating health research

Facilitating health research was coded in 25 studies. The evidence consisted primarily of output evaluations and self-reported perceptions or use, focusing on two broad activities: literature synthesis and health-data analysis.

Output evaluations indicated that AI chatbots could rapidly summarize and organize medical literature and guideline information but also produced inaccurate or fabricated references and incomplete or misrepresented guideline content (117–120). Evaluations of data-analysis tasks similarly suggested that chatbots could support information extraction and pattern identification in qualitative materials and unstructured clinical notes, although reproducibility and contextual interpretation remained limited (121, 122).

Surveys and qualitative studies indicated that researchers and clinicians anticipated efficiency benefits but remained concerned about confidentiality, inaccurate outputs, overreliance, and limited ethical or regulatory guidance (123–126). The qualitative evidence further emphasized local-language support and alignment with local values and health care practices as important considerations when adopting chatbots for health-research applications (126). Overall, the literature primarily documented the potential of chatbots to support research tasks, while effects on routine workflows, productivity, and research quality remained largely unexamined.

## 5.2.3 Health institutions (*n* = 8)

### 5.2.3.1 Institutional workflow support and care navigation

Eight articles examined health institutions as implementation actors, organizational decision-makers, or contexts in which integration conditions were directly studied. Across studies, institutional involvement took the form of bounded integration into existing services. Clinician-facing applications were embedded in documentation and patient-communication workflows (87–89, 127), and patient-facing applications connected recovery and therapy support to institutionally delivered care (49, 70). Implementation remained confined to particular sites, services, or user groups, with professional oversight retained. Survey and interview evidence from health-system leaders and frontline clinicians identified staff training, adequate technical infrastructure, data-security safeguards, and clear governance as conditions shaping institutional adoption (128, 129). Overall, current evidence primarily addresses the feasibility of incorporating AI chatbot systems into existing institutional processes under supervision. Evidence concerning sustained organizational effects, scalability across services, and governance during routine use remains limited.

## 6 Discussion

This scoping review synthesized 286 empirical studies published through July 2025 to examine how AI chatbots have been applied and evaluated across 10 health-related purposes involving four stakeholder groups: the general public or patients, health professionals, health researchers and students, and health institutions. Extending prior reviews of the health chatbot literature (11, 12), the present review jointly maps stakeholder groups, application purposes, health domains, and evidence types. This integrated framework links the stakeholders studied to the purposes and health contexts in which AI chatbots were applied or evaluated, as well as to the nature of the supporting evidence. This integrated mapping shows where the literature is concentrated and where important research gaps remain.

### 6.1 Key patterns of AI Chatbot applications and evaluations across stakeholders and purposes

Four cross-cutting patterns emerged from the reviewed literature. First, applications and evaluations of AI chatbots concentrated on information-oriented and support functions. Second, the evidence remains dominated by output evaluations, with limited behavioral, clinical, and longitudinal evidence. Third, the performance of AI chatbots varied across tasks and evaluation settings. Fourth, potential benefits and risks coexisted across health applications.

#### 6.1.1 AI Chatbot applications center on information-oriented and support functions

First, across stakeholder groups, applications and evaluations of AI chatbots focused primarily on accessing, interpreting, organizing, and communicating health information. Patient-facing studies examined responses to health questions, generated educational materials, self-management guidance, and low-barrier emotional support (24, 56, 57, 130). Professional applications included clinical documentation, patient-facing communication, and guideline-informed decision support, with generated outputs generally positioned for clinician review (5, 84, 89). Early institutional implementations similarly embedded LLM-enabled tools within existing documentation and patient-communication workflows while retaining professional oversight (87, 89, 127). Taken together, current evidence positions AI chatbots primarily as a supplementary layer for information processing and communication within existing services, with human oversight remaining central to their application.

#### 6.1.2 Evidence remains dominated by output evaluation, with limited behavioral, clinical, and longitudinal evidence

Second, the evidence base is largely concentrated at the level of chatbot outputs and self-reported perceptions or use. Direct evidence of sustained engagement and downstream effects remains limited. Output evaluations were identified in 184 studies (64.3%), and 127 studies (44.4%) examined self-reported perceptions or use. Much of the literature therefore assessed the accuracy, completeness, or readability of AI-generated outputs, or examined participants’ perceptions, intentions, and self-reported use. These forms of evidence characterized generated content and reported experiences but did not establish behavioral, clinical, or longitudinal effects. Output-evaluation findings were also contingent on researcher-selected prompts and evaluation criteria, limiting their comparability across studies and generalizability to routine interactions. Direct engagement was less common: 45 studies (15.7%) involved researcher-directed interaction, and only seven (2.4%) documented naturalistic use. Studies involving direct interaction generally assessed perceptions, intentions, or short-term task performance. The distribution of evidence across health contexts was similarly uneven. General health information was addressed in 36.0% of studies and noncommunicable or chronic diseases in 40.2%, while higher-acuity, time-sensitive, and specialist-dependent applications were less frequently examined. Taken together, current evidence primarily concerns chatbot outputs and self-reported perceptions or use. Evidence concerning sustained use and behavioral or clinical outcomes over time remains limited, particularly in higher-stakes contexts.

#### 6.1.3 AI Chatbot performance varies across tasks and evaluation settings

Third, the performance of AI chatbots in health contexts appears to vary by task demands and evaluation setting. Across the reviewed studies, AI chatbots tended to receive favorable evaluations on common, structured, and standardized tasks, although performance varied by task, specialty area, model, and human comparator (25, 112, 113). By contrast, performance was less consistent when tasks required specialized clinical judgment or atypical-case interpretation (131–133). This pattern highlights the importance of assessing AI chatbot performance in relation to specific tasks, populations, and clinical contexts.

#### 6.1.4 Potential benefits and risks coexist across health applications

Across contexts, reported benefits and risks were often linked to the same functional features of AI chatbots. Fluent and tailored communication was associated with favorable ratings of empathy, satisfaction, and preference, but these ratings did not necessarily indicate clinical accuracy or safety (25, 26). In some evaluations, greater perceived empathy coexisted with weaker alignment with scientific consensus and more potentially harmful content (27). Similarly, low-friction and multilingual communication could extend access to health information, while uneven output quality across languages and differences in digital literacy, usability, privacy concerns, and perceived relevance may limit who could benefit (23, 24, 70, 101). Workflow support similarly offered potential efficiency gains, although generated documentation still required professional review and adaptation to existing workflows (84, 88). Overall, potential benefits in communication, access, and efficiency remained conditional on accuracy, equity, and professional oversight.

### 6.2 Research gaps and future directions

#### 6.2.1 Advancing research on institutional integration, governance, and real-world deployment

First, empirical research on the institutional integration and real-world deployment of AI chatbots remains limited. The institution-focused studies identified in this review examined bounded integration into existing clinical and patient-facing workflows (49, 87, 89) and organizational readiness for implementation (128), and health-system leaders’ perspectives on broader AI and LLM integration (129). Overall, the reviewed studies captured early integration and implementation conditions, although the evidence remains concentrated in single-site or narrowly scoped evaluations. Future research should therefore examine real-world deployment across the full institutional lifecycle, including how health institutions select and govern AI chatbots, how these systems are integrated into clinical workflows, and how their sustained use, clinical outcomes, equity, and performance are monitored over time.

#### 6.2.2 Advancing behavioral, clinical, and longitudinal evidence

Second, future research should give greater attention to behavioral and clinical outcomes over time. Despite the rapid expansion of the field, much of the evidence remains concentrated on output evaluations, self-reported perceptions or use, and short-term interaction outcomes. Although these forms of evidence are useful for characterizing potential capability and early feasibility, they offer limited insight into how chatbot engagement influences patient behavior, clinical outcomes, care quality, or professional workflows over time. Prior reviews have similarly identified limited long-term and clinical-efficacy evidence in related health-chatbot research (134). Future research should use prospective and longitudinal designs to examine downstream outcomes, including treatment adherence, help-seeking behavior, clinical decision quality, and clinician workload.

#### 6.2.3 Extending evidence across populations, languages, and modalities

Third, future research should examine how the accessibility and performance of AI chatbots vary across populations, languages, settings, and interaction modalities. The human-participant evidence in this review is concentrated in North America, Western Europe, East Asia, and the Middle East with comparatively little research conducted in low- and middle-income countries. The evidence base is also dominated by text-based interaction, while voice-based, image-based, and multimodal applications were rarely examined. The patterns identified in this review therefore primarily characterize text-based chatbot applications. Emerging multimodal LLMs and AI agents may support more complex forms of interaction and task completion while introducing new challenges related to user oversight, data governance, and accountability. Future studies should include more diverse populations and compare text, voice, and multimodal interfaces across languages and settings, with attention to accuracy, accessibility, comprehension, user experience, and the distribution of potential benefits and risks.

### 6.3 Implications for digital public health and governance

The findings have three implications for digital public health and governance. First, AI chatbots represent an additional source of health information outside formal health care settings (36, 57). Because such use may occur outside formal services, users may encounter content without clinical validation or ongoing health-sector oversight (135). Governance needs to clarify who is responsible for validating health-related outputs, how harmful information can be reported and corrected, and where liability lies when unsafe guidance causes harm (135, 136).

Second, making a chatbot available does not ensure equal access to usable health information. Output evaluations found that health information often exceeded recommended reading levels and that quality varied across languages (23, 24, 101, 137). These findings point to the need for language and readability testing and for user education about system limitations, information verification, and circumstances requiring professional care. Moreover, risks associated with AI chatbots, such as hallucinated outputs and limited model transparency, may vary across stakeholder groups, given differences in how chatbot outputs are accessed, interpreted, and acted upon. Future research should examine these concerns more specifically across public and patient, professional, research and educational, and institutional contexts.

Third, only eight articles directly involved health institutions, and implementation remained early and narrowly scoped. Reported gaps in health-system governance and postdeployment monitoring underscore the need for clearer institutional procedures (129). Existing governance and regulatory frameworks address some of these concerns. For example, the World Health Organization’s guidance for large multimodal models in health emphasizes transparency, explainability, accountability, and post-release evaluation (135). The U. S. Food and Drug Administration’s draft guidance addresses lifecycle risk management for AI-enabled medical device software (138), while the European Union Artificial Intelligence Act establishes requirements for risk management, transparency, human oversight, and postmarket monitoring for qualifying high-risk AI systems (139). However, little empirical research has examined how these principles and requirements are implemented in practice, particularly at the organizational level. Further research is needed to evaluate their application in routine health-system settings.

### 6.4 Limitations

This review has several limitations. First, the search queried abstract-level keywords and was restricted to records available in full text, which may have missed studies where chatbot use appears only in the body of an article and introduced retrieval bias tied to institutional access. Although the search covered nine interdisciplinary databases, additional specialized databases were not included. Future reviews could broaden database coverage and search full text where database access permits.

Second, the review was restricted to peer-reviewed journal articles published in English; gray literature, conference proceedings, and dissertations were not included. These restrictions may have excluded chatbot applications developed in different linguistic and cultural contexts and underrepresented null or unfavorable findings. Future reviews could broaden the inclusion criteria to incorporate selected gray literature and non-English-language studies.

Third, the review was descriptive and mapping-oriented rather than meta-analytic; accordingly, it identifies patterns in the evidence base but does not synthesize pooled effect estimates or quantify overall effectiveness. We also did not formally assess the methodological quality or risk of bias of the included studies, so findings concerning accuracy, safety, effectiveness, and risk should be interpreted cautiously.

Fourth, given the rapid pace of development in this field, the search was closed in July 2025, and additional evidence has likely emerged since that time. Moreover, because the current evidence base is dominated by studies of ChatGPT, the findings may partly reflect the capabilities and use patterns of this particular platform and may not generalize to other emerging foundation models. Future studies may extend this review to capture the rapidly evolving evidence base on AI chatbots in health care. Relatedly, future research could apply a more nuanced stakeholder coding scheme to distinguish sub-groups such as caregivers within the general public or patients.

Finally, it should be noted that this review examined empirical studies rather than real-world implementations of AI chatbots. As such, the findings reflect patterns of scholarly attention rather than the actual prevalence of generative AI applications in practice.

## 7 Conclusion

This scoping review mapped 286 empirical studies of AI chatbot applications and evaluations across 10 health-related purposes involving four stakeholder groups: the general public or patients, health professionals, health researchers and students, and health institutions. The evidence base is expanding rapidly yet remains concentrated on text-based interaction, chatbot outputs, and self-reported perceptions or use. Sustained engagement, behavioral and clinical outcomes, and routine organizational integration remain underexamined. By linking stakeholders, purposes, and evidence types, the review provides a structured account of where AI chatbots are being studied in health care and identifies gaps in the evidence base.
