# Supplementary material for: Mapping applications and evaluations of LLM-enabled AI chatbots for health purposes: a scoping review
Source: Front Public Health. 2026 Sep 22;14:1916780. doi: 10.3389/fpubh.2026.1916780 (PMC13639851; doi:10.3389/fpubh.2026.1916780)
Supplement: Supplementary file 1 [file Table_1.docx]

**Supplementary Table S1. Coding Results of the 286 Included Articles**

**Coding Category**

| **Human involvement** | 1 = None; 2 = Evaluator or comparator only; 3 = Participant |
| --- | --- |
| **Method(s)** | 1=Interview; 2=Focus group; 3=Text analysis; 4=Delphi study; 5=Experiment; 6=Survey; 7=Content analysis; 8=Network analysis. |
| **Evidence type(s)** | 1=Output evaluation; 2=Self-reported perceptions and use; 3=Researcher-directed interaction; 4=Documented naturalistic use. |
| **LLM type** | 1=General-purpose LLM; 2=Custom LLM application; 3=Unspecified. |
| **Chatbot/model(s)** | 1=ChatGPT; 2=Google Bard; 3=Google Gemini; 4=Claude; 5=Microsoft Copilot; 6=Microsoft Bing; 7=Other (specific name); 8=Unspecified. |
| **Chatbot modality** | 1=Text-based; 2=Multimodal; 3=Voice-based; 4=Unspecified. |
| **Health topic** | 1=Communicable/infectious diseases; 2=Noncommunicable/chronic diseases; 3=Mental health; 4=Maternal, sexual, and reproductive health; 5=Nutrition/lifestyle; 6=General health information; 7=Occupational health; 8=Other. |
| **Stakeholder(s)** | 1=Public/patients; 2=Health professionals; 3=Health researchers/students; 4=Health institutions. |
| **Purpose(s)** | 1=Seeking health information; 2=Symptom assessment/self-care management; 3=Emotional support; 4=Preventive/transitional care; 5=Clinical decision support; 6=Administrative efficiency; 7=Patient interaction/doctor-patient communication; 8=Continuing education; 9=Health research; 10=Institutional workflow/care navigation. |

*Note. Multiple codes may apply; multiple codes are separated by semicolons. NR = not reported; — = not applicable*

| **ID and author (year)** | **Human involvement (participant n)** | **Participant country** | **Method(s)** | **Evidence type(s)** | **LLM type** | **Chatbot/model(s)** | **Chatbot modality** | **Health topic** | **Stakeholder(s)** | **Purpose(s)** |
| --- | --- | --- | --- | --- | --- | --- | --- | --- | --- | --- |
| 1. Abate et al. (2025) | 2 | — | 3; 7 | 1 | 1 | 1; 3 | 1 | 1 | 2 | 5 |
| 2. Abdelgadir et al. (2025) | 2 | — | 3; 7 | 1 | 1 | 1 | 1 | 2 | 1 | 1 |
| 3. Abouammoh et al. (2025) | 3 (n = 12) | Saudi Arabia | 2 | 2 | 1 | 1 | 1 | 6 | 2; 3 | 8 |
| 4. Abu Hammour et al. (2023) | 3 (n = 359) | Jordan | 6 | 2 | 1 | 1 | 1 | 6 | 2 | 8 |
| 5. Abu Hammour et al. (2024) | 3 (n = 244) | Jordan | 6 | 2 | 1 | 1 | 1 | 6 | 3 | 8 |
| 6. Ajlouni et al. (2023) | 3 (n = 210) | Jordan | 6 | 2 | 1 | 1 | 1 | 3 | 3 | 8 |
| 7. Aközlü et al. (2024) | 1 | — | 7 | 1 | 1 | 1 | 1 | 2 | 2 | 5 |
| 8. Al-Anezi (2024) | 3 (n = 29) | Saudi Arabia | 1; 5 | 2; 3 | 1 | 1 | 1 | 2 | 1 | 2 |
| 9. Al Shboul et al. (2024) | 3 (n = 16) | Multiple:Jordan, Kuwait, United Arab Emirates | 1 | 2 | 1 | 1 | 1 | 6 | 1 | 1 |
| 10. Al Tibi et al. (2024) | 2 | — | 7 | 1 | 1 | 1 | 1 | 2 | 2 | 5 |
| 11. Alam et al. (2025) | 2 | — | 7 | 1 | 1 | 1 | 2 | 2 | 2 | 5 |
| 12. Alanezi (2024) | 3 (n = 24) | Saudi Arabia | 1; 5 | 2; 3 | 1 | 1 | 1 | 3 | 1 | 3 |
| 13. Alanezi (2024) | 3 (n = 163) | Saudi Arabia | 1; 5; 6 | 2; 3 | 1 | 1 | 1 | 6 | 1 | 1 |
| 14. Alanzi (2023) | 3 (n = 54) | Saudi Arabia | 2 | 2 | 1 | 1 | 1 | 6 | 2 | 5 |
| 15. Alanzi et al. (2023) | 3 (n = 507) | Saudi Arabia | 1; 6 | 2 | 1 | 1 | 1 | 2 | 1 | 1 |
| 16. Albogami et al. (2024) | 2 | — | 7 | 1 | 1 | 1 | 1 | 2 | 2 | 5 |
| 17. Ali et al. (2024) | 1 | — | 3; 7 | 1 | 1 | 1 | 1 | 6 | 1 | 1 |
| 18. Aliyeva et al. (2024) | 1 | — | 7 | 1 | 1 | 1 | 1 | 2 | 1 | 4 |
| 19. Alkhamees (2024) | 2 | — | 7 | 1 | 1 | 1 | 1 | 2 | 1 | 1 |
| 20. Allen et al. (2025) | 1 | — | 7 | 1 | 1 | 1 | 1 | 2 | 2 | 5 |
| 21. Alruwaili et al. (2025) | 3 (n = 33) | Saudi Arabia | 1; 2 | 2 | 1 | 8 | 1 | 8 | 2; 4 | 5; 10 |
| 22. Arbanas et al. (2025) | 3 (n = 89) | Multiple: Bosnia and Herzegovina; Croatia | 6; 7 | 2; 3 | 1 | 1; 7 (Pomozi) | 1 | 3 | 1 | 1 |
| 23. Argan et al. (2024) | 3 (n = 1135) | Turkey | 6 | 2 | 1 | 1 | 1 | 6 | 1 | 1 |
| 24. Armbruster et al. (2024) | 3 (n = 64) | Germany | 6; 7 | 1; 2 | 1 | 1 | 1 | 6 | 1 | 1 |
| 25. Armitage (2025) | 3 (n = 107) | UK | 6 | 2 | 1 | 8 | 1 | 6 | 2 | 6 |
| 26. Armstrong et al. (2024) | 3 (n = 8) | USA | 6; 7 | 1; 2 | 1 | 1 | 1 | 2 | 1; 2 | 1; 7 |
| 27. Arvidsson et al. (2024) | 2 | — | 7 | 1 | 1 | 1 | 1 | 6 | 2 | 5 |
| 28. Aster et al. (2025) | 3 (n = 28) | Germany | 5; 7 | 1; 2; 3 | 1 | 1 | 1 | 2 | 3 | 8 |
| 29. Ataguba et al. (2025) | 3 (n = 17) | Canada | 1; 6; 7; 8 | 1; 2; 3 | 1 | 1 | 1 | 5 | 1 | 4 |
| 30. Ayers et al. (2023) | 1 | — | 7 | 1 | 1 | 1 | 1 | 6 | 1 | 1 |
| 31. Ayo-Ajibola et al. (2024) | 3 (n = 2406) | USA | 6 | 2 | 1 | 1 | 1 | 6 | 1 | 1 |
| 32. Ayo-Ajibola et al. (2025) | 3 (n = 2406) | USA | 6 | 2 | 1 | 1 | 1 | 6 | 1 | 1 |
| 33. Ayoub et al. (2023) | 2 | — | 7 | 1 | 1 | 1 | 1 | 2 | 2 | 5 |
| 34. Ayre et al. (2024) | 1 | — | 3; 7 | 1 | 1 | 1 | 1 | 6 | 1 | 1 |
| 35. Başaran et al. (2024) | 3 (n = 93) | Turkey | 5 | 2 | 1 | 1 | 1 | 4 | 3 | 8 |
| 36. Beckmann et al. (2025) | 3 (n = 1323) | Germany | 5; 6 | 2 | 1 | 1 | 1 | 1 | 1 | 1 |
| 37. Benfatah et al. (2024) | 3 (n = 12) | Morocco | 5; 7 | 2; 3 | 1 | 1 | 1 | 6 | 3 | 8 |
| 38. Berend et al. (2025) | 2 | — | 7 | 1 | 1 | 1 | 1 | 2 | 2 | 5 |
| 39. Bernstein et al. (2023) | 2 | — | 7 | 1 | 1 | 1 | 1 | 2 | 1 | 1 |
| 40. Bhattacharyya et al. (2023) | 2 | — | 7 | 1 | 1 | 1 | 1 | 6 | 3 | 9 |
| 41. Blease et al. (2024) | 3 (n = 1006) | UK | 6 | 2 | 1 | 1; 2; 6 | 1 | 6 | 2 | 6 |
| 42. Bohn et al. (2024) | 2 | — | 7 | 1 | 1 | 1; 7 (BLOOM) | 1 | 2 | 2 | 5 |
| 43. Boonrit et al. (2024) | 3 (n = 5) | Thailand | 1; 7 | 1; 2 | 1 | 1 | 1 | 6 | 2 | 5 |
| 44. Brigo et al. (2025) | 2 | — | 7 | 1 | 1 | 1 | 1 | 2 | 2 | 5 |
| 45. Brigo et al. (2025) | 2 | — | 7 | 1 | 1 | 1 | 1 | 2 | 2 | 5 |
| 46. Buholayka et al. (2023) | 1 | — | 7 | 1 | 1 | 1 | 1 | 2 | 2 | 9 |
| 47. Bundy et al. (2024) | 3 (n = 12) | USA | 1 | 2 | 2 | 5 | 3 | 6 | 2; 4 | 6; 10 |
| 48. Burford et al. (2024) | 1 | — | 3; 7 | 1 | 1 | 1 | 1 | 6 | 3 | 9 |
| 49. Bushuven et al. (2023) | 1 | — | 7 | 1 | 1 | 1 | 1 | 8 | 1 | 2 |
| 50. Buz (2024) | 3 (n = 10) | Turkey | 5; 7 | 1; 3 | 1 | 1 | 1 | 2 | 3 | 8 |
| 51. Campellone et al. (2025) | 3 (n = 160) | USA | 5; 7 | 1; 2; 3 | 2 | 7 (Gen-W-MA, text-embedding-ada-002) | 1 | 3 | 1 | 3 |
| 52. Chandler et al. (2024) | 1 | — | 7 | 1 | 1 | 1 | 1 | 1 | 1 | 1 |
| 53. Chandler et al. (2025) | 1 | — | 7 | 1 | 1 | 1 | 1 | 6 | 1 | 1 |
| 54. Chang et al. (2024) | 2 | — | 3; 7 | 1 | 1 | 1 | 1 | 2 | 1; 2 | 1; 8 |
| 55. Chen et al. (2024) | 3 (n = 659) | China | 6 | 2 | 1 | 1 | 1 | 6 | 1; 3 | 1; 8; 9 |
| 56. Chen et al. (2025) | 3 (n = 52) | China | 2; 5 | 2; 3 | 1 | 1 | 2 | 4 | 3 | 8 |
| 57. Chen et al. (2025) | 3 (n = 1762) | USA | 5 | 2 | 1 | 8 | 1 | 6 | 1 | 2 |
| 58. Cheng et al. (2024) | 3 (n = 120) | China | 5 | 2; 3 | 1 | 1 | 1 | 4 | 1 | 1 |
| 59. Choi et al. (2024) | 2 | — | 7 | 1 | 1 | 1 | 1 | 2 | 1; 2 | 1; 7 |
| 60. Chou et al. (2025) | 3 (n = 31) | Taiwan | 3; 6; 7 | 1; 2; 3 | 1 | 1 | 1 | 2 | 1 | 1 |
| 61. Choudhury et al. (2024) | 3 (n = 607) | USA | 6 | 2 | 1 | 1 | 1 | 6 | 1 | 1 |
| 62. Choudhury et al. (2025) | 3 (n = 556) | Multiple: India; United Kingdom; United States | 6 | 2 | 1 | 7 (DeepSeek) | 1 | 6 | 1 | 1 |
| 63. Christofi et al. (2025) | 3 (n = 21) | Cyprus | 7 | 1; 3 | 1 | 1 | 1 | 4 | 1 | 1 |
| 64. Colakca et al. (2024) | 2 | — | 7 | 1 | 1 | 1 | 1 | 6 | 2 | 5 |
| 65. Cosma et al. (2025) | 2 | — | 3; 7 | 1 | 1 | 1 | 1 | 1 | 2 | 7 |
| 66. Creswell Báez et al. (2025) | 3 (n = 29) | USA | 1 | 2; 3 | 1 | 1; 2 | 1 | 3 | 2 | 5 |
| 67. Criss et al. (2024) | 1 | — | 7 | 1 | 1 | 1 | 1 | 1 | 1 | 1; 3 |
| 68. Dal et al. (2024) | 1 | — | 7 | 1 | 1 | 1 | 1 | 2 | 2 | 5 |
| 69. de Ruiter et al. (2025) | 3 (n = 106) | Netherlands | 6 | 2 | 1 | 1 | 1 | 6 | 2 | 5; 6 |
| 70. Dolev-Cohen et al. (2025) | 3 (n = 153) | Israel | 6; 7 | 1; 3 | 1 | 1 | 1 | 4 | 1 | 4 |
| 71. Dos Santos et al. (2024) | 1 | — | 7 | 1 | 1 | 1 | 1 | 2 | 2 | 5 |
| 72. Du et al. (2025) | 2 | — | 7 | 1 | 1 | 1; 3; 4; 7 (DeepSeek) | 1 | 2 | 1 | 2 |
| 73. Dubinski et al. (2024) | 3 (n = 2) | Germany | 5; 7 | 1; 3 | 1 | 1 | 1 | 2 | 2 | 6 |
| 74. Dzuali et al. (2024) | 3 (n = 3) | USA | 3; 6; 7 | 1; 2 | 1 | 1 | 1 | 6 | 1; 2 | 1; 7 |
| 75. Ebrahimi et al. (2025) | 2 | — | 7 | 1 | 1 | 1; 3 | 1 | 2 | 1 | 1 |
| 76. El Haj et al. (2024) | 1 | — | 7 | 1 | 1 | 1 | 1 | 2 | 2 | 5 |
| 77. Elpasiony et al. (2025) | 3 (n = 1001) | Egypt | 6 | 2 | 1 | 1 | 1 | 6 | 3 | 9 |
| 78. Erdat et al. (2025) | 3 (n = 147) | Turkey | 6 | 2 | 1 | 1; 3; 4; 6; 7 (Meta Llama) | 1 | 2 | 2 | 5; 9 |
| 79. Evans et al. (2025) | 3 (n = 305) | Australia | 1; 5; 6 | 2; 3 | 2 | 7 (Lyrebird Health AI scribe) | 3 | 6 | 2; 4 | 6; 7; 10 |
| 80. Fukuzawa et al. (2024) | 1 | — | 7 | 1 | 1 | 1 | 1 | 1 | 2 | 5 |
| 81. Galido et al. (2023) | 1 | — | 7 | 1 | 1 | 1 | 1 | 3 | 2 | 5 |
| 82. Garcia et al. (2024) | 3 (n = 162) | USA | 5 | 2; 4 | 2 | 1; 7 (Epic Generated Draft Replies) | 1 | 6 | 2; 4 | 6; 10 |
| 83. Gencer et al. (2025) | 2 | — | 7 | 1 | 1 | 1 | 1 | 4 | 1 | 4 |
| 84. Genes et al. (2024) | 2 | — | 7 | 1 | 1 | 1 | 1 | 2 | 2 | 5 |
| 85. Gill et al. (2024) | 2 | — | 3; 7 | 1 | 1 | 1 | 1 | 6 | 3 | 9 |
| 86. Giray (2025) | 3 (n = 6) | Unspecified | 7 | 2; 4 | 1 | 1 | 1 | 3 | 1 | 3 |
| 87. Girton et al. (2024) | 2 | — | 3; 7 | 1 | 1 | 1 | 1 | 6 | 1 | 1 |
| 88. Goh et al. (2024) | 3 (n = 50) | USA | 5; 7 | 1; 3 | 1 | 1 | 1 | 6 | 2 | 5 |
| 89. Gondode et al. (2024) | 2 | — | 3; 7 | 1 | 1 | 1 | 1 | 2 | 1 | 1 |
| 90. Guleria et al. (2024) | 3 (n = 32) | USA | 6 | 2 | 3 | 8 | 1 | 6 | 4 | 10 |
| 91. Gürses et al. (2025) | 2 | — | 7 | 1 | 1 | 1; 2; 3 | 1 | 2 | 2 | 5 |
| 92. Habicht et al. (2025) | 3 (n = 244) | UK | 6 | 2; 3; 4 | 2 | 7 (Limbic Care) | 1 | 3 | 1; 4 | 3; 10 |
| 93. Hamed et al. (2023) | 1 | — | 7 | 1 | 1 | 1 | 1 | 2 | 2 | 9 |
| 94. Hanai et al. (2024) | 1 | — | 3; 7 | 1 | 1 | 1 | 1 | 4 | 1 | 1 |
| 95. Harari et al. (2025) | 3 (n = 54) | USA | 5; 7 | 1; 2; 3 | 1 | 1 | 1 | 2 | 1 | 1 |
| 96. Hasei et al. (2025) | 3 (n = 5) | Japan | 5 | 3 | 1 | 1 | 1 | 3 | 1 | 3 |
| 97. He et al. (2024) | 2 | — | 3; 7 | 1 | 1 | 1; 7 (MedAlpaca, Meta Llama, ORCA-mini) | 1 | 6 | 1 | 1 |
| 98. Hernandez et al. (2023) | 2 | — | 7 | 1 | 1 | 1 | 1 | 2 | 1 | 1 |
| 99. Hipgrave et al. (2025) | 3 (n = 23) | Australian 19 (82.6%), Norwegian 1 (4.3%), Irish 1 (4.3%), Uzbekistani 1 (4.3%), Filipino 1 (4.3%) | 1; 6 | 2 | 1 | 1 | 1 | 3 | 1 | 3 |
| 100. Hoj et al. (2023) | 2 | — | 7 | 1 | 1 | 1 | 1 | 2 | 1 | 1 |
| 101. Hose et al. (2025) | 3 (n = 30) | USA | 1; 6 | 2; 3 | 1 | 1 | 1 | 2 | 1 | 2 |
| 102. Huang et al. (2024) | 3 (n = 6) | China | 1 | 2; 3 | 1 | 1 | 1 | 6 | 3 | 8 |
| 103. Huang et al. (2024) | 3 (n = 156) | USA | 5 | 2 | 1 | 1 | 1 | 6 | 1 | 4 |
| 104. Huang et al. (2025) | 1 | — | 3; 7 | 1 | 1 | 1 | 1 | 3 | 1 | 3 |
| 105. Huo et al. (2024) | 1 | — | 7 | 1 | 1 | 1; 4 | 1 | 2 | 1; 2 | 1; 5 |
| 106. Jain et al. (2024) | 3 (n = 140) | India | 5 | 2 | 1 | 1 | 1 | 3 | 1 | 3 |
| 107. Jairoun et al. (2024) | 3 (n = 25) | United Arab Emirates | 1 | 2 | 1 | 1 | 1 | 2 | 2 | 5 |
| 108. Ji et al. (2024) | 3 (n = 90) | China | 5; 7 | 1; 2; 3 | 1 | 1 | 1 | 6 | 2 | 8 |
| 109. Ju et al. (2025) | 3 (n = 40) | South Korea | 1; 5; 7 | 1; 2; 3 | 2 | 7 (SmartENR AI) | 1 | 6 | 2 | 6 |
| 110. Juhi et al. (2023) | 2 | — | 3; 7 | 1 | 1 | 1 | 1 | 6 | 1 | 1 |
| 111. Kaneda et al. (2023) | 1 | — | 7 | 1 | 1 | 1 | 1 | 2 | 2 | 5; 6 |
| 112. Kang et al. (2025) | 3 (n = 20) | South Korea | 1; 6 | 1; 2; 3 | 2 | 7 (HoMemeTown Dr. CareSam) | 1 | 3 | 1 | 3 |
| 113. Karaçay et al. (2025) | 2 | — | 7 | 1 | 1 | 1 | 2 | 2 | 2 | 5 |
| 114. Kharko et al. (2024) | 1 | — | 3; 7 | 1 | 1 | 1 | 1 | 6 | 2 | 6 |
| 115. Kim et al. (2023) | 3 (n = 24) | South Korea | 5 | 2 | 1 | 1 | 1 | 2 | 1 | 2 |
| 116. Kim et al. (2025) | 3 (n = 5) | USA | 3; 6; 7 | 2 | 1 | 1 | 1 | 2 | 2 | 7 |
| 117. Kim et al. (2025) | 2 | — | 7 | 1 | 1 | 4 | 1 | 6 | 1 | 1 |
| 118. Kishimoto et al. (2025) | 3 (n = 98) | China | 3; 5 | 3 | 1 | 1 | 1 | 3 | 1 | 3 |
| 119. Koegel et al. (2025) | 3 (n = 30) | USA | 5; 7 | 3 | 1 | 1 | 2 | 3 | 1 | 3 |
| 120. Koh et al. (2024) | 1 | — | 7 | 1 | 1 | 1 | 1 | 1 | 1 | 1 |
| 121. Koh et al. (2025) | 2 | — | 7 | 1 | 1 | 1 | 1 | 1 | 1 | 4 |
| 122. Koh et al. (2025) | 1 | — | 7 | 1 | 1 | 1 | 1 | 1 | 2 | 6 |
| 123. Kuo et al. (2024) | 2 | — | 7 | 1 | 1 | 1 | 1 | 6 | 1; 2 | 1; 7 |
| 124. Lang et al. (2024) | 2 | — | 7 | 1 | 1 | 1; 2 | 1 | 2 | 1; 2 | 1; 7 |
| 125. Lang et al. (2025) | 3 (n = 10) | Multiple: France; Germany; Spain; Switzerland; Turkey; United Kingdom | 6; 7 | 1; 2 | 1 | 1 | 1 | 2 | 1; 2 | 1; 7 |
| 126. Latt et al. (2025) | 2 | — | 7 | 1 | 2 | 7 (Alice, Azure chatbot) | 1 | 4 | 1 | 1 |
| 127. Lauderdale et al. (2025) | 1 | — | 7 | 1 | 1 | 1; 3 | 1 | 3 | 1 | 3 |
| 128. Lee et al. (2024) | 3 (n = 565) | USA | 6 | 2 | 1 | 1 | 1 | 2 | 1 | 1 |
| 129. Levin et al. (2025) | 3 (n = 74) | Israel | 3; 5; 7 | 1; 3 | 1 | 1; 4 | 1 | 6 | 2 | 5 |
| 130. Levit et al. (2025) | 3 (n = 33) | Israel | 1; 7 | 1; 2 | 1 | 1; 3 | 1 | 2 | 3 | 9 |
| 131. Levkovich et al. (2023) | 1 | — | 7 | 1 | 1 | 1 | 1 | 3 | 2 | 5 |
| 132. Levkovich et al. (2024) | 2 | — | 7 | 1 | 1 | 1; 3; 4 | 1 | 3 | 2 | 5 |
| 133. Leypold et al. (2024) | 1 | — | 7 | 1 | 1 | 1 | 1 | 2 | 2 | 5 |
| 134. Li et al. (2024) | 3 (n = 228) | China | 6 | 2 | 1 | 1 | 1 | 2 | 2 | 5 |
| 135. Li et al. (2024) | 3 (n = 20) | USA | 1; 7 | 1; 2 | 1 | 1 | 1 | 2 | 3 | 9 |
| 136. Li et al. (2024) | 3 (n = 535) | China | 5 | 2 | 1 | 7 (ERNIE Bot) | 1 | 1 | 1 | 2 |
| 137. Li et al. (2024) | 3 (n = 306) | China | 5 | 2 | 1 | 1 | 1 | 3 | 1 | 1; 3 |
| 138. Lian et al. (2024) | 2 | — | 7 | 1 | 1 | 1 | 1 | 2 | 2 | 5 |
| 139. Liao et al. (2023) | 1 | — | 3; 7 | 1 | 1 | 1; 7 (Perplexity AI) | 1 | 6 | 3 | 9 |
| 140. Lim et al. (2023) | 3 (n = 149) | USA | 3; 5; 7 | 1; 2 | 1 | 7 (BLOOM) | 1 | 4 | 1 | 1 |
| 141. Lim et al. (2023) | 1 | — | 7 | 1 | 1 | 1; 2; 7 (Blue Willow, DALL-E, Midjourney) | 2 | 8 | 3 | 8 |
| 142. Lin et al. (2025) | 1 | — | 7 | 1 | 1 | 1 | 1 | 2 | 2 | 5 |
| 143. Lin et al. (2025) | 3 (n = 270) | China | 6 | 2 | 1 | 1 | 1 | 6 | 2 | 8 |
| 144. Linardon et al. (2025) | 3 (n = 271) | Multiple: Australia; Canada; Germany; Greece; Ireland; Italy; New Zealand; Scotland; Sweden; United Kingdom; United States; other countries not individually reported | 6 | 2 | 1 | 1; 2; 7 (Wysa) | 1 | 3 | 1; 2 | 1; 5 |
| 145. Lindbäck et al. (2025) | 3 (n = 15) | Sweden | 2 | 2 | 1 | 8 | 1 | 6 | 3 | 8 |
| 146. Liu et al. (2023) | 2 | — | 7 | 1 | 1 | 1 | 1 | 2 | 2 | 5 |
| 147. Liu et al. (2023) | 2 | — | 7 | 1 | 1 | 1 | 1 | 6 | 2 | 5 |
| 148. Liu et al. (2024) | 2 | — | 7 | 1 | 1 | 1 | 1 | 6 | 2 | 5 |
| 149. Liu et al. (2025) | 2 | — | 7 | 1 | 1 | 1 | 1 | 2 | 1 | 1 |
| 150. Liu et al. (2025) | 3 (n = 401) | China | 6 | 2 | 3 | 8 | 1 | 6 | 1 | 1 |
| 151. Liu et al. (2025) | 1 | — | 3; 7 | 1 | 1 | 1 | 1 | 3 | 1 | 3 |
| 152. Liu et al. (2025) | 3 (n = 13) | China | 1 | 2 | 1 | 8 | 1 | 6 | 2 | 8 |
| 153. Lockwood et al. (2025) | 3 (n = 249) | USA | 6; 7 | 1; 2 | 1 | 1 | 1 | 3 | 2 | 6 |
| 154. Logan et al. (2025) | 2 | — | 7 | 1 | 1 | 1; 3 | 2 | 2 | 1 | 4 |
| 155. Lu et al. (2025) | 3 (n = 5) | China | 5; 7 | 1; 3 | 1 | 1 | 1 | 2 | 2 | 6 |
| 156. Lu (2025) | 3 (n = 1435) | USA | 5 | 2 | 1 | 1 | 1 | 1 | 1 | 4 |
| 157. Luo et al. (2025) | 3 (n = 210) | Multiple: countries not individually reported | 6 | 2 | 1 | 1; 3; 4 | 1 | 6 | 2; 3 | 9 |
| 158. Lv et al. (2024) | 2 | — | 7 | 1 | 1 | 1; 2 | 1 | 2 | 1 | 1 |
| 159. Ma et al. (2024) | 3 (n = 21) | China | 1 | 2 | 1 | 8 | 2 | 3 | 2 | 5 |
| 160. Ma et al. (2025) | 3 (n = 24) | China | 1 | 2 | 1 | 1; 6 | 1 | 6 | 3 | 8 |
| 161. Maida et al. (2024) | 3 (n = 1133) | Italy | 6; 7 | 1; 2 | 1 | 1 | 1 | 2 | 1 | 1 |
| 162. Maillard et al. (2024) | 2 | — | 7 | 1 | 1 | 1 | 1 | 1 | 2 | 5 |
| 163. Mansour et al. (2024) | 3 (n = 27) | USA | 5 | 2; 3 | 1 | 1 | 1 | 7 | 3 | 8 |
| 164. Manzella et al. (2025) | 2 | — | 7 | 1 | 1 | 1 | 1 | 2 | 2 | 5 |
| 165. Marshall et al. (2024) | 3 (n = 100) | USA | 5; 7 | 1; 3 | 1 | 1; 2 | 1 | 6 | 1 | 2 |
| 166. McMahon et al. (2024) | 1 | — | 7 | 1 | 1 | 1 | 1 | 4 | 1 | 1 |
| 167. Mendel et al. (2025) | 3 (n = 281) | USA | 6 | 2 | 1 | 1; 2; 5 | 1 | 6 | 1 | 1 |
| 168. Miskiewicz et al. (2024) | 1 | — | 3; 7 | 1 | 1 | 1 | 1 | 2 | 1 | 1 |
| 169. Mondal et al. (2023) | 2 | — | 3; 7 | 1 | 1 | 1 | 1 | 2 | 1 | 1 |
| 170. Montastruc et al. (2023) | 2 | — | 7 | 1 | 1 | 1 | 1 | 6 | 2 | 5 |
| 171. Montiel-Romero et al. (2025) | 2 | — | 7 | 1 | 1 | 1 | 1 | 1 | 2 | 5 |
| 172. Moskatel et al. (2023) | 1 | — | 7 | 1 | 1 | 1 | 1 | 2 | 2 | 9 |
| 173. Motegi et al. (2025) | 1 | — | 7 | 1 | 1 | 1 | 1 | 2 | 1 | 1 |
| 174. Moya-Salazar et al. (2024) | 3 (n = 2) | Peru | 1 | 4 | 1 | 1 | 1 | 2 | 3 | 9 |
| 175. Naeem et al. (2025) | 2 | — | 7 | 1 | 1 | 1 | 1 | 6 | 2 | 5 |
| 176. Nastasi et al. (2023) | 2 | — | 7 | 1 | 1 | 1 | 1 | 2 | 1 | 1 |
| 177. Neo et al. (2024) | 2 | — | 7 | 1 | 1 | 1; 2 | 1 | 2 | 1 | 4 |
| 178. Nickel et al. (2025) | 2 | — | 7 | 1 | 1 | 1 | 1 | 2 | 1 | 4 |
| 179. Nitsch et al. (2025) | 3 (n = 35) | USA | 5; 6 | 2 | 1 | 1; 2 | 1 | 2 | 1 | 1 |
| 180. Norris et al. (2025) | 3 (n = 101) | Multiple: Australia; New Zealand | 6 | 2 | 1 | 1 | 1 | 6 | 2 | 6 |
| 181. Nov et al. (2023) | 3 (n = 392) | USA | 5; 6; 7 | 1; 2; 3 | 1 | 1 | 1 | 6 | 1 | 1 |
| 182. Ozkan et al. (2025) | 3 (n = 115) | Multiple: Canada; China; Croatia; France; Italy; Russia; Saudi Arabia; Tanzania; Turkey; United States; other countries not individually reported | 6 | 2 | 1 | 1 | 1 | 6 | 2; 3 | 5; 8; 9 |
| 183. Parekh et al. (2024) | 1 | — | 7 | 1 | 1 | 1; 2 | 1 | 2 | 1 | 1 |
| 184. Patel et al. (2024) | 3 (n = 48) | USA | 5; 7 | 1; 3 | 1 | 1 | 1 | 2 | 2; 3 | 8 |
| 185. Paulo et al. (2025) | 3 (n = 11) | Portugal | 1 | 2 | 1 | 1 | 2 | 2 | 2 | 5 |
| 186. Perlis et al. (2024) | 2 | — | 7 | 1 | 1 | 1 | 1 | 3 | 2 | 5 |
| 187. Pham et al. (2024) | 1 | — | 7 | 1 | 1 | 1 | 1 | 2 | 2 | 5 |
| 188. Philuek et al. (2025) | 3 (n = 9) | Thailand | 5 | 3 | 1 | 1 | 1 | 5 | 1 | 4 |
| 189. Piazza et al. (2024) | 2 | — | 7 | 1 | 1 | 1 | 1 | 2 | 2 | 5 |
| 190. Picard et al. (2025) | 1 | — | 3; 7; 8 | 1 | 1 | 1 | 1 | 2 | 3 | 9 |
| 191. Platt et al. (2024) | 3 (n = 1787) | USA | 6 | 2 | 1 | 1 | 2 | 6 | 1 | 1 |
| 192. Pratibha et al. (2023) | 3 (n = 20) | India | 7 | 1; 4 | 1 | 1 | 2 | 2 | 2 | 9 |
| 193. Qin et al. (2024) | 3 (n = 734) | China | 1; 5; 7 | 1; 2 | 1 | 1 | 1 | 6 | 1 | 1 |
| 194. Qu et al. (2024) | 3 (n = 955) | China | 6 | 2 | 3 | 8 | 4 | 6 | 2 | 5 |
| 195. Rackoff et al. (2025) | 3 (n = 428) | USA | 6 | 2 | 1 | 8 | 2 | 3 | 1 | 3 |
| 196. Rädel-Ablass et al. (2025) | 3 (n = 28) | Unspecified | 6; 7 | 1; 2; 3 | 1 | 1 | 1 | 6 | 3 | 8 |
| 197. Rames et al. (2024) | 2 | — | 7 | 1 | 1 | 1 | 1 | 8 | 1 | 1 |
| 198. Rao et al. (2025) | 2 | — | 7 | 1 | 1 | 1 | 1 | 2 | 3 | 8 |
| 199. Rawashdeh et al. (2023) | 2 | — | 7 | 1 | 1 | 1 | 1 | 8 | 2 | 9 |
| 200. Rebitschek et al. (2025) | 3 (n = 300) | UK | 5; 7 | 1; 2; 3 | 1 | 1; 3; 7 (Mistral) | 1 | 2 | 1 | 1 |
| 201. Reinhardt et al. (2025) | 3 (n = 1951) | Multiple: Austria; Denmark; France; Serbia | 5 | 2 | 1 | 8 | 1 | 6 | 1 | 1 |
| 202. Reynolds et al. (2024) | 2 | — | 7 | 1 | 1 | 1 | 1 | 2 | 1 | 1 |
| 203. Riddell et al. (2023) | 1 | — | 3; 7 | 1 | 1 | 1 | 1 | 2 | 1 | 1 |
| 204. Rizwan et al. (2023) | 1 | — | 7 | 1 | 1 | 1 | 1 | 2 | 2 | 5 |
| 205. Robinson et al. (2023) | 1 | — | 7 | 1 | 1 | 1 | 1 | 2 | 2 | 6 |
| 206. Rodler et al. (2025) | 2 | — | 3; 7 | 1 | 1 | 1 | 1 | 2 | 1 | 1 |
| 207. Rodriguez et al. (2024) | 3 (n = 11) | USA | 1; 6; 7 | 1; 2 | 1 | 1 | 1 | 2 | 2; 3 | 9 |
| 208. Romoff et al. (2025) | 2 | — | 3; 7 | 1 | 1 | 1; 3; 5 | 1 | 2 | 1 | 1 |
| 209. Roosan et al. (2024) | 2 | — | 7 | 1 | 1 | 1 | 1 | 2 | 2 | 5 |
| 210. Roy et al. (2025) | 2 | — | 3; 7 | 1 | 1 | 1 | 1 | 2 | 1 | 1 |
| 211. Ruiz Sarrias et al. (2024) | 2 | — | 7 | 1 | 1 | 1 | 1 | 2 | 2 | 5 |
| 212. Saeidnia et al. (2024) | 3 (n = 30) | Iran | 1; 6; 7 | 1; 2 | 1 | 1 | 1 | 2 | 1 | 1 |
| 213. Salam et al. (2024) | 3 (n = 13) | Germany | 5; 7 | 1; 3 | 1 | 1 | 1 | 2 | 1 | 1 |
| 214. Sallam et al. (2024) | 3 (n = 164) | Jordan | 6 | 2 | 1 | 1; 3; 5 | 1 | 3 | 3 | 8 |
| 215. Sallam et al. (2025) | 3 (n = 587) | Multiple: Egypt; Iraq; Jordan; Kuwait; Saudi Arabia; other countries not individually reported | 6 | 2 | 1 | 1 | 1 | 3 | 3 | 8 |
| 216. Samsonov et al. (2024) | 1 | — | 7 | 1 | 1 | 1 | 1 | 2 | 1 | 1 |
| 217. Sanchez Tena et al. (2025) | 3 (n = 225) | Multiple: Portugal; Spain | 6; 7 | 1; 2 | 1 | 1; 2 | 1 | 2 | 2; 3 | 8 |
| 218. Saraç et al. (2025) | 2 | — | 7 | 1 | 1 | 1; 3 | 1 | 5 | 1 | 4 |
| 219. Schäfer et al. (2025) | 3 (n = 527) | Multiple: Germany; United Kingdom; United States; other countries not individually reported | 6 | 2; 3 | 2 | 7 (Clare) | 3 | 3 | 1 | 3 |
| 220. Schlarb et al. (2024) | 3 (n = 4) | Germany | 1; 6; 7 | 1; 2 | 1 | 1 | 1 | 5 | 1 | 4 |
| 221. Schmidt et al. (2024) | 3 (n = 184) | Multiple: countries not individually reported | 1; 6 | 2 | 1 | 8 | 1 | 6 | 2 | 6 |
| 222. Schmidt et al. (2025) | 3 (n = 265) | Netherlands | 5; 7 | 1; 3 | 1 | 1 | 1 | 6 | 2 | 5 |
| 223. Segal et al. (2023) | 3 (n = 1) | USA | 7 | 1; 4 | 1 | 1 | 1 | 2 | 2 | 6 |
| 224. Senoymak et al. (2024) | 2 | — | 7 | 1 | 1 | 1 | 1 | 2 | 1 | 1 |
| 225. Senturk et al. (2025) | 2 | — | 3; 7 | 1 | 1 | 1 | 1 | 3 | 1 | 1 |
| 226. Shahsavar et al. (2023) | 3 (n = 607) | Unspecified | 6 | 2 | 1 | 1 | 1 | 6 | 1 | 2 |
| 227. Sheikh et al. (2024) | 1 | — | 7 | 1 | 1 | 1 | 2 | 2 | 1 | 1 |
| 228. Shikino et al. (2024) | 2 | — | 7 | 1 | 1 | 1 | 1 | 6 | 2 | 5 |
| 229. Simsek et al. (2025) | 3 (n = 441) | Turkey | 6 | 2 | 1 | 8 | 2 | 6 | 3 | 8 |
| 230. Sireci et al. (2024) | 2 | — | 7 | 1 | 1 | 1 | 1 | 2 | 2 | 5 |
| 231. Small et al. (2024) | 3 (n = 16) | USA | 3; 6; 7 | 1; 2 | 2 | 1; 7 (Epic Generated Draft Replies) | 1 | 6 | 1; 2 | 1; 7 |
| 232. Smith et al. (2024) | 2 | — | 7 | 1 | 1 | 1 | 1 | 2 | 1 | 1 |
| 233. Solmonovich et al. (2025) | 3 (n = 562) | USA | 6 | 2 | 1 | 1 | 1 | 6 | 2 | 6; 8; 9 |
| 234. Soto-Chávez et al. (2024) | 1 | — | 7 | 1 | 1 | 1 | 1 | 2 | 1 | 1 |
| 235. Spatscheck et al. (2024) | 3 (n = 368) | Multiple: countries not individually reported | 5 | 2 | 2 | 7 (task-specific models) | 1 | 6 | 3 | 5 |
| 236. Spies et al. (2023) | 3 (n = 8) | USA | 6; 7 | 1; 2 | 1 | 1 | 1 | 6 | 2 | 5 |
| 237. Spina et al. (2025) | 1 | — | 3; 7 | 1 | 1 | 1 | 1 | 2 | 1 | 1 |
| 238. Spotnitz et al. (2024) | 3 (n = 30) | USA | 6 | 2 | 1 | 8 | 1 | 6 | 2 | 5 |
| 239. Stage et al. (2025) | 3 (n = 41) | USA | 6; 7 | 1; 2 | 1 | 1 | 1 | 6 | 3 | 9 |
| 240. Stalp et al. (2024) | 2 | — | 7 | 1 | 1 | 1 | 1 | 2 | 2 | 5 |
| 241. Stanceski et al. (2024) | 2 | — | 7 | 1 | 1 | 1 | 1 | 6 | 1 | 4 |
| 242. Stephan et al. (2025) | 3 (n = 300) | Germany | 3; 5; 7 | 1; 2 | 1 | 1 | 1 | 6 | 1 | 1 |
| 243. Stoneham et al. (2024) | 1 | — | 7 | 1 | 1 | 1 | 1 | 2 | 2 | 5 |
| 244. Sultan et al. (2023) | 1 | — | 7 | 1 | 1 | 1 | 1 | 2 | 2 | 5 |
| 245. Sun et al. (2025) | 3 (n = 20) | Netherlands | 1; 5 | 3 | 1 | 1 | 2 | 6 | 1 | 1 |
| 246. Taha et al. (2024) | 3 (n = 428) | Egypt | 6 | 2 | 1 | 1 | 1 | 6 | 2 | 5 |
| 247. Tai-Seale et al. (2024) | 3 (n = 122) | USA | 5 | 2; 3; 4 | 2 | 7 (Epic GenAI draft replies) | 1 | 6 | 2; 4 | 7; 10 |
| 248. Tangadulrat et al. (2023) | 3 (n = 239) | Thailand | 6 | 2 | 1 | 1 | 1 | 2 | 2; 3 | 8 |
| 249. Tao et al. (2024) | 3 (n = 693) | China | 6 | 2 | 1 | 1 | 4 | 6 | 3 | 8 |
| 250. Tekin et al. (2025) | 3 (n = 196) | Turkey | 5; 7 | 1; 3 | 1 | 1; 3 | 2 | 6 | 3 | 8 |
| 251. Tenner et al. (2024) | 1 | — | 7 | 1 | 1 | 1 | 2 | 6 | 2 | 5 |
| 252. Thota et al. (2025) | 2 | — | 7 | 1 | 3 | 8 | 1 | 6 | 2 | 5 |
| 253. Tong et al. (2025) | 3 (n = 23) | China | 2 | 2 | 1 | 8 | 1 | 6 | 2 | 9 |
| 254. Tortella et al. (2025) | 3 (n = 589) | Italy | 6 | 2 | 1 | 1; 2; 6 | 1 | 6 | 3 | 8 |
| 255. Triantafyllopoulos et al. (2024) | 3 (n = 182) | Greece | 6 | 3 | 1 | 1 | 1 | 6 | 2 | 5 |
| 256. Triplett et al. (2025) | 2 | — | 7 | 1 | 1 | 1 | 1 | 6 | 2 | 5 |
| 257. Tuncer et al. (2024) | 3 (n = 288) | Turkey | 6 | 2 | 1 | 1 | 1 | 6 | 2 | 5 |
| 258. Tuppal et al. (2025) | 3 (n = 10) | Philippines | 2 | 2 | 1 | 1 | 1 | 6 | 3 | 9 |
| 259. Uribe et al. (2024) | 3 (n = 428) | Multiple: Argentina; Brazil; Chile; Colombia; Egypt; France; India; Latvia; Lithuania; Mexico; Spain; United Kingdom; United States; Venezuela; other countries not individually reported | 6 | 2 | 1 | 1 | 1 | 6 | 3 | 8 |
| 260. Valetopoulou et al. (2024) | 3 (n = 60) | UK | 5 | 1; 2 | 1 | 1 | 1 | 6 | 2; 3 | 8 |
| 261. Vasudevan et al. (2025) | 3 (n = 512) | Saudi Arabia | 6 | 2 | 1 | 1 | 1 | 6 | 3 | 8 |
| 262. Verran (2024) | 1 | — | 3; 7 | 1 | 1 | 1 | 1 | 2 | 1; 2 | 1; 7 |
| 263. Vidal Sabanés et al. (2025) | 2 | — | 3; 7 | 1 | 1 | 1 | 1 | 2 | 1; 3 | 1; 9 |
| 264. von Bubnoff et al. (2025) | 2 | — | 3; 7 | 1 | 2 | 7 (unnamed Reddit-analysis application) | 1 | 2 | 3 | 9 |
| 265. Vu et al. (2025) | 3 (n = 165) | USA | 6 | 2 | 1 | 1 | 1 | 6 | 1 | 1 |
| 266. Wang et al. (2024) | 3 (n = 12) | China | 2; 5 | 3 | 1 | 1 | 2 | 3 | 1 | 3 |
| 267. Wiemken et al. (2024) | 1 | — | 7 | 1 | 1 | 1; 7 (Mixtral) | 1 | 1 | 2 | 5 |
| 268. Wu et al. (2024) | 3 (n = 900) | UK | 6 | 2 | 1 | 8 | 2 | 2 | 1 | 2 |
| 269. Wu et al. (2025) | 2 | — | 7 | 1 | 1 | 1 | 1 | 1 | 1; 2 | 1; 7 |
| 270. Xia et al. (2025) | 3 (n = 60) | China | 5 | 2 | 1 | 1 | 1 | 1 | 1 | 4 |
| 271. Xie (2025) | 3 (n = 600) | China | 5 | 2 | 2 | 7 (unnamed university-provided chatbot) | 1 | 6 | 1 | 1 |
| 272. Xu et al. (2025) | 2 | — | 7 | 1 | 1 | 1 | 1 | 4 | 1 | 1 |
| 273. Yan et al. (2025) | 2 | — | 7 | 1 | 1 | 1 | 1 | 2 | 1 | 1 |
| 274. Yanagita et al. (2024) | 2 | — | 7 | 1 | 1 | 1 | 1 | 6 | 2 | 8 |
| 275. Yang et al. (2025) | 3 (n = 9) | China | 5; 7 | 1; 2 | 1 | 1 | 1 | 2 | 1; 2 | 1; 7 |
| 276. Yang et al. (2025) | 3 (n = 20) | China | 1 | 2 | 2 | 7 (iflyhealth) | 1 | 2 | 1; 4 | 4; 10 |
| 277. Yang et al. (2025) | 2 | — | 7 | 1 | 1 | 1 | 1 | 2 | 2 | 5 |
| 278. Yazla et al. (2025) | 1 | — | 7 | 1 | 1 | 1 | 1 | 2 | 2 | 5 |
| 279. Yigit et al. (2025) | 3 (n = 10) | Turkey | 1 | 2 | 1 | 1 | 1 | 6 | 3 | 8 |
| 280. Yildiz et al. (2023) | 2 | — | 7 | 1 | 1 | 1 | 1 | 2 | 1 | 1 |
| 281. Ying et al. (2024) | 2 | — | 7 | 1 | 1 | 1 | 1 | 2 | 1; 2 | 1; 5 |
| 282. Yun et al. (2025) | 3 (n = 297) | Multiple: Australia; Canada; Germany; Mexico; Poland; Portugal; South Africa; United Kingdom; United States; other countries not individually reported | 6 | 2 | 1 | 1; 5; 7 (Amazon Alexa, Apple Siri, Perplexity AI) | 1 | 6 | 1 | 1 |
| 283. Zeng et al. (2024) | 2 | — | 7 | 1 | 1 | 1; 3; 4; 5; 7 (ERNIE Bot) | 1 | 1 | 1 | 1 |
| 284. Zhang et al. (2024) | 2 | — | 7 | 1 | 1 | 1 | 1 | 2 | 2 | 5 |
| 285. Zhang et al. (2024) | 3 (n = 1038) | Multiple: Argentina; Australia; Brazil; Egypt; France; Ghana; Malaysia; Mexico; Namibia; Nigeria; Pakistan; Peru; Saudi Arabia; Serbia; South Africa; Spain; Thailand; Turkey; United Arab Emirates; United Kingdom; United States | 6 | 2 | 1 | 1 | 1 | 6 | 1 | 1 |
| 286. Zhou et al. (2025) | 3 (n = 137) | China | 5; 7 | 1; 2 | 1 | 1 | 1 | 6 | 2 | 8 |

**References for Supplementary Table S1**

1. Abate, A., Poncato, E., Barbieri, M. A., Powell, G., Rossi, A., Peker, S., Hviid, A., Bate, A., & Sessa, M. (2025). Off-the-shelf large language models for causality assessment of individual case safety reports: A proof-of-concept with COVID-19 vaccines. *Drug Safety*, *48*(7), 805–820. https://doi.org/10.1007/s40264-025-01531-y

2. Abdelgadir, Y. H., Thongprayoon, C., Craici, I. M., Cheungpasitporn, W., & Miao, J. (2025). Improving patient understanding of glomerular disease terms with ChatGPT. *International Journal of Clinical Practice*, *2025*(1), 9977290. https://doi.org/10.1155/ijcp/9977290

3. Abouammoh, N., Alhasan, K., Aljamaan, F., Raina, R., Malki, K. H., Altamimi, I., Muaygil, R., Wahabi, H., Jamat, A., Alhaboob, A., Assiri, R. A., Al-Tawfiq, J. A., Al-Eyadhy, A., Soliman, M., & Temsah, M. H. (2025). Perceptions and earliest experiences of medical students and faculty with ChatGPT in medical education: Qualitative study. *JMIR Medical Education*, *11*, e63400. https://doi.org/10.2196/63400

4. Abu Hammour, K., Alhamad, H., Al-Ashwal, F. Y., Halboup, A., Abu Farha, R., & Abu Hammour, A. (2023). ChatGPT in pharmacy practice: A cross-sectional exploration of Jordanian pharmacists' perception, practice, and concerns. *Journal of Pharmaceutical Policy and Practice*, *16*(1), 115. https://doi.org/10.1186/s40545-023-00624-2

5. Abu Hammour, A., Hammour, K. A., Alhamad, H., Nassar, R., El-Dahiyat, F., Sawaqed, M., Allan, A., Manaseer, Q., Abu Hammour, M., Halboup, A., & Farha, R. A. (2024). Exploring Jordanian medical students' perceptions and concerns about ChatGPT in medical education: A cross-sectional study. *Journal of Pharmaceutical Policy and Practice*, *17*(1), 2429000. https://doi.org/10.1080/20523211.2024.2429000

6. Ajlouni, A. O., Almahaireh, A. S., & Wahba, F. A.-A. (2023). Students' perception of using ChatGPT in counseling and mental health education: The benefits and challenges. *International Journal of Emerging Technologies in Learning (iJET)*, *18*(20), 199–218. https://doi.org/10.3991/ijet.v18i20.42075

7. Aközlü, Z., & Erkut, Z. (2024). Can artificial intelligence assist nurses in planning the nursing care of a child with acute lymphoblastic leukemia? *Indian Journal of Medical and Paediatric Oncology*, *45*(06), 523–526. https://doi.org/10.1055/s-0044-1788701

8. Al-Anezi, F. M. (2024). Exploring the use of ChatGPT as a virtual health coach for chronic disease management. *Learning Health Systems*, *8*(3), e10406. https://doi.org/10.1002/lrh2.10406

9. Al Shboul, M., Alwreikat, A., & Alotaibi, F. A. (2024). Investigating the use of ChatGPT as a novel method for seeking health information: A qualitative approach. *Science & Technology Libraries*, *43*(3), 225–234. https://doi.org/10.1080/0194262x.2023.2250835

10. Al Tibi, G., Alexander, M., Miller, S., & Chronos, N. (2024). A retrospective comparison of medication recommendations between a cardiologist and ChatGPT-4 for hypertension patients in a rural clinic. *Cureus*, *16*, e55789. https://doi.org/10.7759/cureus.55789

11. Alam, S. F., Thongprayoon, C., Miao, J., Pham, J. H., Sheikh, M. S., Valencia, O. A. G., Schwartz, G. L., Craici, I. M., Suarez, M. L. G., & Cheungpasitporn, W. (2025). Advancing personalized medicine in digital health: The role of artificial intelligence in enhancing clinical interpretation of 24-h ambulatory blood pressure monitoring. *Digital Health*, *11*, 20552076251326014. https://doi.org/10.1177/20552076251326014

12. Alanezi, F. (2024). Assessing the effectiveness of ChatGPT in delivering mental health support: A qualitative study. *Journal of Multidisciplinary Healthcare*, *17*, 461–471. https://doi.org/10.2147/jmdh.s447368

13. Alanezi, F. (2024). Factors influencing patients' engagement with ChatGPT for accessing health-related information. *Critical Public Health*, *34*(1), 1–20. https://doi.org/10.1080/09581596.2024.2348164

14. Alanzi, T. M. (2023). Impact of ChatGPT on teleconsultants in healthcare: Perceptions of healthcare experts in Saudi Arabia. *Journal of Multidisciplinary Healthcare*, *16*, 2309–2321. https://doi.org/10.2147/jmdh.s419847

15. Alanzi, T. M., Alzahrani, W., Albalawi, N. S., Allahyani, T., Alghamdi, A., Al-Zahrani, H., Almutairi, A., Alzahrani, H., Almulhem, L., Alanzi, N., Al Moarfeg, A., & Farhah, N. (2023). Public awareness of obesity as a risk factor for cancer in central Saudi Arabia: Feasibility of ChatGPT as an educational intervention. *Cureus*, *15*, e50781. https://doi.org/10.7759/cureus.50781

16. Albogami, Y., Alfakhri, A., Alaqil, A., Alkoraishi, A., Alshammari, H., Elsharawy, Y., Alhammad, A., & Alhossan, A. (2024). Safety and quality of AI chatbots for drug-related inquiries: A real-world comparison with licensed pharmacists. *Digital Health*, *10*, 20552076241253523. https://doi.org/10.1177/20552076241253523

17. Ali, R., Connolly, I. D., Tang, O. Y., Mirza, F. N., Johnston, B., Abdulrazeq, H. F., Galamaga, P. F., Libby, T. J., Sodha, N. R., Groff, M. W., Gokaslan, Z. L., Telfeian, A. E., Shin, J. H., Asaad, W. F., Zou, J., & Doberstein, C. E. (2024). Bridging the literacy gap for surgical consents: An AI-human expert collaborative approach. *npj Digital Medicine*, *7*(1), 63. https://doi.org/10.1038/s41746-024-01039-2

18. Aliyeva, A., Sari, E., Alaskarov, E., & Nasirov, R. (2024). Enhancing postoperative cochlear implant care with ChatGPT-4: A study on artificial intelligence (AI)-assisted patient education and support. *Cureus*, *16*, e53897. https://doi.org/10.7759/cureus.53897

19. Alkhamees, A. (2024). Evaluation of artificial intelligence as a search tool for patients: Can ChatGPT-4 provide accurate evidence-based orthodontic-related information? *Cureus*, *16*, e65820. https://doi.org/10.7759/cureus.65820

20. Allen, T., Horwitz, A., & Sprigle, S. (2025). Leveraging AI and customer reviews to evaluate technology used by people with disabilities. *Disability and Rehabilitation: Assistive Technology*, *20*(6), 1668–1676. https://doi.org/10.1080/17483107.2025.2465603

21. Alruwaili, A. N., Alshammari, A. M., Alhaiti, A., Elsharkawy, N. B., Ali, S. I., & Ramadan, O. M. E. (2025). Neonatal nurses' experiences with generative AI in clinical decision-making: A qualitative exploration in high-risk nicus. *BMC Nursing*, *24*(1), 386. https://doi.org/10.1186/s12912-025-03044-6

22. Arbanas, G., Perisa, A., Biliskov, I., Susac, J., Badurina, M., & Arbanas, D. (2025). Patients prefer human psychiatrists over chatbots: A cross-sectional study. *Croatian Medical Journal*, *66*(1), 13–19. https://doi.org/10.3325/cmj.2025.66.13

23. Argan, M., Gürbüz, B., Dursun, M. T., Dinç, H., & Tokay Argan, M. (2024). Usage intention of ChatGPT for health in Turkey: An extended technology acceptance model. *International Journal of Human–Computer Interaction*, *41*(15), 9492–9504. https://doi.org/10.1080/10447318.2024.2426045

24. Armbruster, J., Bussmann, F., Rothhaas, C., Titze, N., Gruetzner, P. A., & Freischmidt, H. (2024). "Doctor ChatGPT, can you help me?" The patient's perspective: Cross-sectional study. *Journal of Medical Internet Research*, *26*, e58831. https://doi.org/10.2196/58831

25. Armitage, R. C. (2025). How do GPs want large language models to be applied in primary care, and what are their concerns? A cross‐sectional survey. *Journal of Evaluation in Clinical Practice*, *31*(4), e70129. https://doi.org/10.1111/jep.70129

26. Armstrong, D., Paul, C., McGlaughlin, B., & Hill, D. (2024). Can artificial intelligence (AI) educate your patient? A study to assess overall readability and pharmacists' perception of AI-generated patient education materials. *Jaccp: Journal of the American College of Clinical Pharmacy*, *7*(8), 803–808. https://doi.org/10.1002/jac5.2006

27. Arvidsson, R., Gunnarsson, R., Entezarjou, A., Sundemo, D., & Wikberg, C. (2024). ChatGPT (GPT-4) versus doctors on complex cases of the Swedish family medicine specialist examination: An observational comparative study. *BMJ Open*, *14*(12), e086148. https://doi.org/10.1136/bmjopen-2024-086148

28. Aster, A., Ragaller, S. V., Raupach, T., & Marx, A. (2025). ChatGPT as a virtual patient: Written empathic expressions during medical history taking. *Medical Science Educator*, *35*(3), 1513–1522. https://doi.org/10.1007/s40670-025-02342-7

29. Ataguba, G., & Orji, R. (2025). Exploring large language models for personalized recipe generation and weight-loss management. *ACM Transactions on Computing for Healthcare*, *6*(2), 1–57. https://doi.org/10.1145/3712709

30. Ayers, J. W., Poliak, A., Dredze, M., Leas, E. C., Zhu, Z., Kelley, J. B., Faix, D. J., Goodman, A. M., Longhurst, C. A., Hogarth, M., & Smith, D. M. (2023). Comparing physician and artificial intelligence chatbot responses to patient questions posted to a public social media forum. *JAMA Internal Medicine*, *183*(6), 589. https://doi.org/10.1001/jamainternmed.2023.1838

31. Ayo-Ajibola, O., Davis, R. J., Lin, M. E., Riddell, J., & Kravitz, R. L. (2024). Characterizing the adoption and experiences of users of artificial intelligence–generated health information in the United States: Cross-sectional questionnaire study. *Journal of Medical Internet Research*, *26*, e55138. https://doi.org/10.2196/55138

32. Ayo-Ajibola, O., Julien, C., Lin, M. E., Riddell, J., Duan, N. H., & Kravitz, R. L. (2025). Association of primary care access with health‐related ChatGPT use: A national cross‐sectional survey. *Journal of General Internal Medicine*, *41*(2), 338–345. https://doi.org/10.1007/s11606-025-09406-9

33. Ayoub, M., Ballout, A. A., Zayek, R. A., & Ayoub, N. F. (2023). Mind + machine: ChatGPT as a basic clinical decisions support tool. *Cureus*, *15*, e43690. https://doi.org/10.7759/cureus.43690

34. Ayre, J., Mac, O., McCaffery, K., McKay, B. R., Liu, M. Y., Shi, Y., Rezwan, A., & Dunn, A. G. (2024). New frontiers in health literacy: Using ChatGPT to simplify health information for people in the community. *Journal of General Internal Medicine*, *39*(4), 573–577. https://doi.org/10.1007/s11606-023-08469-w

35. Başaran, F., & Duru, P. (2024). The impact of Kahoot and ChatGPT educational technologies on nursing students’ sexual health knowledge and attitudes: A quasi‐experimental study. *Sexuality and Disability*, *42*(4), 801–815. https://doi.org/10.1007/s11195-024-09873-8

36. Beckmann, S. A., Link, E., & Bachl, M. (2025). “ChatGPT, is the influenza vaccination useful?” Comparing perceived argument strength and correctness of pro-vaccination arguments from AI and medical experts. *Journal of Science Communication*, *24*(2). https://doi.org/10.22323/2.24020204

37. Benfatah, M., Marfak, A., Saad, E., Hilali, A., Nejjari, C., & Youlyouz-Marfak, I. (2024). Assessing the efficacy of ChatGPT as a virtual patient in nursing simulation training: A study on nursing students' experience. *Teaching and Learning in Nursing*, *19*(3), e486–e493. https://doi.org/10.1016/j.teln.2024.02.005

38. Berend, K., Duits, A., & Gans, R. O. B. (2025). Challenging cases of hyponatremia incorrectly interpreted by ChatGPT. *BMC Medical Education*, *25*(1), 751. https://doi.org/10.1186/s12909-025-07235-2

39. Bernstein, I. A., Zhang, Y. C., Govil, D., Majid, I., Chang, R. T., Sun, Y., Shue, A., Chou, J. C., Schehlein, E., Christopher, K. L., Groth, S. L., Ludwig, C., & Wang, S. Y. (2023). Comparison of ophthalmologist and large language model chatbot responses to online patient eye care questions. *JAMA Network Open*, *6*(8), e2330320. https://doi.org/10.1001/jamanetworkopen.2023.30320

40. Bhattacharyya, M., Miller, V. M., Bhattacharyya, D., & Miller, L. E. (2023). High rates of fabricated and inaccurate references in ChatGPT-generated medical content. *Cureus*, *15*, e39238. https://doi.org/10.7759/cureus.39238

41. Blease, C. R., Locher, C., Gaab, J., Hägglund, M., & Mandl, K. D. (2024). Generative artificial intelligence in primary care: An online survey of UK general practitioners. *BMJ Health & Care Informatics*, *31*(1), e101102. https://doi.org/10.1136/bmjhci-2024-101102

42. Bohn, B., & Anselmann, V. (2024). Artificial intelligence in nursing practice – a delphi study with ChatGPT. *Applied Nursing Research*, *80*, 151867. https://doi.org/10.1016/j.apnr.2024.151867

43. Boonrit, N., Chaisawat, K., Phueakong, C., Nootong, N., & Ruanglertboon, W. (2024). Exploring community pharmacists’ attitudes in Thailand towards ChatGPT usage: A pilot qualitative investigation. *Digital Health*, *10*, 20552076241283256. https://doi.org/10.1177/20552076241283256

44. Brigo, F., Broggi, S., Strigaro, G., Olivo, S., Tommasini, V., Massar, M., Turcato, G., & Zaboli, A. (2025). Artificial intelligence (ChatGPT 4.0) vs. human expertise for epileptic seizure and epilepsy diagnosis and classification in adults: An exploratory study. *Epilepsy & Behavior*, *166*, 110364. https://doi.org/10.1016/j.yebeh.2025.110364

45. Brigo, F., Broggi, S., Leuci, E., Turcato, G., & Zaboli, A. (2025). Can ChatGPT 4.0 diagnose epilepsy? A study on artificial intelligence's diagnostic capabilities. *Journal of Clinical Medicine*, *14*(2), 322. https://doi.org/10.3390/jcm14020322

46. Buholayka, M., Zouabi, R., & Tadinada, A. (2023). The readiness of ChatGPT to write scientific case reports independently: A comparative evaluation between human and artificial intelligence. *Cureus*, *15*, e39386. https://doi.org/10.7759/cureus.39386

47. Bundy, H., Gerhart, J., Baek, S., Connor, C. D., Isreal, M., Dharod, A., Stephens, C., Liu, T. L., Hetherington, T., & Cleveland, J. (2024). Can the administrative loads of physicians be alleviated by AI-facilitated clinical documentation? *Journal of General Internal Medicine*, *39*(15), 2995–3000. https://doi.org/10.1007/s11606-024-08870-z

48. Burford, K. G., Itzkowitz, N. G., Ortega, A. G., Teitler, J. O., & Rundle, A. G. (2024). Use of generative AI to identify helmet status among patients with micromobility-related injuries from unstructured clinical notes. *JAMA Network Open*, *7*(8), e2425981. https://doi.org/10.1001/jamanetworkopen.2024.25981

49. Bushuven, S., Bentele, M., Bentele, S., Gerber, B., Bansbach, J., Ganter, J., Trifunovic-Koenig, M., & Ranisch, R. (2023). “ChatGPT, can you help me save my child’s life?” - diagnostic accuracy and supportive capabilities to lay rescuers by ChatGPT in prehospital basic life support and paediatric advanced life support cases – an in-silico analysis. *Journal of Medical Systems*, *47*(1), 123. https://doi.org/10.1007/s10916-023-02019-x

50. Buz, M., & Demirhan, R. (2024). Large language model and medical education: Evaluation of human and artificial intelligence responses to thoracic surgery questions. *Southern Clinics of Istanbul Eurasia*, *35*(3), 209–212. https://doi.org/10.14744/scie.2024.91129

51. Campellone, T. R., Flom, M., Montgomery, R. M., Bullard, L., Pirner, M. C., Pavez, A., Morales, M., Harper, D., Oddy, C., O'Connor, T., Daniels, J., Eaneff, S., Forman-Hoffman, V. L., Sackett, C., & Darcy, A. (2025). Safety and user experience of a generative artificial intelligence digital mental health intervention: Exploratory randomized controlled trial. *Journal of Medical Internet Research*, *27*, e67365. https://doi.org/10.2196/67365

52. Chandler, R. D., Warner, S., Aidoo-Frimpong, G., & Wells, J. (2024). “What did you say, ChatGPT?” The use of AI in Black women’s HIV self-education: An inductive qualitative data analysis. *Journal of the Association of Nurses in AIDS Care*, *35*(3), 294–302. https://doi.org/10.1097/jnc.0000000000000468

53. Chandler, R. D., Warner, S., Guillaume, D., & Wells, J. (2025). Exploring ChatGPT's role in women's health self-education: A descriptive study comparing responses with public health guidance. *Nursing Outlook*, *73*(4), 102468. https://doi.org/10.1016/j.outlook.2025.102468

54. Chang, A., Young, J., Para, A., Lamb, A., & Gulati, N. (2024). Efficacy of ChatGPT in educating patients and clinicians about skin toxicities associated with cancer treatment. *JMIR Dermatology*, *7*, e54919. https://doi.org/10.2196/54919

55. Chen, S. Y., Kuo, H. Y., & Chang, S. H. (2024). Perceptions of ChatGPT in healthcare: Usefulness, trust, and risk. *Frontiers in Public Health*, *12*, 1457131. https://doi.org/10.3389/fpubh.2024.1457131

56. Chen, P. J., & Liou, W. K. (2025). ChatGPT-driven interactive virtual reality communication simulation in obstetric nursing: A mixed-methods study. *Nurse Education in Practice*, *85*, 104383. https://doi.org/10.1016/j.nepr.2025.104383

57. Chen, C., & Cui, Z. H. (2025). Impact of AI-assisted diagnosis on American patients’ trust in and intention to seek help from health care professionals: Randomized, web-based survey experiment. *Journal of Medical Internet Research*, *27*, e66083. https://doi.org/10.2196/66083

58. Cheng, S. L., Xiao, Y. P., Liu, L., & Sun, X. Y. (2024). Comparative outcomes of AI-assisted ChatGPT and face-to-face consultations in infertility patients: A cross-sectional study. *Postgraduate Medical Journal*, *100*(1189), 851–855. https://doi.org/10.1093/postmj/qgae083

59. Choi, J., Kim, J. W., Lee, Y. S., Tae, J. H., Choi, S. Y., Chang, I. H., & Kim, J. H. (2024). Availability of ChatGPT to provide medical information for patients with kidney cancer. *Scientific Reports*, *14*(1), 1542. https://doi.org/10.1038/s41598-024-51531-8

60. Chou, H. H., Chen, Y. H., Lin, C. T., Chang, H. T., Wu, A. C., Tsai, J. L., Chen, H. W., Hsu, C. C., Liu, S. Y., & Lee, J. T. (2025). Ai‐driven patient support: Evaluating the effectiveness of ChatGPT‐4 in addressing queries about ovarian cancer compared with healthcare professionals in gynecologic oncology. *Supportive Care in Cancer*, *33*(4), 337. https://doi.org/10.1007/s00520-025-09389-7

61. Choudhury, A., Elkefi, S., & Tounsi, A. (2024). Exploring factors influencing user perspective of ChatGPT as a technology that assists in healthcare decision making: A cross sectional survey study. *PLOS One*, *19*(3), e0296151. https://doi.org/10.1371/journal.pone.0296151

62. Choudhury, A., Shahsavar, Y., & Shamszare, H. (2025). User intent to use DeepSeek for health care purposes and their trust in the large language model: Multinational survey study. *JMIR Human Factors*, *12*, e72867. https://doi.org/10.2196/72867

63. Christofi, A., Neophytou, R., & Liasidou, S. (2025). Using artificial intelligence to address sexually transmitted infections during and after travel of young travellers. *Worldwide Hospitality and Tourism Themes*, *17*(1), 91–105. https://doi.org/10.1108/whatt-12-2024-0309

64. Colakca, C., Ergin, M., Ozensoy, H. S., Sener, A., Guru, S., & Ozhasenekler, A. (2024). Emergency department triaging using ChatGPT based on emergency severity index principles: A cross-sectional study. *Scientific Reports*, *14*(1), 22106. https://doi.org/10.1038/s41598-024-73229-7

65. Cosma, C., Radi, A., Cattano, R., Zanobini, P., Bonaccorsi, G., Lorini, C., & Del Riccio, M. (2025). Potential role of ChatGPT in simplifying and improving informed consent forms for vaccination: A pilot study conducted in italy. *BMJ Health & Care Informatics*, *32*(1), e101248. https://doi.org/10.1136/bmjhci-2024-101248

66. Creswell Báez, J., Victor, B. G., Dysart, C., & Goldkind, L. (2025). “I don’t understand it, but okay”: An empirical study of mental health practitioners’ readiness to use large language models. *Journal of Technology in Human Services*, *43*(2), 142–160. https://doi.org/10.1080/15228835.2025.2479475

67. Criss, S., Nguyen, T. T., Gonzales, S. M., Lin, B., Kim, M., Makres, K., Sorial, B. M., Xiong, Y. J., Dennard, E., Merchant, J. S., & Hswen, Y. (2024). "HIV stigma exists" - exploring ChatGPT's HIV advice by race and ethnicity, sexual orientation, and gender identity. *Journal of Racial and Ethnic Health Disparities*, *12*(6), 3622–3635. https://doi.org/10.1007/s40615-024-02162-2

68. Dal, E., Srivastava, A., Chigarira, B., Chehade, C. H., Thomas, V. M., Fortuna, G. M. G., Garg, D., Ji, R., Gebrael, G., Agarwal, N., Swami, U., & Li, H. R. (2024). Effectiveness of ChatGPT 4.0 in telemedicine-based management of metastatic prostate carcinoma. *Diagnostics*, *14*(17), 1899. https://doi.org/10.3390/diagnostics14171899

69. de Ruiter, E. J., Eimermann, V. M., Rijcken, C., Taxis, K., & Borgsteede, S. D. (2025). The extent and type of use, opportunities and concerns of ChatGPT in community pharmacy: A survey of community pharmacy staff. *Exploratory Research in Clinical and Social Pharmacy*, *17*, 100575. https://doi.org/10.1016/j.rcsop.2025.100575

70. Dolev-Cohen, M., & Henry, N. (2025). Teachers vs. chat GPT: Identifying and naming acts of online sexual abuse. *Information, Communication & Society*, *28*(16), 2976–2994. https://doi.org/10.1080/1369118x.2025.2492585

71. Dos Santos, F. C., Johnson, L. G., Madandola, O. O., Priola, K. J. B., Yao, Y. W., Macieira, T. G. R., & Keenan, G. M. (2024). An example of leveraging AI for documentation: ChatGPT-generated nursing care plan for an older adult with lung cancer. *Journal of the American Medical Informatics Association*, *31*(9), 2089–2096. https://doi.org/10.1093/jamia/ocae116

72. Du, K., Li, A., Zuo, Q. H., Zhang, C. Y., Guo, R., Chen, P., Du, W. S., & Li, S. M. (2025). Comparing artificial intelligence–generated and clinician-created personalized self-management guidance for patients with knee osteoarthritis: Blinded observational study. *Journal of Medical Internet Research*, *27*, e67830. https://doi.org/10.2196/67830

73. Dubinski, D., Won, S. Y., Trnovec, S., Behmanesh, B., Baumgarten, P., Dinc, N., Konczalla, J., Chan, A. L., Bernstock, J. D., Freiman, T. M., & Gessler, F. (2024). Leveraging artificial intelligence in neurosurgery—unveiling ChatGPT for neurosurgical discharge summaries and operative reports. *Acta Neurochirurgica*, *166*(1), 38. https://doi.org/10.1007/s00701-024-05908-3

74. Dzuali, F., Seiger, K., Novoa, R., Aleshin, M., Teng, J., Lester, J., & Daneshjou, R. (2024). ChatGPT may improve access to language-concordant care for patients with non–English language preferences. *JMIR Medical Education*, *10*, e51435. https://doi.org/10.2196/51435

75. Ebrahimi, N., Vakhshoori, M., Teichman, S., & Abdipour, A. (2025). Agreement between AI and nephrologists in addressing common patient questions about diabetic nephropathy: Cross-sectional study. *JMIR Diabetes*, *10*, e65846. https://doi.org/10.2196/65846

76. El Haj, M., Boutoleau-Bretonnière, C., Gallouj, K., Wagemann, N., Antoine, P., Kapogiannis, D., & Chapelet, G. (2024). ChatGPT as a diagnostic aid in alzheimer’s disease: An exploratory study. *Journal of Alzheimer's Disease Reports*, *8*(1), 495–500. https://doi.org/10.3233/adr-230191

77. Elpasiony, N. M. A., Sabek, E. M., & Ibrahim, S. S. M. (2025). Chat generative pre-trained transformers era: Pros and cons between nursing researchers in egypt. *BMC Nursing*, *24*(1), 667. https://doi.org/10.1186/s12912-025-03332-1

78. Erdat, E. C., & Senler, F. Ç. (2025). Turkish medical oncologists’ perspectives on integrating artificial intelligence: Knowledge, attitudes, and ethical considerations. *BMC Medical Ethics*, *26*(1), 82. https://doi.org/10.1186/s12910-025-01249-7

79. Evans, K., Papinniemi, A., Ploderer, B., Nicholson, V., Hindhaugh, T., Vuvan, V., Cowley, N., Tariq, A., & Thomson, H. (2025). Impact of using an AI scribe on clinical documentation and clinician-patient interactions in allied health private practice: Perspectives of clinicians and patients. *Musculoskeletal Science and Practice*, *78*, 103333. https://doi.org/10.1016/j.msksp.2025.103333

80. Fukuzawa, F., Yanagita, Y., Yokokawa, D., Uchida, S., Yamashita, S., Li, Y., Shikino, K., Tsukamoto, T., Noda, K., Uehara, T., & Ikusaka, M. (2024). Importance of patient history in artificial intelligence- assisted medical diagnosis: Comparison study. *JMIR Medical Education*, *10*, e52674. https://doi.org/10.2196/52674

81. Galido, P. V., Butala, S., Chakerian, M., & Agustines, D. (2023). A case study demonstrating applications of ChatGPT in the clinical management of treatment-resistant schizophrenia. *Cureus*, *15*, e38166. https://doi.org/10.7759/cureus.38166

82. Garcia, P., Ma, S. P., Shah, S. Y., Smith, M., Jeong, Y., Devon-Sand, A., Tai-Seale, M., Takazawa, K., Clutter, D., Vogt, K., Lugtu, C., Rojo, M., Lin, S. V., Shanafelt, T., Pfeffer, M. A., & Sharp, C. (2024). Artificial intelligence-generated draft replies to patient inbox messages. *JAMA Network Open*, *7*(3), e243201. https://doi.org/10.1001/jamanetworkopen.2024.3201

83. Gencer, B., Arzu, U., Orhan, S. S., Dincal, T., & Ekinci, M. (2025). Evaluation of ChatGPT responses about sexual activity after total hip arthroplasty: A comparative study with observers of different experience levels. *Journal of Clinical Medicine*, *14*(9), 2942. https://doi.org/10.3390/jcm14092942

84. Genes, M., & Deveci, B. (2024). A clinical evaluation of cardiovascular emergencies: A comparison of responses from ChatGPT, emergency physicians, and cardiologists. *Diagnostics*, *14*(23), 2731. https://doi.org/10.3390/diagnostics14232731

85. Gill, B., Bonamer, J., Kuechly, H., Gupta, R., Emmert, S., Kurkowski, S., Hasselfeld, K., & Grawe, B. (2024). ChatGPT is a promising tool to increase readability of orthopedic research consents. *Journal of Orthopaedics, Trauma and Rehabilitation*, *31*(2), 148–152. https://doi.org/10.1177/22104917231208212

86. Giray, L. (2025). Cases of using ChatGPT as a mental health and psychological support tool. *Journal of Consumer Health on the Internet*, *29*(1), 29–48. https://doi.org/10.1080/15398285.2024.2442374

87. Girton, M. R., Greene, D. N., Messerlian, G., Keren, D. F., & Yu, M. (2024). ChatGPT vs medical professional: Analyzing responses to laboratory medicine questions on social media. *Clinical Chemistry*, *70*(9), 1122–1139. https://doi.org/10.1093/clinchem/hvae093

88. Goh, E., Gallo, R., Hom, J., Strong, E., Weng, Y. J., Kerman, H., Cool, J. A., Kanjee, Z., Parsons, A. S., Ahuja, N., Horvitz, E., Yang, D. N., Milstein, A., Olson, A. P. J., Rodman, A., & Chen, J. H. (2024). Large language model influence on diagnostic reasoning a randomized clinical trial. *JAMA Network Open*, *7*(10), e2440969. https://doi.org/10.1001/jamanetworkopen.2024.40969

89. Gondode, P., Duggal, S., Garg, N., Sethupathy, S., Asai, O., & Lohakare, P. (2024). Comparing patient education tools for chronic pain medications: Artificial intelligence chatbot versus traditional patient information leaflets. *Indian Journal of Anaesthesia*, *68*(7), 631–636. https://doi.org/10.4103/ija.ija_204_24

90. Guleria, S., Guptill, J., Kumar, I., McClintic, M., & Rojas, J. C. (2024). Artificial intelligence integration in healthcare: Perspectives and trends in a survey of U. S. health system leaders. *BMC Digital Health*, *2*(1), 80. https://doi.org/10.1186/s44247-024-00135-3

91. Gürses, Ö. A., Özüdogru, A., Tuncay, F., & Kararti, C. (2025). The role of artificial intelligence large language models in personalized rehabilitation programs for knee osteoarthritis: An observational study. *Journal of Medical Systems*, *49*(1), 73. https://doi.org/10.1007/s10916-025-02207-x

92. Habicht, J., Dina, L. M., McFadyen, J., Stylianou, M., Harper, R., Hauser, T. U., & Rollwage, M. (2025). Generative AI-enabled therapy support tool for improved clinical outcomes and patient engagement in group therapy: Real-world observational study. *Journal of Medical Internet Research*, *27*, e60435. https://doi.org/10.2196/60435

93. Hamed, E., Eid, A., & Alberry, M. (2023). Exploring ChatGPT's potential in facilitating adaptation of clinical guidelines: A case study of diabetic ketoacidosis guidelines. *Cureus*, *15*, e38784. https://doi.org/10.7759/cureus.38784

94. Hanai, A., Ishikawa, T., Kawauchi, S., Iida, Y., & Kawakami, E. (2024). Generative artificial intelligence and non-pharmacological bias: An experimental study on cancer patient sexual health communications. *BMJ Health & Care Informatics*, *31*(1), e100924. https://doi.org/10.1136/bmjhci-2023-100924

95. Harari, R. E., Altaweel, A., Ahram, T., Keehner, M., & Shokoohi, H. (2025). A randomized controlled trial on evaluating clinician-supervised generative AI for decision support. *International Journal of Medical Informatics*, *195*, 105701. https://doi.org/10.1016/j.ijmedinf.2024.105701

96. Hasei, J., Hanzawa, M., Nagano, A., Maeda, N., Yoshida, S., Endo, M., Yokoyama, N., Ochi, M., Ishida, H., Katayama, H., Fujiwara, T., Nakata, E., Nakahara, R., Kunisada, T., Tsukahara, H., & Ozaki, T. (2025). Empowering pediatric, adolescent, and young adult patients with cancer utilizing generative AI chatbots to reduce psychological burden and enhance treatment engagement: A pilot study. *Frontiers in Digital Health*, *7*, 1543543. https://doi.org/10.3389/fdgth.2025.1543543

97. He, Z., Bhasuran, B., Jin, Q., Tian, S. B., Hanna, K., Shavor, C., Arguello, L. G., Murray, P., & Lu, Z. Y. (2024). Quality of answers of generative large language models versus peer users for interpreting laboratory test results for lay patients: Evaluation study. *Journal of Medical Internet Research*, *26*, e56655. https://doi.org/10.2196/56655

98. Hernandez, C. A., Vazquez Gonzalez, A. E., Polianovskaia, A., Amoro Sanchez, R., Muyolema Arce, V., Mustafa, A., Vypritskaya, E., Perez Gutierrez, O., Bashir, M., & Eighaei Sedeh, A. (2023). The future of patient education: AI-driven guide for type 2 diabetes. *Cureus*, *15*(11), e48919. https://doi.org/10.7759/cureus.48919

99. Hipgrave, L., Goldie, J., Dennis, S., & Coleman, A. (2025). Balancing risks and benefits: Clinicians’ perspectives on the use of generative AI chatbots in mental healthcare. *Frontiers in Digital Health*, *7*, 1606291. https://doi.org/10.3389/fdgth.2025.1606291

100. Hoj, S., Thomsen, S. F., Meteran, H., Sigsgaard, T., & Meteran, H. (2023). Artificial intelligence and allergic rhinitis: Does ChatGPT increase or impair the knowledge? *Journal of Public Health*, *46*(1), 123–126. https://doi.org/10.1093/pubmed/fdad219

101. Hose, B. Z., Rounds, A. K., Nandwani, I., Busog, D. N., Giardina, T. D., Haskell, H., Smith, K. M., & Miller, K. E. (2025). Use of ChatGPT for urinary symptom management among people with spinal cord injury or disease: Qualitative study. *JMIR Rehabilitation and Assistive Technologies*, *12*, e70339. https://doi.org/10.2196/70339

102. Huang, H., & Lin, H.-C. (2024). ChatGPT as a life coach for professional identity formation in medical education: A self-regulated learning perspective. *Educational Technology & Society*, *27*(3), 374–389. https://doi.org/10.30191/ets.202407_27(3).tp03

103. Huang, T., Safranek, C., Socrates, V., Chartash, D., Wright, D., Dilip, M., Sangal, R. B., & Taylor, R. A. (2024). Patient-representing population's perceptions of GPT-generated versus standard emergency department discharge instructions: Randomized blind survey assessment. *Journal of Medical Internet Research*, *26*, e60336. https://doi.org/10.2196/60336

104. Huang, Y. H., Wang, W. J., Zhou, J. Y., Zhang, L., Lin, J. H., Liu, H., Hu, X. G., Zhou, Z. K., & Dong, W. H. (2025). Integrative modeling enables ChatGPT to achieve average level of human counselors performance in mental health Q&A. *Information Processing & Management*, *62*(5), 104152. https://doi.org/10.1016/j.ipm.2025.104152

105. Huo, B. G., Mckechnie, T., Ortenzi, M., Lee, Y., Antoniou, S., Mayol, J., Ahmed, H., Boudreau, V., Ramji, K., & Eskicioglu, C. (2024). Dr. GPT will see you now: The ability of large language model-linked chatbots to provide colorectal cancer screening recommendations. *Health and Technology*, *14*(3), 463–469. https://doi.org/10.1007/s12553-024-00836-9

106. Jain, G., Pareek, S., & Carlbring, P. (2024). Revealing the source: How awareness alters perceptions of AI and human-generated mental health responses. *Internet Interventions*, *36*, 100745. https://doi.org/10.1016/j.invent.2024.100745

107. Jairoun, A. A., Al-Hemyari, S. S., Shahwan, M., Al-Qirim, T., & Shahwan, M. (2024). Benefit–risk assessment of ChatGPT applications in the field of diabetes and metabolic illnesses: A qualitative study. *Clinical Medicine Insights: Endocrinology and Diabetes*, *17*, 11795514241235514. https://doi.org/10.1177/11795514241235514

108. Ji, Z., Jiang, Y., Sun, H., Qiu, B., Chen, Y., Li, M., Fan, J., & Wang, J. (2024). Enhancing puncture skills training with generative AI and digital technologies: A parallel cohort study. *BMC Medical Education*, *24*(1), 1328. https://doi.org/10.1186/s12909-024-06217-0

109. Ju, H., Park, M., Jeong, H., Lee, Y., Kim, H., Seong, M., & Lee, D. (2025). Generative AI-based nursing diagnosis and documentation recommendation using virtual patient electronic record data. *Healthcare Informatics Research*, *31*(2), 156–165. https://doi.org/10.4258/hir.2025.31.2.156

110. Juhi, A., Pipil, N., Santra, S., Mondal, S., Behera, J. K., & Mondal, H. (2023). The capability of ChatGPT in predicting and explaining common drug-drug interactions. *Cureus*, *15*, e36272. https://doi.org/10.7759/cureus.36272

111. Kaneda, Y., Takita, M., Hamaki, T., Ozaki, A., & Tanimoto, T. (2023). ChatGPT's potential in enhancing physician efficiency: A Japanese case study. *Cureus*, *15*, e48235. https://doi.org/10.7759/cureus.48235

112. Kang, B., & Hong, M. (2025). Development and evaluation of a mental health chatbot using ChatGPT4.0: Mixed methods user experience study with Korean users. *JMIR Medical Informatics*, *13*, e63538. https://doi.org/10.2196/63538

113. Karaçay, P., Goktas, P., Yasar, O., Uyanik, B., Uzlu, S., Coskun, K., & Benk, M. (2025). Investigation of pressure injuries with visual ChatGPT integration: A descriptive cross-sectional study. *Journal of Advanced Nursing*, *82*(1), 479–492. https://doi.org/10.1111/jan.16905

114. Kharko, A., McMillan, B., Hagström, J., Muli, I., Davidge, G., Hägglund, M., & Blease, C. (2024). Generative artificial intelligence writing open notes: A mixed methods assessment of the functionality of GPT 3.5 and GPT 4.0. *Digital Health*, *10*, 20552076241291384. https://doi.org/10.1177/20552076241291384

115. Kim, S. H., Tae, J. H., Chang, I. H., Kim, T. H., Myung, S. C., Nguyen, T. T., Choi, J., Kim, J. H., Kim, J. W., Lee, Y. S., & Choi, S. Y. (2023). Changes in patient perceptions regarding ChatGPT-written explanations on lifestyle modifications for preventing urolithiasis recurrence. *Digital Health*, *9*, 20552076231203940. https://doi.org/10.1177/20552076231203940

116. Kim, J., Chen, M. L., Rezaei, S. J., Hernandez-Boussard, T., Chen, J. H., Rodriguez, F., Han, S. S., Lal, R. A., Kim, S. H., Dosiou, C., Seav, S. M., Akcan, T., Rodriguez, C. I., Asch, S. M., & Linos, E. (2025). Artificial intelligence tools in supporting healthcare professionals for tailored patient care. *npj Digital Medicine*, *8*(1), 210. https://doi.org/10.1038/s41746-025-01604-3

117. Kim, M. S., Lee, J. S., & Bae, H. (2025). Large language models for pre-mediation counseling in medical disputes: A comparative evaluation against human experts. *Healthcare Informatics Research*, *31*(2), 200–208. https://doi.org/10.4258/hir.2025.31.2.200

118. Kishimoto, T., Hao, X. M., Chang, T. M., & Luo, Z. Y. (2025). Single online self-compassion writing intervention reduces anxiety: With the feedback of ChatGPT. *Internet Interventions*, *39*, 100810. https://doi.org/10.1016/j.invent.2025.100810

119. Koegel, L. K., Ponder, E., Bruzzese, T., Wang, M., Semnani, S. J., Chi, N., Koegel, B. L., Lin, T. Y., Swarnakar, A., & Lam, M. S. (2025). Using artificial intelligence to improve empathetic statements in autistic adolescents and adults: A randomized clinical trial. *Journal of Autism and Developmental Disorders*, *56*(7), 2513–2529. https://doi.org/10.1007/s10803-025-06734-x

120. Koh, M. C. Y., Ngiam, J. N., Yong, J. Y., Tambyah, P. A., & Archuleta, S. (2024). The role of an artificial intelligence model in antiretroviral therapy counselling and advice for people living with HIV. *HIV Medicine*, *25*(4), 504–508. https://doi.org/10.1111/hiv.13604

121. Koh, M. C. Y., Ngiam, J. N., Salada, B. M. A., Tambyah, P. A., Archuleta, S., & Oon, J. E. L. (2025). Can ChatGPT counter vaccine hesitancy? An evaluation of ChatGPT's responses to simulated queries from the general public. *Healthcare*, *13*(11), 1269. https://doi.org/10.3390/healthcare13111269

122. Koh, M. C. Y., Ngiam, J. N., Oon, J. E. L., Lum, L. H. W., Smitasin, N., & Archuleta, S. (2025). Using ChatGPT for writing hospital inpatient discharge summaries – perspectives from an inpatient infectious diseases service. *BMC Health Services Research*, *25*(1), 221. https://doi.org/10.1186/s12913-025-12373-w

123. Kuo, F. H., Fierstein, J. L., Tudor, B. H., Gray, G. M., Ahumada, L. M., Watkins, S. C., & Rehman, M. A. (2024). Comparing ChatGPT and a single anesthesiologist's responses to common patient questions: An exploratory cross-sectional survey of a panel of anesthesiologists. *Journal of Medical Systems*, *48*(1), 77. https://doi.org/10.1007/s10916-024-02100-z

124. Lang, S. G., Vitale, J., Fekete, T. F., Haschtmann, D., Reitmeir, R., Ropelato, M., Puhakka, J., Galbusera, F., & Loibl, M. (2024). Are large language models valid tools for patient information on lumbar disc herniation? The spine surgeons' perspective. *Brain and Spine*, *4*, 102804. https://doi.org/10.1016/j.bas.2024.102804

125. Lang, S. G., Vitale, J., Galbusera, F., Fekete, T., Boissiere, L., Charles, Y. P., Yucekul, A., Yilgor, C., Núñez-Pereira, S., Haddad, S., Gomez-Rice, A., Mehta, J., Pizones, J., Pellisé, F., Obeid, I., Alanay, A., Kleinstück, F., Loibl, M., & Grp, E. S. S. G. E. S. S. (2025). Is the information provided by large language models valid in educating patients about adolescent idiopathic scoliosis? An evaluation of content, clarity, and empathy: The perspective of the European spine study group. *Spine Deformity*, *13*(2), 361–372. https://doi.org/10.1007/s43390-024-00955-3

126. Latt, P. M., Aung, E. T., Htaik, K., Soe, N. N., Lee, D., King, A. J., Fortune, R., Ong, J. J., Chow, E. P. F., Bradshaw, C. S., Rahman, R., Deneen, M., Dobinson, S., Randall, C., Zhang, L., & Fairley, C. K. (2025). Evaluation of artificial intelligence (AI) chatbots for providing sexual health information: A consensus study using real-world clinical queries. *BMC Public Health*, *25*(1), 1788. https://doi.org/10.1186/s12889-025-22933-8

127. Lauderdale, S., Griffin, S. A., Lahman, K. R., Mbaba, E., & Tomlinson, S. (2025). Unveiling public stigma for borderline personality disorder: A comparative study of artificial intelligence and mental health care providers. *Personality and Mental Health*, *19*(2), e70018. https://doi.org/10.1002/pmh.70018

128. Lee, J., Park, J., & Han, H. S. (2024). Using ChatGPT for kidney transplantation: Perceived information quality by race and education levels. *Clinical Transplantation*, *38*(7), e15378. https://doi.org/10.1111/ctr.15378

129. Levin, C., Suliman, M., Naimi, E., & Saban, M. (2025). Augmenting intensive care unit nursing practice with generative AI: A formative study of diagnostic synergies using simulation-based clinical cases. *Journal of Clinical Nursing*, *34*(7), 2898–2907. https://doi.org/10.1111/jocn.17384

130. Levit, N. S., & Saban, M. (2025). When investigator meets large language models: A qualitative analysis of cancer patient decision-making journeys. *npj Digital Medicine*, *8*(1), 336. https://doi.org/10.1038/s41746-025-01747-3

131. Levkovich, I., & Elyoseph, Z. (2023). Identifying depression and its determinants upon initiating treatment: ChatGPT versus primary care physicians. *Family Medicine and Community Health*, *11*(4), e002391. https://doi.org/10.1136/fmch-2023-002391

132. Levkovich, I., Rabin, E., Brann, M., & Elyoseph, Z. (2024). Large language models outperform general practitioners in identifying complex cases of childhood anxiety. *Digital Health*, *10*, 20552076241294182. https://doi.org/10.1177/20552076241294182

133. Leypold, T., Lingens, L. F., Beier, J. P., & Boos, A. M. (2024). Integrating AI in lipedema management: Assessing the efficacy of GPT-4 as a consultation assistant. *Life*, *14*(5), 646. https://doi.org/10.3390/life14050646

134. Li, M., Xiong, X. M., & Xu, B. (2024). Attitudes and perceptions of Chinese oncologists towards artificial intelligence in healthcare: A cross-sectional survey. *Frontiers in Digital Health*, *6*, 1371302. https://doi.org/10.3389/fdgth.2024.1371302

135. Li, K. D., Fernandez, A. M., Schwartz, R., Rios, N., Carlisle, M. N., Amend, G. M., Patel, H., & Breyer, B. N. (2024). Comparing GPT-4 and human researchers in health care data analysis: Qualitative description study. *Journal of Medical Internet Research*, *26*, e56500. https://doi.org/10.2196/56500

136. Li, S. S., Chen, M., Liu, P. L., & Xu, J. (2024). Following medical advice of an AI or a human doctor? Experimental evidence based on clinician-patient communication pathway model. *Health Communication*, *40*(9), 1810–1822. https://doi.org/10.1080/10410236.2024.2423114

137. Li, Y., Chen, L., & Fu, L. (2024). Vicarious interaction in online health consultation service: The effects of generative AI’s anthropomorphism and social support on intended responses through social presence and source credibility. *International Journal of Human–Computer Interaction*, *41*(17), 11209–11226. https://doi.org/10.1080/10447318.2024.2441422

138. Lian, C. X., Yuan, X., Chokkakula, S., Wang, G. Q., Song, B., Wang, Z., Fan, G., & Yin, C. L. (2024). Assessing the ChatGPT aptitude: A competent and effective dermatology doctor? *Heliyon*, *10*(17), e37220. https://doi.org/10.1016/j.heliyon.2024.e37220

139. Liao, W., Liu, Z., Dai, H., Xu, S., Wu, Z., Zhang, Y., Huang, X., Zhu, D., Cai, H., Li, Q., Liu, T., & Li, X. (2023). Differentiating ChatGPT-generated and human-written medical texts: Quantitative study. *JMIR Medical Education*, *9*, e48904. https://doi.org/10.2196/48904

140. Lim, S., & Schmälzle, R. (2023). Artificial intelligence for health message generation: An empirical study using a large language model (LLM) and prompt engineering. *Frontiers in Communication*, *8*, 1129082. https://doi.org/10.3389/fcomm.2023.1129082

141. Lim, B., Seth, I., Kah, S., Sofiadellis, F., Ross, R. J., Rozen, W. M., & Cuomo, R. (2023). Using generative artificial intelligence tools in cosmetic surgery: A study on rhinoplasty, facelifts, and blepharoplasty procedures. *Journal of Clinical Medicine*, *12*(20), 6524. https://doi.org/10.3390/jcm12206524

142. Lin, C. C. J., Krzy, E. Z., Tsai, S. H. L., Wang, Y. C., Chang, C. W., Lin, T. Y., & Fu, T. S. (2025). A comparative analysis between ChatGPT versus NASS clinical guidelines for adult isthmic spondylolisthesis. *North American Spine Society Journal (NASSJ)*, *22*, 100599. https://doi.org/10.1016/j.xnsj.2025.100599

143. Lin, H. L., Liao, L. L., Wang, Y. N., & Chang, L. C. (2025). Attitude and utilization of ChatGPT among registered nurses: A cross-sectional study. *International Nursing Review*, *72*(2), e13012. https://doi.org/10.1111/inr.13012

144. Linardon, J., Liu, C., Messer, M., Mcclure, Z., Anderson, C., & Jarman, H. K. (2025). Current practices and perspectives of artificial intelligence in the clinical management of eating disorders: Insights from clinicians and community participants. *International Journal of Eating Disorders*, *58*(4), 724–734. https://doi.org/10.1002/eat.24385

145. Lindbäck, Y., Schröder, K., Engström, T., Valeskog, K., & Sonesson, S. (2025). Generative artificial intelligence in physiotherapy education: Great potential amidst challenges- a qualitative interview study. *BMC Medical Education*, *25*(1), 603. https://doi.org/10.1186/s12909-025-07106-w

146. Liu, J. Y., Zheng, J. Q., Cai, X. T., Wu, D. D., & Yin, C. L. (2023). A descriptive study based on the comparison of ChatGPT and evidence-based neurosurgeons. *iScience*, *26*(9), 107590. https://doi.org/10.1016/j.isci.2023.107590

147. Liu, S. R., Wright, A. P., Patterson, B. L., Wanderer, J. P., Turer, R. W., Nelson, S. D., McCoy, A. B., Sittig, D. F., & Wright, A. (2023). Using AI-generated suggestions from ChatGPT to optimize clinical decision support. *Journal of the American Medical Informatics Association*, *30*(7), 1237–1245. https://doi.org/10.1093/jamia/ocad072

148. Liu, S. R., McCoy, A. B., Wright, A. P., Nelson, S. D., Huang, S. S., Ahmad, H. B., Carro, S. E., Franklin, J., Brogan, J., & Wright, A. (2024). Why do users override alerts? Utilizing large language model to summarize comments and optimize clinical decision support. *Journal of the American Medical Informatics Association*, *31*(6), 1388–1396. https://doi.org/10.1093/jamia/ocae041

149. Liu, J., Daher, M., Laperche, J., Gilreath, N., Testa, E. J., El-Othamni, M. M., Barrett, T. J., & Antoci, V. (2025). ChatGPT versus expert arthroplasty surgeons in total knee arthroplasty patient counseling. *The Knee*, *55*, 12–17. https://doi.org/10.1016/j.knee.2025.03.005

150. Liu, J., Chen, X. H., Liu, C. Z., & Han, P. (2025). Factors influencing adoption intentions to use AIGC for health information: Findings from SEM and fsQCA. *Frontiers in Public Health*, *13*, 1525879. https://doi.org/10.3389/fpubh.2025.1525879

151. Liu, Y., & Wang, F. (2025). Investigating the interpretability of ChatGPT in mental health counseling: An analysis of artificial intelligence generated content differentiation. *Computer Methods and Programs in Biomedicine*, *268*, 108864. https://doi.org/10.1016/j.cmpb.2025.108864

152. Liu, J. B., Wang, L., He, X. H., Xia, Y. R., Gong, X. Y., Wu, R. J., Li, S., & Wu, L. L. (2025). Leveraging generative artificial intelligence to enhance ICU novice simulation instructors’ case design: A qualitative study. *Clinical Simulation in Nursing*, *105*, 101770. https://doi.org/10.1016/j.ecns.2025.101770

153. Lockwood, A., Farmer, R., Shergill, G., Benson, N., & Gilbert, K. (2025). Human vs. machine: Comparing AI-generated and human-written psychological reports. *Journal of Psychoeducational Assessment*, *43*(6), 559–573. https://doi.org/10.1177/07342829251346623

154. Logan, J. A., Sadhu, S., Hazlewood, C., Denton, M., Burke, S. E., Simone-Soule, C. A., Black, C., Ciaverelli, C., Stulb, J., Nourzadeh, H., Vinogradskiy, Y., Leader, A., Dicker, A. P., Choi, W., & Simone, N. L. (2025). Bridging gaps in cancer care: Utilizing large language models for accessible dietary recommendations. *Nutrients*, *17*(7), 1176. https://doi.org/10.3390/nu17071176

155. Lu, X. J., Gao, X. Q., Wang, X. Y., Gong, Z. Y., Cheng, J., Hu, W. G., Wu, S., Wang, R., & Li, X. Y. (2025). Comparison of medical history documentation efficiency and quality based on GPT-4o: A study on the comparison between residents and artificial intelligence. *Frontiers in Medicine*, *12*, 1545730. https://doi.org/10.3389/fmed.2025.1545730

156. Lu, H. (2025). Generative AI for vaccine misbelief correction: Insights from targeting extraversion and pseudoscientific beliefs. *Vaccine*, *54*, 127018. https://doi.org/10.1016/j.vaccine.2025.127018

157. Luo, X. F., Wang, B. Y., Li, Y. L., Liu, S., Li, H. D., Ren, Y. X., Yang, W. H., Lam, K., Ali, S. R., Sharp, G., Moraes, F. Y., Wang, Y., Zhu, D., Yang, Z. H., Mohammad, D., Fruscio, R., Boulos, M. N. K., Lin, Z. C., Ide, K., …, Grp, A. W. (2025). Knowledge and awareness of generative artificial intelligence use in medicine among international stakeholders: A cross-sectional study. *Journal of Evidence-Based Medicine*, *18*(2), e70034. https://doi.org/10.1111/jebm.70034

158. Lv, X. L., Zhang, X. M., Li, Y., Ding, X. X., Lai, H. C., & Shi, J. Y. (2024). Leveraging large language models for improved patient access and self-management: Assessor-blinded comparison between expert- and AI-generated content. *Journal of Medical Internet Research*, *26*, e55847. https://doi.org/10.2196/55847

159. Ma, Y. Z., Zeng, Y., Liu, T., Sun, R. S., Xiao, M. Z., & Wang, J. (2024). Integrating large language models in mental health practice: A qualitative descriptive study based on expert interviews. *Frontiers in Public Health*, *12*, 1475867. https://doi.org/10.3389/fpubh.2024.1475867

160. Ma, Y. Z., Liu, T., Qi, J. W., Gan, Y. L., Cheng, Q. Y., Xiao, M. Z., & Wang, J. (2025). Facilitators and barriers of large language model adoption among nursing students: A qualitative descriptive study. *Journal of Advanced Nursing*, *81*(8), 4856–4870. https://doi.org/10.1111/jan.16655

161. Maida, E., Moccia, M., Palladino, R., Borriello, G., Affinito, G., Clerico, M., Repice, A. M., Di Sapio, A., Iodice, R., Spiezia, A. L., Sparaco, M., Miele, G., Bile, F., Scandurra, C., Ferraro, D., Stromillo, M. L., Docimo, R., De Martino, A., Mancinelli, L., …, SIN, I. S. N. (2024). ChatGPT vs. neurologists: A cross‐sectional study investigating preference, satisfaction ratings and perceived empathy in responses among people living with multiple sclerosis. *Journal of Neurology*, *271*(7), 4057–4066. https://doi.org/10.1007/s00415-024-12328-x

162. Maillard, A., Micheli, G., Lefevre, L., Guyonnet, C., Poyart, C., Canoui, E., Belan, M., & Charlier, C. (2024). Can chatbot artificial intelligence replace infectious diseases physicians in the management of bloodstream infections? A prospective cohort study. *Clinical Infectious Diseases*, *78*(4), 825–832. https://doi.org/10.1093/cid/ciad632

163. Mansour, T., & Wong, J. H. (2024). Enhancing fieldwork readiness in occupational therapy students with generative AI. *Frontiers in Medicine*, *11*, 1485325. https://doi.org/10.3389/fmed.2024.1485325

164. Manzella, R., Immordino, A., Galletti, C., Indaco, F. G., Stilo, G., Messina, G., Lorusso, F., Gargano, R., Frangipane, S., Giunta, G., Mariut, D., Portelli, D., Zambito, P., Ferrisi, M. G., Ciodaro, F., Centineo, M., Maira, S., Dispenza, F., Gallina, S., …, Sireci, F. (2025). ChatGPT in the management of chronic rhinosinusitis with nasal polyps: Promising support or digital illusion? Insights from a multicenter observational study. *Journal of Clinical Medicine*, *14*(13), 4501. https://doi.org/10.3390/jcm14134501

165. Marshall, C., Forbes, J., Seidman, M. D., Roldan, L., & Atkins, J. (2024). Artificial intelligence for diagnosis in otologic patients: Is it ready to be your doctor? *Otology & Neurotology*, *45*(8), 863–869. https://doi.org/10.1097/mao.0000000000004267

166. McMahon, H. V., & McMahon, B. D. (2024). Automating untruths: ChatGPT, self-managed medication abortion, and the threat of misinformation in a post-roe world. *Frontiers in Digital Health*, *6*, 1287186. https://doi.org/10.3389/fdgth.2024.1287186

167. Mendel, T., Singh, N., Mann, D. M., Wiesenfeld, B., & Nov, O. (2025). Laypeople's use of and attitudes toward large language models and search engines for health queries: Survey study. *Journal of Medical Internet Research*, *27*, e64290. https://doi.org/10.2196/64290

168. Miskiewicz, M. J., Leonardo, C., Capotosto, S., Ling, K. Y., Cohen, D., Komatsu, D., & Wang, E. D. (2024). Can generative artificial intelligence enhance health literacy about lateral epicondylitis? *Cureus*, *16*, e61384. https://doi.org/10.7759/cureus.61384

169. Mondal, H., Mondal, S., & Podder, I. (2023). Using ChatGPT for writing articles for patients’ education for dermatological diseases: A pilot study. *Indian Dermatology Online Journal*, *14*(4), 482. https://doi.org/10.4103/idoj.idoj_72_23

170. Montastruc, F., Storck, W., de Canecaude, C., Victor, L., Li, J., Cesbron, C., Zelmat, Y., & Barus, R. (2023). Will artificial intelligence chatbots replace clinical pharmacologists? An exploratory study in clinical practice. *European Journal of Clinical Pharmacology*, *79*(10), 1375–1384. https://doi.org/10.1007/s00228-023-03547-8

171. Montiel-Romero, S., Rajme-López, S., Román-Montes, C. M., López-Iñiguez, A., Rivera-Villegas, H. O., Ochoa-Hein, E., González-Lara, M. F., Ponce-de-León, A., Tamez-Torres, K. M., & Martinez-Guerra, B. A. (2025). Recommended antibiotic treatment agreement between infectious diseases specialists and ChatGPT®. *BMC Infectious Diseases*, *25*(1), 38. https://doi.org/10.1186/s12879-024-10426-9

172. Moskatel, L. S., & Zhang, N. S. (2023). The utility of ChatGPT in the assessment of literature on the prevention of migraine: An observational, qualitative study. *Frontiers in Neurology*, *14*, 1225223. https://doi.org/10.3389/fneur.2023.1225223

173. Motegi, M., Shino, M., Kuwabara, M., Takahashi, H., Matsuyama, T., Tada, H., Hagiwara, H., & Chikamatsu, K. (2025). Comparison of physician and large language model chatbot responses to online ear, nose, and throat inquiries. *Scientific Reports*, *15*(1), 21346. https://doi.org/10.1038/s41598-025-06769-1

174. Moya-Salazar, J., Salazar, C. R., & Goicochea-Palomino, E. A. (2024). Report of two cases of acute appendicitis prior to exams in medical students using OpenAI's ChatGPT. *Electronic Journal of General Medicine*, *21*(3), em585. https://doi.org/10.29333/ejgm/14571

175. Naeem, A., Khan, O., Baqir, S. M., Jana, K., Shankar, P., Kaur, A., Zaaya, M., Sajid, F., Mohsin, F., Boadla, M. R., Oo, A., Wong, V., Noor, M., Sandhu, S. P. S., Slobodyanuk, K., Shetty, V., & Tokayer, A. Z. (2025). Language artificial intelligence models as pioneers in diagnostic medicine? A retrospective analysis on real-time patients. *Journal of Clinical Medicine*, *14*(4), 1131. https://doi.org/10.3390/jcm14041131

176. Nastasi, A. J., Courtright, K. R., Halpern, S. D., & Weissman, G. E. (2023). A vignette-based evaluation of ChatGPT's ability to provide appropriate and equitable medical advice across care contexts. *Scientific Reports*, *13*(1), 17885. https://doi.org/10.1038/s41598-023-45223-y

177. Neo, J. R. E., Ser, J. S., & Tay, S. (2024). Use of large language model-based chatbots in managing the rehabilitation concerns and education needs of outpatient stroke survivors and caregivers. *Frontiers in Digital Health*, *6*, 1395501. https://doi.org/10.3389/fdgth.2024.1395501

178. Nickel, B., Ayre, J., Marinovich, M. L., Smith, D. P., Chiam, K., Lee, C. I., Wilt, T. J., Taba, M., Mccaffery, K., & Houssami, N. (2025). Are AI chatbots concordant with evidence-based cancer screening recommendations? *Patient Education and Counseling*, *134*, 108677. https://doi.org/10.1016/j.pec.2025.108677

179. Nitsch, K. E., & Ivatury, S. J. (2025). Do people prefer AI-generated patient educational materials over traditional ones? *Patient Education and Counseling*, *134*, 108672. https://doi.org/10.1016/j.pec.2025.108672

180. Norris, S. A., Kron, T., Masterson, M., & Badawy, M. K. (2025). An Australasian survey on the use of ChatGPT and other large language models in medical physics. *Physical and Engineering Sciences in Medicine*, *48*(3), 1145–1153. https://doi.org/10.1007/s13246-025-01571-9

181. Nov, O., Singh, N., & Mann, D. (2023). Putting ChatGPT's medical advice to the (Turing) test: Survey study. *JMIR Medical Education*, *9*, e46939. https://doi.org/10.2196/46939

182. Ozkan, E., Tekin, A., Ozkan, M. C., Cabrera, D., Niven, A., & Dong, Y. (2025). Global health care professionals' perceptions of large language model use in practice: Cross-sectional survey study. *JMIR Medical Education*, *11*, e58801. https://doi.org/10.2196/58801

183. Parekh, A. S., McCahon, J., Nghe, A., Pedowitz, D. I., Daniel, J. N., & Parekh, S. G. (2024). Foot and ankle patient education materials and artificial intelligence chatbots: A comparative analysis. *Foot & Ankle Specialist*, *19*(1), 92–100. https://doi.org/10.1177/19386400241235834

184. Patel, J., Robinson, P., Illing, E., & Anthony, B. (2024). Is ChatGPT 3.5 smarter than otolaryngology trainees? A comparison study of board style exam questions. *PLOS One*, *19*(9), e0306233. https://doi.org/10.1371/journal.pone.0306233

185. Paulo, S. F., Neto, I., Figueiredo, N. L., Lopes, D. S., & Nicolau, H. (2025). Reimagining multidisciplinary teams: Challenges and opportunities for LLMs in cancer MDTs. *Proceedings of the ACM on Human-Computer Interaction*, *9*(2), 1–22. https://doi.org/10.1145/3711055

186. Perlis, R. H., Goldberg, J. F., Ostacher, M. J., & Schneck, C. D. (2024). Clinical decision support for bipolar depression using large language models. *Neuropsychopharmacology*, *49*(9), 1412–1416. https://doi.org/10.1038/s41386-024-01841-2

187. Pham, J. H., Thongprayoon, C., Suppadungsuk, S., Miao, J., Craici, I. M., & Cheungpasitporn, W. (2024). Digital health tools in nephrology: A comparative analysis of AI and professional opinions via online polls. *Digital Health*, *10*, 20552076241277458. https://doi.org/10.1177/20552076241277458

188. Philuek, P., Kusump, S., Sathianpoonsook, T., Jansupom, C., Sawanyawisuth, P., Sawanyawisuth, K., & Chainarong, A. (2025). The effects of chat GPT generated exercise program in healthy overweight young adults: A pilot study. *Journal of Human Sport and Exercise*, *20*(1), 169. https://doi.org/10.55860/1epqgp77

189. Piazza, D., Martorana, F., Curaba, A., Sambataro, D., Valerio, M. R., Firenze, A., Pecorino, B., Scollo, P., Chiantera, V., Scibilia, G., Vigneri, P., Gebbia, V., & Scandurra, G. (2024). The consistency and quality of ChatGPT responses compared to clinical guidelines for ovarian cancer: A delphi approach. *Current Oncology*, *31*(5), 2796–2804. https://doi.org/10.3390/curroncol31050212

190. Picard, M., Leclercq, M., Bodein, A., Scott-Boyer, M. P., Perin, O., & Droit, A. (2025). Improving drug repositioning with negative data labeling using large language models. *Journal of Cheminformatics*, *17*(1), 16. https://doi.org/10.1186/s13321-025-00962-0

191. Platt, J., Nong, P. G., Smiddy, R., Hamasha, R., Clavijo, G. C., Richardson, J., & Kardia, S. L. R. (2024). Public comfort with the use of ChatGPT and expectations for healthcare. *Journal of the American Medical Informatics Association*, *31*(9), 1976–1982. https://doi.org/10.1093/jamia/ocae164

192. Pratibha, J. P., Prasad, S. S., & Manohar, N. (2023). Genital and extragenital lichen sclerosus et atrophicus: A case series written using ChatGPT. *Cureus*, *15*, e38987. https://doi.org/10.7759/cureus.38987

193. Qin, H., Zhu, Y., Jiang, Y., Luo, S., & Huang, C. (2024). Examining the impact of personalization and carefulness in AI-generated health advice: Trust, adoption, and insights in online healthcare consultations experiments. *Technology in Society*, *79*, 102726. https://doi.org/10.1016/j.techsoc.2024.102726

194. Qu, S. J., Liu, L., Zhou, M., Zhou, C. T., & Campy, K. S. (2024). Factors influencing Chinese doctors to use medical large language models. *Digital Health*, *10*, 20552076241297237. https://doi.org/10.1177/20552076241297237

195. Rackoff, G. N., Zhang, Z. Z., & Newman, M. G. (2025). Chatbot-delivered mental health support: Attitudes and utilization in a sample of US college students. *Digital Health*, *11*, 20552076241313401. https://doi.org/10.1177/20552076241313401

196. Rädel-Ablass, K., Schliz, K., Schlick, C., Meindl, B., Pahr-Hosbach, S., Schwendemann, H., Rupp, S., Roddewig, M., & Miersch, C. (2025). Teaching opportunities for anamnesis interviews through AI based teaching role plays: A survey with online learning students from health study programs. *BMC Medical Education*, *25*(1), 259. https://doi.org/10.1186/s12909-025-06756-0

197. Rames, M. M., O'Hern, K., Rames, J. D., Tooley, A. A., Hausauer, A. K., Munavalli, G. S., & Wyles, S. P. (2024). ChatGPT as a resource for patient education in cosmetic dermatological procedures: A boon or a bane? *Journal of Cosmetic Dermatology*, *23*(3), 1085–1086. https://doi.org/10.1111/jocd.16166

198. Rao, A. S., Kim, J., Mu, A., Young, C. C., Kalmowitz, E., Senter-Zapata, M., Whitehead, D. C., Garibyan, L., Landman, A. B., & Succi, M. D. (2025). Synthetic medical education in dermatology leveraging generative artificial intelligence. *npj Digital Medicine*, *8*(1), 247. https://doi.org/10.1038/s41746-025-01650-x

199. Rawashdeh, B., Kim, J., Alryalat, S. A., Prasad, R., & Cooper, M. (2023). ChatGPT and artificial intelligence in transplantation research: Is it always correct? *Cureus*, *15*, e42150. https://doi.org/10.7759/cureus.42150

200. Rebitschek, F. G., Carella, A., Kohlrausch-Pazin, S., Zitzmann, M., Steckelberg, A., & Wilhelm, C. (2025). Evaluating evidence-based health information from generative AI using a cross-sectional study with laypeople seeking screening information. *npj Digital Medicine*, *8*(1), 343. https://doi.org/10.1038/s41746-025-01752-6

201. Reinhardt, A., Matthes, J., Bojic, L., Maindal, H. T., Paraschiv, C., & Ryom, K. (2025). Help me, doctor AI? A cross-national experiment on the effects of disease threat and stigma on AI health information-seeking intentions. *Computers in Human Behavior*, *172*, 108718. https://doi.org/10.1016/j.chb.2025.108718

202. Reynolds, K., Nadelman, D., Durgin, J., Ansah-Addo, S., Cole, D., Fayne, R., Harrell, J., Ratycz, M., Runge, M., Shepard-Hayes, A., Wenzel, D., & Tejasvi, T. (2024). Comparing the quality of ChatGPT- and physician-generated responses to patients' dermatology questions in the electronic medical record. *Clinical and Experimental Dermatology*, *49*(7), 715–718. https://doi.org/10.1093/ced/llad456

203. Riddell, C. W., Chan, C., McGrinder, H., Earle, N. J., Poppe, K. K., & Doughty, R. N. (2023). College‐level reading is required to understand ChatGPT's answers to lay questions relating to heart failure. *European Journal of Heart Failure*, *25*(12), 2336–2337. https://doi.org/10.1002/ejhf.3083

204. Rizwan, A., & Sadiq, T. (2023). The use of AI in diagnosing diseases and providing management plans: A consultation on cardiovascular disorders with ChatGPT. *Cureus*, *15*, e43106. https://doi.org/10.7759/cureus.43106

205. Robinson, A., & Aggarwal, S. (2023). When precision meets penmanship: ChatGPT and surgery documentation. *Cureus*, *15*, e40546. https://doi.org/10.7759/cureus.40546

206. Rodler, S., Cei, F., Ganjavi, C., Checcucci, E., De Backer, P., Belenchon, I. R., Taratkin, M., Puliatti, S., Veccia, A., Piazza, P., Baekelandt, L., Kowalewski, K. F., Rivas, J. G., Fankhauser, C. D., Moschini, M., Gandaglia, G., Campi, R., Abreu, A. D., Russo, G. I., …, Collaborators, Y. A. U. (2025). GPT-4 generates accurate and readable patient education materials aligned with current oncological guidelines: A randomized assessment. *PLOS One*, *20*(6), e0324175. https://doi.org/10.1371/journal.pone.0324175

207. Rodriguez, D. V., Lawrence, K., Gonzalez, J., Brandfield-Harvey, B., Xu, L., Tasneem, S., Levine, D. L., & Mann, D. (2024). Leveraging generative AI tools to support the development of digital solutions in health care research: Case study. *JMIR Human Factors*, *11*, e52885. https://doi.org/10.2196/52885

208. Romoff, M., Brunette, M., Peterson, M. K., Hashmi, S. Z., & Kim, M. S. (2025). The role of large language models in improving the readability of orthopaedic spine patient educational material. *Journal of Orthopaedic Surgery and Research*, *20*(1), 531. https://doi.org/10.1186/s13018-025-05955-1

209. Roosan, D., Padua, P., Khan, R., Khan, H., Verzosa, C., & Wu, Y. (2024). Effectiveness of ChatGPT in clinical pharmacy and the role of artificial intelligence in medication therapy management. *Journal of the American Pharmacists Association*, *64*(2), 422–428.e8. https://doi.org/10.1016/j.japh.2023.11.023

210. Roy, J. M., Atallah, E., Piper, K., Majmundar, S., Mouchtouris, N., Self, D. M., Kaul, A., Sizdahkhani, S., Musmar, B., Tjoumakaris, S. I., Gooch, M. R., Rosenwasser, R. H., & Jabbour, P. M. (2025). Comparison of quality, empathy and readability of physician responses versus chatbot responses to common cerebrovascular neurosurgical questions on a social media platform. *Clinical Neurology and Neurosurgery*, *255*, 108986. https://doi.org/10.1016/j.clineuro.2025.108986

211. Ruiz Sarrias, O., Martínez del Prado, M. P., Sala Gonzalez, M. Á., Azcuna Sagarduy, J., Casado Cuesta, P., Figaredo Berjano, C., Galve-Calvo, E., López de San Vicente Hernández, B., López-Santillán, M., Nuño Escolástico, M., Sánchez Togneri, L., Sande Sardina, L., Pérez Hoyos, M. T., Abad Villar, M. T., Zabalza Zudaire, M., & Sayar Beristain, O. (2024). Leveraging large language models for precision monitoring of chemotherapy-induced toxicities: A pilot study with expert comparisons and future directions. *Cancers*, *16*(16), 2830. https://doi.org/10.3390/cancers16162830

212. Saeidnia, H. R., Kozak, M., Lund, B. D., & Hassanzadeh, M. (2024). Evaluation of ChatGPT's responses to information needs and information seeking of dementia patients. *Scientific Reports*, *14*(1), 10273. https://doi.org/10.1038/s41598-024-61068-5

213. Salam, B., Kravchenko, D., Nowak, S., Sprinkart, A. M., Weinhold, L., Odenthal, A., Mesropyan, N., Bischoff, L. M., Attenberger, U., Kuetting, D. L., Luetkens, J. A., & Isaak, A. (2024). Generative pre-trained transformer 4 makes cardiovascular magnetic resonance reports easy to understand. *Journal of Cardiovascular Magnetic Resonance*, *26*(1), 101035. https://doi.org/10.1016/j.jocmr.2024.101035

214. Sallam, M., Al-Mahzoum, K., Almutairi, Y. M., Alaqeel, O., Abu Salami, A., Almutairi, Z. E., Alsarraf, A. N., & Barakat, M. (2024). Anxiety among medical students regarding generative artificial intelligence models: A pilot descriptive study. *International Medical Education*, *3*(4), 406–425. https://doi.org/10.3390/ime3040031

215. Sallam, M., Al-Mahzoum, K., Alaraji, H., Albayati, N., Alenzei, S., AlFarhan, F., Alkandari, A., Alkhaldi, S., Alhaider, N., Al-Zubaidi, D., Shammari, F., Salahaldeen, M., Slehat, A. S., Mijwil, M. M., Abdelaziz, D. H., & Al-Adwan, A. S. (2025). Apprehension toward generative artificial intelligence in healthcare: A multinational study among health sciences students. *Frontiers in Education*, *10*, 1542769. https://doi.org/10.3389/feduc.2025.1542769

216. Samsonov, A. P., Habibi, A., Butler, J. J., Walls, R. J., & Kennedy, J. G. (2024). Artificial intelligence language models are useful tools for patients undergoing total ankle replacement. *Foot & Ankle Specialist*, *19*(2), 155–167. https://doi.org/10.1177/19386400241249810

217. Sanchez Tena, M. A., Alvarez-Peregrina, C., & Martinez-Perez, C. (2025). Evaluation of the perception of information from ChatGPT in myopia education: Perspectives of students and professionals. *Ophthalmic and Physiological Optics*, *45*(3), 883–894. https://doi.org/10.1111/opo.13451

218. Saraç, H., Ulusoy, T., Alpay, J., Ödemiş, H., & Söğüt, M. (2025). Evaluating the potential role of AI chatbots in designing personalized exercise programs for weight management. *International Journal of Human–Computer Interaction*, *41*(19), 12551–12558. https://doi.org/10.1080/10447318.2025.2462752

219. Schäfer, L. M., Krause, T., & Köhler, S. (2025). Exploring user characteristics, motives, and expectations and the therapeutic alliance in the mental health conversational AI clare®: A baseline study. *Frontiers in Digital Health*, *7*, 1576135. https://doi.org/10.3389/fdgth.2025.1576135

220. Schlarb, A. A., & Faber, J. (2024). Artificial intelligence (AI) in pediatric sleep: AI vs. expert-generated psychotherapeutic pediatric sleep stories. *Somnologie*. https://doi.org/10.1007/s11818-024-00491-2

221. Schmidt, M., Kafai, Y. B., Heinze, A., & Ghidinelli, M. (2024). Unravelling orthopaedic surgeons' perceptions and adoption of generative AI technologies. *Journal of CME*, *13*(1), 2437330. https://doi.org/10.1080/28338073.2024.2437330

222. Schmidt, H. G., Rotgans, J., & Mamede, S. (2025). Bias sensitivity in diagnostic decision-making: Comparing ChatGPT with residents. *Journal of General Internal Medicine*, *40*(4), 790–795. https://doi.org/10.1007/s11606-024-09177-9

223. Segal, S., & Khanna, A. K. (2023). Anesthetic management of a patient with juvenile hyaline fibromatosis: A case report written with the assistance of the large language model ChatGPT. *Cureus*, *15*, e35946. https://doi.org/10.7759/cureus.35946

224. Senoymak, M. C., Erbatur, N. H., Senoymak, I., & Firat, S. N. (2024). The role of artificial intelligence in endocrine management: Assessing ChatGPT's responses to prolactinoma queries. *Journal of Personalized Medicine*, *14*(4), 330. https://doi.org/10.3390/jpm14040330

225. Senturk, E., & Koparal, B. (2025). ChatGPT-4o vs psychiatrists in responding to common antidepressant concerns. *American Journal of Health Promotion*, *40*(1), 10–17. https://doi.org/10.1177/08901171251348208

226. Shahsavar, Y., & Choudhury, A. (2023). User intentions to use ChatGPT for self-diagnosis and health-related purposes: Cross-sectional survey study. *JMIR Human Factors*, *10*, e47564. https://doi.org/10.2196/47564

227. Sheikh, M. S., Dreesman, B., Barreto, E. F., Thongprayoon, C., Miao, J., Suppadungsuk, S., Mao, M. A., Qureshi, F., Pham, J. H., Craici, I. M., Kashani, K. B., & Cheungpasitporn, W. (2024). Identification of kidney-related medications using AI from self-captured pill images. *Renal Failure*, *46*(2), 2402075. https://doi.org/10.1080/0886022x.2024.2402075

228. Shikino, K., Shimizu, T., Otsuka, Y., Tago, M., Takahashi, H., Watari, T., Sasaki, Y., Iizuka, G., Tamura, H., Nakashima, K., Kunitomo, K., Suzuki, M., Aoyama, S., Kosaka, S., Kawahigashi, T., Matsumoto, T., Orihara, F., Morikawa, T., Nishizawa, T., …, Tokuda, Y. (2024). Evaluation of ChatGPT-generated differential diagnosis for common diseases with atypical presentation: Descriptive research. *JMIR Medical Education*, *10*, e58758. https://doi.org/10.2196/58758

229. Simsek, E., Kudube, A. I. A., & Sahin, R. S. (2025). The predictive effect of nursing students' attitudes and acceptance towards artificial intelligence on their clinical competencies. *Teaching and Learning in Nursing*, *20*(3), e806–e814. https://doi.org/10.1016/j.teln.2025.02.036

230. Sireci, F., Lorusso, F., Immordino, A., Centineo, M., Gerardi, I., Patti, G., Rusignuolo, S., Manzella, R., Gallina, S., & Dispenza, F. (2024). ChatGPT as a new tool to select a biological for chronic rhino sinusitis with polyps, "caution advised" or "distant reality"? *Journal of Personalized Medicine*, *14*(6), 563. https://doi.org/10.3390/jpm14060563

231. Small, W. R., Wiesenfeld, B., Brandfield-Harvey, B., Jonassen, Z., Mandal, S., Stevens, E. R., Major, V. J., Lostraglio, E., Szerencsy, A., Jones, S., Aphinyanaphongs, Y., Johnson, S. B., Nov, O., & Mann, D. (2024). Large language model-based responses to patients' in-basket messages. *JAMA Network Open*, *7*(7), e2422399. https://doi.org/10.1001/jamanetworkopen.2024.22399

232. Smith, A. M., Jacquez, E. A., & Argintar, E. H. (2024). Assessing the efficacy of an AI-powered chatbot (ChatGPT) in providing information on orthopedic surgeries: A comparative study with expert opinion. *Cureus*, *16*, e63287. https://doi.org/10.7759/cureus.63287

233. Solmonovich, R. L., Kouba, I., Lee, J. Y., Demertzis, K., & Blitz, M. J. (2025). Physician awareness of, interest in, and current use of artificial intelligence large language model-based virtual assistants. *PLOS One*, *20*(5), e0320749. https://doi.org/10.1371/journal.pone.0320749

234. Soto-Chávez, M. J., Bustos, M. M., Fernández-Avila, D. G., & Muñoz, O. M. (2024). Evaluation of information provided to patients by ChatGPT about chronic diseases in Spanish language. *Digital Health*, *10*, 20552076231224603. https://doi.org/10.1177/20552076231224603

235. Spatscheck, N., Schaschek, M., & Winkelmann, A. (2024). The effects of generative AI’s human-like competencies on clinical decision-making. *Journal of Decision Systems*, 1–39. https://doi.org/10.1080/12460125.2024.2430731

236. Spies, N. C., Hubler, Z., Roper, S. M., Omosule, C. L., Senter-Zapata, M., Roemmich, B. L., Brown, H. M., Gimple, R., & Farnsworth, C. W. (2023). GPT-4 underperforms experts in detecting IV fluid contamination. *The Journal of Applied Laboratory Medicine*, *8*(6), 1092–1100. https://doi.org/10.1093/jalm/jfad058

237. Spina, A. C., Fereydouni, P., Tang, J. N., Andalib, S., Picton, B. G., & Fox, A. R. (2025). Tailoring glaucoma education using large language models: Addressing health disparities in patient comprehension. *Medicine*, *104*(2), e41059. https://doi.org/10.1097/md.0000000000041059

238. Spotnitz, M., Idnay, B., Gordon, E. R., Shyu, R., Zhang, G. B., Liu, C., Cimino, J. J., & Weng, C. H. (2024). A survey of clinicians' views of the utility of large language models. *Applied Clinical Informatics*, *15*(02), 306–312. https://doi.org/10.1055/a-2281-7092

239. Stage, M. A., Creamer, M. M., & Ruben, M. A. (2025). "Having providers WHO are trained and have empathy is life-saving": Improving primary care communication through thematic analysis with ChatGPT and human expertise. *PEC Innovation*, *6*, 100371. https://doi.org/10.1016/j.pecinn.2024.100371

240. Stalp, J. L., Denecke, A., Jentschke, M., Hillemanns, P., & Klapdor, R. (2024). Quality of ChatGPT-generated therapy recommendations for breast cancer treatment in gynecology. *Current Oncology*, *31*(7), 3845–3854. https://doi.org/10.3390/curroncol31070284

241. Stanceski, K., Zhong, S., Zhang, X., Khadra, S., Tracy, M., Koria, L., Lo, S., Naganathan, V., Kim, J., Dunn, A. G., & Ayre, J. (2024). The quality and safety of using generative AI to produce patient-centred discharge instructions. *npj Digital Medicine*, *7*(1), 329. https://doi.org/10.1038/s41746-024-01336-w

242. Stephan, D., Bertsch, A. S., Schumacher, S., Puladi, B., Burwinkel, M., Al-Nawas, B., Kämmerer, P. W., & Thiem, D. G. E. (2025). Improving patient communication by simplifying AI-generated dental radiology reports with ChatGPT: Comparative study. *Journal of Medical Internet Research*, *27*, e73337. https://doi.org/10.2196/73337

243. Stoneham, S., Livesey, A., Cooper, H., & Mitchell, C. (2024). ChatGPT versus clinician: Challenging the diagnostic capabilities of artificial intelligence in dermatology. *Clinical and Experimental Dermatology*, *49*(7), 707–710. https://doi.org/10.1093/ced/llad402

244. Sultan, I., Al-Abdallat, H., Alnajjar, Z., Ismail, L., Abukhashabeh, R., Bitar, L., & Abu Shanap, M. (2023). Using ChatGPT to predict cancer predisposition genes: A promising tool for pediatric oncologists. *Cureus*, *15*, e47594. https://doi.org/10.7759/cureus.47594

245. Sun, X., Liu, Y., Bosch, J. A., & Li, Z. (2025). Interface matters: Exploring human trust in health information from large language models via text, speech, and embodiment. *Proceedings of the ACM on Human-Computer Interaction*, *9*(2), 1–32. https://doi.org/10.1145/3711014

246. Taha, T. A. A., Abdel-Qader, D. H., Alamiry, K. R., Fadl, Z. A., Alrawi, A., & Abdelsattar, N. K. (2024). Perception, concerns, and practice of ChatGPT among egyptian pharmacists: A cross-sectional study in egypt. *BMC Health Services Research*, *24*(1), 1500. https://doi.org/10.1186/s12913-024-11815-1

247. Tai-Seale, M., Baxter, S. L., Vaida, F., Walker, A., Sitapati, A. M., Osborne, C., Diaz, J., Desai, N., Webb, S., Polston, G., Helsten, T., Gross, E., Thackaberry, J., Mandvi, A., Lillie, D., Li, S., Gin, G., Achar, S., Hofflich, H., …, Longhurst, C. A. (2024). AI-generated draft replies integrated into health records and physicians' electronic communication. *JAMA Network Open*, *7*(4), e246565. https://doi.org/10.1001/jamanetworkopen.2024.6565

248. Tangadulrat, P., Sono, S., & Tangtrakulwanich, B. (2023). Using ChatGPT for clinical practice and medical education: Cross-sectional survey of medical students' and physicians' perceptions. *JMIR Medical Education*, *9*, e50658. https://doi.org/10.2196/50658

249. Tao, W. J., Yang, J. M., & Qu, X. (2024). Utilization of, perceptions on, and intention to use AI chatbots among medical students in China: National cross-sectional study. *JMIR Medical Education*, *10*, e57132. https://doi.org/10.2196/57132

250. Tekin, M., Yurdal, M. O., Toraman, Ç., Korkmaz, G., & Uysal, I. (2025). Is AI the future of evaluation in medical education?? AI vs. human evaluation in objective structured clinical examination. *BMC Medical Education*, *25*(1), 641. https://doi.org/10.1186/s12909-025-07241-4

251. Tenner, Z. M., Cottone, M. C., & Chavez, M. R. (2024). Harnessing the open access version of ChatGPT for enhanced clinical opinions. *PLOS Digital Health*, *3*(2), e0000355. https://doi.org/10.1371/journal.pdig.0000355

252. Thota, D., Alt, D., Cole, J., & Tring, V. S. (2025). Prompting pro tips! Best practices for generating clinical narrative summaries. *Military Medicine*, *191*(1-2), e445–e448. https://doi.org/10.1093/milmed/usaf247

253. Tong, L., Niu, Y. R., Zhou, L. X., Jin, S., Wang, Y. L., & Xiao, Q. (2025). Perceptions and experiences of generative artificial intelligence training to support research for Chinese nurses: A qualitative focus group study. *International Journal of Nursing Sciences*, *12*(3), 210–217. https://doi.org/10.1016/j.ijnss.2025.04.010

254. Tortella, F., Palese, A., Turolla, A., Castellini, G., Pillastrini, P., Landuzzi, M. G., Cook, C., Galeoto, G., Giovannico, G., Rodeghiero, L., Gianola, S., & Rossettini, G. (2025). Knowledge and use, perceptions of benefits and limitations of artificial intelligence chatbots among Italian physiotherapy students: A cross-sectional national study. *BMC Medical Education*, *25*(1), 572. https://doi.org/10.1186/s12909-025-07176-w

255. Triantafyllopoulos, L., Feretzakis, G., Tzelves, L., Sakagianni, A., Verykios, V. S., & Kalles, D. (2024). Evaluating the interactions of medical doctors with chatbots based on large language models: Insights from a nationwide study in the greek healthcare sector using ChatGPT. *Computers in Human Behavior*, *161*, 108404. https://doi.org/10.1016/j.chb.2024.108404

256. Triplett, S., Ness-Engle, G. L., & Behnen, E. M. (2025). A comparison of drug information question responses by a drug information center and by ChatGPT. *American Journal of Health-System Pharmacy*, *82*(8), 448–460. https://doi.org/10.1093/ajhp/zxae316

257. Tuncer, G. Z., & Tuncer, M. (2024). Investigation of nurses' general attitudes toward artificial intelligence and their perceptions of ChatGPT usage and influencing factors. *Digital Health*, *10*, 20552076241277025. https://doi.org/10.1177/20552076241277025

258. Tuppal, C. P., Ninobla, M. M. G., Loresco, R. D., Cabradilla, M. R., Tuppal, S. M. P., Pellacoeur, L. K. A., Roa, M. N. T., Chan, J. M. A. R., Panes, I. I., Ferares, A. L. U., & Caspillo, I. J. (2025). Looking into the future of ChatGPT and its cultural consideration from Filipino nurse scientists' perspectives: A narrative-focused group discussion. *Creative Nursing*, *31*(3), 318–329. https://doi.org/10.1177/10784535251324516

259. Uribe, S. E., Maldupa, I., Kavadella, A., El Tantawi, M., Chaurasia, A., Fontana, M., Marino, R., Innes, N., & Schwendicke, F. (2024). Artificial intelligence chatbots and large language models in dental education: Worldwide survey of educators. *European Journal of Dental Education*, eje.13009. https://doi.org/10.1111/eje.13009

260. Valetopoulou, A., Williams, S., & Marcus, H. J. (2024). Can artificial intelligence improve medicine’s uncomfortable relationship with maths? *npj Digital Medicine*, *7*(1), 166. https://doi.org/10.1038/s41746-024-01168-8

261. Vasudevan, R., Alqahtani, T., Alqahtani, S., Devanandan, P., Kandasamy, G., Saad, R., Amer, A., Abduallah, R., Waleed, G., Hasan, R., & Ahmed, L. (2025). Integrating AI in healthcare education: Attitudes of pharmacy students at King Khalid University towards using ChatGPT in clinical decision-making. *Healthcare*, *13*(11), 1265. https://doi.org/10.3390/healthcare13111265

262. Verran, C. (2024). Artificial intelligence-generated patient information leaflets: A comparison of contents according to british association of dermatologists standards. *Clinical and Experimental Dermatology*, *49*(7), 711–714. https://doi.org/10.1093/ced/llad461

263. Vidal Sabanés, L., & da Cunha, I. (2025). AI as a resource for the clarification of medical terminology: An analysis of its advantages and limitations. *Terminology. International Journal of Theoretical and Applied Issues in Specialized Communication*, *31*(1), 37–71. https://doi.org/10.1075/term.00083.vid

264. von Bubnoff, F., Werner, J., Hebach, N. R., Chopra, D. A., & Schubert, M. C. (2025). Medical mistrust in online cancer communities: A large-scale analysis across 10 cancer entities. *Psycho-Oncology*, *34*(6), e70180. https://doi.org/10.1002/pon.70180

265. Vu, C. B., & Park, D. Y. (2025). Why would university students use ChatGPT for health discourses? A moderated mediation analysis. *Journal of Consumer Health on the Internet*, *29*(2), 149–173. https://doi.org/10.1080/15398285.2025.2491042

266. Wang, Y., & Li, S. Y. (2024). Tech vs. tradition: ChatGPT and mindfulness in enhancing older adults' emotional health. *Behavioral Sciences*, *14*(10), 923. https://doi.org/10.3390/bs14100923

267. Wiemken, T. L., & Carrico, R. M. (2024). Assisting the infection preventionist: Use of artificial intelligence for health care-associated infection surveillance. *American Journal of Infection Control*, *52*(6), 625–629. https://doi.org/10.1016/j.ajic.2024.02.007

268. Wu, P. F., Summers, C., Panesar, A., Kaura, A., & Zhang, L. (2024). AI hesitancy and acceptability-perceptions of AI chatbots for chronic health management and long COVID support: Survey study. *JMIR Human Factors*, *11*, e51086. https://doi.org/10.2196/51086

269. Wu, H., Li, M. Y., & Zhang, L. (2025). Comparing physician and large language model responses to influenza patient questions in the online health community. *International Journal of Medical Informatics*, *197*, 105836. https://doi.org/10.1016/j.ijmedinf.2025.105836

270. Xia, D. K., Song, M. Y., & Zhu, T. S. (2025). A comparison of the persuasiveness of human and ChatGPT generated pro-vaccine messages for HPV. *Frontiers in Public Health*, *12*, 1515871. https://doi.org/10.3389/fpubh.2024.1515871

271. Xie, Z. H. (2025). WHO is spreading AI-generated health rumors? A study on the association between AIGC interaction types and the willingness to share health rumors. *Social Media + Society*, *11*(1), 20563051251323391. https://doi.org/10.1177/20563051251323391

272. Xu, M., Lotke, P., Figueroa, M., Doty, N., & Baum, J. (2025). Reproductive health and ChatGPT: An evaluation of AI-generated responses to commonly asked abortion questions. *Culture, Health & Sexuality*, 1–13. https://doi.org/10.1080/13691058.2025.2517289

273. Yan, Z. L., Liu, J. W., Fan, Y. H., Lu, S. Y., Xu, D. T., Yang, Y., Wang, H. G., Mao, J., Tseng, H. C., Chang, T. H., & Chen, Y. (2025). Ability of ChatGPT to replace doctors in patient education: Cross-sectional comparative analysis of inflammatory bowel disease. *Journal of Medical Internet Research*, *27*, e62857. https://doi.org/10.2196/62857

274. Yanagita, Y., Yokokawa, D., Uchida, S., Li, Y., Uehara, T., & Ikusaka, M. (2024). Can AI-generated clinical vignettes in Japanese be used medically and linguistically? *Journal of General Internal Medicine*, *39*(16), 3282–3289. https://doi.org/10.1007/s11606-024-09031-y

275. Yang, X., Xiao, Y., Liu, D., Zhang, Y., Deng, H., Huang, J., Shi, H., Liu, D., Liang, M., Jin, X., Sun, Y., Yao, J., Zhou, X., Guo, W., He, Y., Tang, W., & Xu, C. (2025). Enhancing doctor-patient communication using large language models for pathology report interpretation. *BMC Medical Informatics and Decision Making*, *25*(1), 36. https://doi.org/10.1186/s12911-024-02838-z

276. Yang, T. T., Zheng, H. X., Cao, S., Jing, M. L., Hu, J., Zuo, Y., Chen, Q. Y., & Zhang, J. J. (2025). Harnessing an artificial intelligence-based large language model with personal health record capability for personalized information support in postsurgery myocardial infarction: Descriptive qualitative study. *Journal of Medical Internet Research*, *27*, e68762. https://doi.org/10.2196/68762

277. Yang, X., Zhang, Y., Jiang, J., Chen, Z., Bai, R., Yuan, Z., Dong, L., Xiao, Y., Liu, D., Deng, H., Huang, J., Shi, H., Liu, D., Liang, M., Tang, W., & Xu, C. (2025). Harnessing GPT-4 for automated error detection in pathology reports: Implications for oncology diagnostics. *Digital Health*, *11*, 20552076251346703. https://doi.org/10.1177/20552076251346703

278. Yazla, M., & Sarcan, E. (2025). Compliance evaluation with ChatGPT for diagnosis and treatment in patients brought to the ED with a preliminary diagnosis of stroke. *Prehospital Emergency Care*, *29*(3), 243–251. https://doi.org/10.1080/10903127.2025.2475513

279. Yigit, S., Berse, S., Dirgar, E., & Güner, S. G. (2025). Views of health sciences undergraduates on ChatGPT, an artificial intelligence-powered language model: A qualitative study. *Innovations in Education and Teaching International*, *62*(4), 1258–1272. https://doi.org/10.1080/14703297.2024.2391044

280. Yildiz, M. S., & Alper, A. (2023). Can ChatGPT-4 diagnose in Turkish: A comparison of ChatGPT responses to health-related questions in English and Turkish. *Journal of Consumer Health on the Internet*, *27*(3), 294–307. https://doi.org/10.1080/15398285.2023.2240652

281. Ying, L., Li, S., Chen, C., Yang, F., Li, X., Chen, Y., Ding, Y., Chang, G., Li, J., & Wang, X. (2024). Screening/diagnosis of pediatric endocrine disorders through the artificial intelligence model in different language settings. *European Journal of Pediatrics*, *183*(6), 2655–2661. https://doi.org/10.1007/s00431-024-05527-1

282. Yun, H. S., & Bickmore, T. (2025). Online health information-seeking in the era of large language models: Cross-sectional web-based survey study. *Journal of Medical Internet Research*, *27*, e68560. https://doi.org/10.2196/68560

283. Zeng, S.-Y., Kong, Q.-Z., Wu, X.-Q., Ma, T., Wang, L.-M., Xu, L.-Q., Kou, G.-J., Zhang, M.-M., Yang, X.-Y., Zuo, X.-L., Li, Y.-Y., & Li, Y.-Q. (2024). Artificial intelligence-generated patient education materials for Helicobacter pylori infection: A comparative analysis. *Helicobacter*, *29*(4), e13115. https://doi.org/10.1111/hel.13115

284. Zhang, J. X., Ma, Y., Zhang, R., Chen, Y. H., Xu, M. Y., Su, R. N., & Ma, K. (2024). A comparative study of GPT-4o and human ophthalmologists in glaucoma diagnosis. *Scientific Reports*, *14*(1), 30385. https://doi.org/10.1038/s41598-024-80917-x

285. Zhang, D., & Zhao, X. M. (2024). Understanding adoption intention of virtual medical consultation systems: Perceptions of ChatGPT and satisfaction with doctors. *Computers in Human Behavior*, *159*, 108359. https://doi.org/10.1016/j.chb.2024.108359

286. Zhou, J. C., Zhang, J. T., Wan, R. R., Cui, X. C., Liu, Q. Y., Guo, H., Shi, X. F., Fu, B. B., Meng, J., Yue, B., Zhang, Y. Y., & Zhang, Z. Y. (2025). Integrating AI into clinical education: Evaluating general practice trainees' proficiency in distinguishing AI-generated hallucinations and impacting factors. *BMC Medical Education*, *25*(1), 406. https://doi.org/10.1186/s12909-025-06916-2

**Supplementary Table S2**

*Coding Framework and Operational Definitions (N = 286)*

| **Variable or concept** | **Coding values** | **Operational definition** | **Krippendorff’s α** |
| --- | --- | --- | --- |
| **Part 1. Study characteristics** | | | |
| **Human involvement** | 1 = none; 2 = evaluator or comparator only; 3 = participant | 1 = None: no person provides data; the analysis concerns chatbot outputs, records, documents, cases, or question banks.  2 = Evaluator or comparator only: people rate chatbot outputs or serve as expert comparators, but their own perceptions, behavior, use, or outcomes are not analysed.  3 = Participant: people were themselves an object of measurement, with findings reported about their perceptions, attitudes, intentions, reported or observed use, etc. | 1 |
| **Country of study participants** | Country; NA (non-human-subject study); Unspecified | Country was recorded only for studies in which people participated by providing data. *NA* was used for studies with no human involvement or with evaluators or raters only; *Unspecified* was used when participant country was not reported. | 1 |
| **Health topic** | | |  |
| **Communicable or infectious diseases** | 1 | Infectious agents, transmission, prevention, diagnosis, treatment, or management of communicable diseases, including infection-specific vaccination contexts. | 0.95 |
| **Noncommunicable or chronic diseases** | 2 | Noninfectious diseases and long-term conditions, including cancer, cardiovascular, metabolic, neurological, musculoskeletal, ophthalmic, dental, and other chronic or specialty-care conditions. |  |
| **Mental health** | 3 | Mental or behavioral health conditions, counseling, psychological well-being, distress, stigma, or emotional and psychosocial support when these formed the primary health domain. |  |
| **Maternal, sexual, and reproductive health** | 4 | Pregnancy, childbirth, maternal health, fertility, contraception, sexual health, reproductive health, or related conditions. |  |
| **Nutrition and lifestyle-related issues** | 5 | Diet, physical activity, weight management, sleep, or other lifestyle-related health issues when no more specific disease category predominated. |  |
| **General health information** | 6 | Broad, cross-domain, or generic health information, health-service, or health-AI questions for which no single disease or specialty was the primary focus. |  |
| **Occupational health** | 7 | Health, safety, exposure, or well-being specifically associated with work or an occupational setting. |  |
| **Other health topic** | 8 (specify) | Health topics that could not be assigned to any predefined coding category were recorded in the accompanying free-text field. |  |
| **Methodological approach** | | | |
| **Interview** | 0 = absent; 1 = present | An explicitly qualitative, semi-structured, in-depth, or narrative interview used to collect participant data. An expert panel that only rated chatbot outputs was treated as a measurement procedure and was not coded as an interview. | 1 |
| **Focus group** | 0 = absent; 1 = present | A facilitated group discussion in which interaction among participants contributed to the qualitative data. | 1 |
| **Text analysis** | 0 = absent; 1 = present | A distinct computational, linguistic, lexical, semantic, sentiment, readability, or related analysis of text. | 1 |
| **Delphi study** | 0 = absent; 1 = present | An explicit iterative, multi-round expert-consensus process involving controlled feedback or reconsideration across rounds. A one-time expert survey or rating panel was not coded as Delphi. | Not estimable  (100% agreement) |
| **Experiment** | 0 = absent; 1 = present | Human participants were assigned to or exposed to researcher-controlled conditions or completed a manipulated comparison of conditions. | 1 |
| **Survey** | 0 = absent; 1 = present | An independent participant survey component administered to elicit participants' own reports. | 1 |
| **Content analysis** | 0 = absent; 1 = present | Systematic coding, categorization, rubric or reference-standard scoring, correctness judgment, or other substantive qualitative or quantitative assessment of independently analyzed chatbot outputs, chatbot logs, or documents. | 1 |
| **Network analysis** | 0 = absent; 1 = present | A formal analysis of relational data represented through nodes, links, ties, or network structure. | Not estimable  (100% agreement) |
| **Chatbot and model characteristics** | | | |
| **Chatbot modality** | Text-based; Multimodal; Voice-based; Unspecified / not reported | The interaction channel actually evaluated or reported. Text-based denotes text input and output even when the underlying system was technically multimodal. Multimodal combines text with at least one non-text channel, such as images or voice. Voice-based denotes voice-only or primarily speech-based interaction. Missing channel information was coded Unspecified | 1 |
| **LLM type** | General-purpose LLM; Custom LLM application; Unspecified | General-purpose denotes direct use of a broadly available consumer system, such as ChatGPT, Claude, Gemini, or Copilot, including use with medical prompts.  Custom requires an explicitly built or adapted application, API pipeline, retrieval-augmented generation, fine-tuning, tool integration, or domain-specific system. Unspecified denotes that the article did not provide sufficient information to classify the LLM type. | 0.81 |
| **Evidence-type** | | |  |
| **Output evaluation** | 0 = absent; 1 = present | Researchers generated or selected chatbot outputs and assessed output or task performance using substantive criteria, including accuracy, safety, completeness, readability, guideline concordance, or agreement with a reference standard. | 1 |
| **Self-reported perceptions and use** | 0 = absent; 1 = present | Participants reported attitudes, trust, acceptance, perceived usefulness, intentions, prior or current chatbot use. | 1 |
| **Researcher-directed interaction** | 0 = absent; 1 = present | The protocol required or enabled participants to enter information and receive contingent chatbot responses, including experimental tasks, interventions, usability tests, and assigned home use. | 0.85 |
| **Documented naturalistic use** | 0 = absent; 1 = present | Personal, clinical, organizational, or scholarly chatbot use occurred outside researcher direction and was documented through logs, electronic health record or audit trails, utilization or conversation records, reproduced interaction records, or direct observation. | Not estimable  (100% agreement) |
| **Part 2. Stakeholder groups** | | | |
| **Public or patient** | 0 = absent; 1 = present | Patients, caregivers or family members, members of the public, or general participants acting as users or intended users of health information, self-care, preventive care, recovery support, or emotional support. | 1 |
| **Health professionals** | 0 = absent; 1 = present | Practicing clinicians, nurses, pharmacists, allied-health staff, residents, or public-health practitioners acting in a professional capacity. | 1 |
| **Health researchers and students** | 0 = absent; 1 = present | Research staff or faculty and health-related students acting as researchers, learners, or trainees. | 0.92 |
| **Health institutions** | 0 = absent; 1 = present | A health organization or system was a decision-maker, implementation actor, organizational workflow target, or unit of analysis. | 0.83 |
| **Part 3. Purpose categories** | | | |
| **Seeking health information** | 0 = absent; 1 = present | Obtaining, explaining, simplifying, translating, or checking health information for members of the public or patients. | 1 |
| **Symptom assessment and self-care management** | 0 = absent; 1 = present | Symptom assessment, self-diagnosis, self-triage, decisions about seeking care, chronic-disease self-management, symptom monitoring, medication self-management, or related self-care. | 1 |
| **Emotional support** | 0 = absent; 1 = present | Mental-health counseling, companionship, psychosocial support, emotional expression, or support relating to stress, anxiety, depression, loneliness, or other emotional needs. | 1 |
| **Preventive and transitional care support** | 0 = absent; 1 = present | Preventive guidance or behavior change, screening or vaccination, or support during postoperative, rehabilitative, discharge, and broader recovery periods. | Not estimable  (100% agreement) |
| **Clinical decision support** | 0 = absent; 1 = present | Processing clinical information, diagnostic or professional triage reasoning, treatment selection, care planning, risk assessment, or other decisions made in a health-professional role. | 0.92 |
| **Improving administrative efficiency** | 0 = absent; 1 = present | Clinical documentation, report or note generation, scheduling, referrals, inbox work, coding, or other administrative workload and efficiency tasks. | 1 |
| **Supporting patient interaction and doctor–patient communication** | 0 = absent; 1 = present | Clinician replies to patient inquiries, patient-facing explanations or education produced for professional use, consultation communication, or follow-up communication. | 1 |
| **Enhancing continuing education** | 0 = absent; 1 = present | Health-professional or student education, training, simulation, assessment, knowledge acquisition, or professional development. | 0.90 |
| **Facilitating health research** | 0 = absent; 1 = present | Literature or guideline synthesis, scholarly writing, research-data analysis, research design, or reported use of chatbots for health-research tasks. | 0.89 |
| **Institutional workflow support and care navigation** | 0 = absent; 1 = present | A health-organization or health-system workflow, including organization-level implementation, care navigation, communication, documentation, or operational support. | Not estimable  (100% agreement) |

*Note.* Intercoder reliability was assessed using 30 articles independently coded by two researchers before consensus reconciliation. Krippendorff’s α was calculated using the nominal metric. For variables showing no variation in the reliability subset, α was not estimable; percent agreement is reported instead.

**Supplementary Table S3**

*Database Search Strategies*

| **Platform/database** | **Filters** | **Search string** |
| --- | --- | --- |
| EBSCOhost: Academic Search Complete; Communication & Mass Media Complete; Global Health; Library Literature & Information Science; APA PsycInfo; Education Source Ultimate | Full Text; Scholarly (Peer Reviewed) Journals; Academic Journals; English | AB ("generative artificial intelligence" OR "generative AI" OR "AI-generated content" OR AIGC OR GenAI OR "large language model*" OR Chatbot OR ChatGPT OR DeepSeek OR Midjourney)  AND  AB (health* OR healthcare OR medicine OR medical OR clinic* OR patient* OR hospital* OR doctor* OR "public health" OR "chronic disease*" OR "mental health") |
| PubMed | Full text; English; Exclude preprints | ("generative artificial intelligence"[Title/Abstract] OR "generative AI"[Title/Abstract] OR "AI-generated content"[Title/Abstract] OR AIGC[Title/Abstract] OR GenAI[Title/Abstract] OR "large language model*"[Title/Abstract] OR Chatbot[Title/Abstract] OR ChatGPT[Title/Abstract] OR DeepSeek[Title/Abstract] OR Midjourney[Title/Abstract])  AND  (health*[Title/Abstract] OR healthcare[Title/Abstract] OR medicine[Title/Abstract] OR medical[Title/Abstract] OR clinic*[Title/Abstract] OR patient*[Title/Abstract] OR hospital*[Title/Abstract] OR doctor*[Title/Abstract] OR "public health"[Title/Abstract] OR "chronic disease*"[Title/Abstract] OR "mental health"[Title/Abstract]) |
| Web of Science Core Collection | English; Article and Proceedings Paper; excluded Computer Science and Engineering research areas | AB=("generative artificial intelligence" OR "generative AI" OR "AI-generated content" OR AIGC OR GenAI OR "large language model*" OR Chatbot OR ChatGPT OR DeepSeek OR Midjourney)  AND  AB=(health* OR healthcare OR medicine OR medical OR clinic* OR patient* OR hospital* OR doctor* OR "public health" OR "chronic disease*" OR "mental health") |
| Scopus | English; Article and Conference Paper; Journal and Conference Proceeding; Social Sciences, Health Professions, and Arts and Humanities | ABS("generative artificial intelligence" OR "generative AI" OR "AI-generated content" OR AIGC OR GenAI OR "large language model*" OR Chatbot OR ChatGPT OR DeepSeek OR Midjourney)  AND  ABS(health* OR healthcare OR medicine OR medical OR clinic* OR patient* OR hospital* OR doctor* OR "public health" OR "chronic disease*" OR "mental health") |

*Note.* Searches were conducted in July 2025. AI-related terms and health-related terms were combined using AND, with synonyms within each concept block combined using OR. The six EBSCOhost databases were searched simultaneously.

**Supplementary Table S4**

*Descriptive Distribution of Health Domains and LLM Types Across Stakeholder Groups*

| **Subgroup** | **Overall (N = 286)** | **General public or patients (n = 138)** | **Health professionals (n = 125)** | **Health researchers and students (n = 46)** | **Health institutions (n = 8)** |
| --- | --- | --- | --- | --- | --- |
| **Health domain** |  |  |  |  |  |
| Communicable or infectious diseases | 17 (5.9) | 10 (7.2) | 8 (6.4) | 0 (0.0) | 0 (0.0) |
| General health information | 103 (36.0) | 37 (26.8) | 47 (37.6) | 26 (56.5) | 5 (62.5) |
| Maternal, sexual, and reproductive health | 11 (3.8) | 9 (6.5) | 0 (0.0) | 2 (4.3) | 0 (0.0) |
| Mental health | 30 (10.5) | 20 (14.5) | 8 (6.4) | 3 (6.5) | 1 (12.5) |
| Noncommunicable or chronic diseases | 115 (40.2) | 56 (40.6) | 60 (48.0) | 13 (28.3) | 1 (12.5) |
| Nutrition- and lifestyle-related issues | 4 (1.4) | 4 (2.9) | 0 (0.0) | 0 (0.0) | 0 (0.0) |
| Occupational health | 1 (0.3) | 0 (0.0) | 0 (0.0) | 1 (2.2) | 0 (0.0) |
| Other health topics | 5 (1.7) | 2 (1.4) | 2 (1.6) | 1 (2.2) | 1 (12.5) |
| **LLM type** |  |  |  |  |  |
| Custom LLM application | 15 (5.2) | 8 (5.8) | 6 (4.8) | 2 (4.3) | 6 (75.0) |
| General-purpose LLM | 267 (93.4) | 129 (93.5) | 117 (93.6) | 44 (95.7) | 1 (12.5) |
| Unspecified | 4 (1.4) | 1 (0.7) | 2 (1.6) | 0 (0.0) | 1 (12.5) |

***Note.*** Values are n (%). Percentages in the overall column were calculated using the full sample (N = 286), whereas percentages in the stakeholder columns were calculated within the corresponding stakeholder group. Stakeholder groups were not mutually exclusive; an article could therefore appear in more than one stakeholder column.
